# A socio‐ecological model for predicting impacts of land‐use and climate change on regional plant diversity in the Austrian Alps

**DOI:** 10.1111/gcb.14977

**Published:** 2020-01-29

**Authors:** Iwona Dullinger, Andreas Gattringer, Johannes Wessely, Dietmar Moser, Christoph Plutzar, Wolfgang Willner, Claudine Egger, Veronika Gaube, Helmut Haberl, Andreas Mayer, Andreas Bohner, Christian Gilli, Kathrin Pascher, Franz Essl, Stefan Dullinger

**Affiliations:** ^1^ Division of Conservation Biology, Vegetation and Landscape Ecology Department of Botany and Biodiversity Research University of Vienna Vienna Austria; ^2^ Institute of Social Ecology Department of Economics and Social Sciences University of Natural Resources and Life Sciences, Vienna Vienna Austria; ^3^ Agricultural Research and Education Centre Raumberg‐Gumpenstein Irdning‐Donnersbachtal Austria; ^4^ Division of Systematic and Evolutionary Botany Department of Botany and Biodiversity Research University of Vienna Vienna Austria; ^5^ Institute of Zoology Department of Integrative Biology and Biodiversity Research University of Natural Resources and Life Sciences, Vienna Vienna Austria

**Keywords:** agent‐based model, biodiversity, climate change, Europe, global change, land‐use change, plant diversity, plant species distribution, species distribution model

## Abstract

Climate and land‐use change jointly affect the future of biodiversity. Yet, biodiversity scenarios have so far concentrated on climatic effects because forecasts of land use are rarely available at appropriate spatial and thematic scales. Agent‐based models (ABMs) represent a potentially powerful but little explored tool for establishing thematically and spatially fine‐grained land‐use scenarios. Here, we use an ABM parameterized for 1,329 agents, mostly farmers, in a Central European model region, and simulate the changes to land‐use patterns resulting from their response to three scenarios of changing socio‐economic conditions and three scenarios of climate change until the mid of the century. Subsequently, we use species distribution models to, first, analyse relationships between the realized niches of 832 plant species and climatic gradients or land‐use types, respectively, and, second, to project consequent changes in potential regional ranges of these species as triggered by changes in both the altered land‐use patterns and the changing climate. We find that both drivers determine the realized niches of the studied plants, with land use having a stronger effect than any single climatic variable in the model. Nevertheless, the plants' future distributions appear much more responsive to climate than to land‐use changes because alternative future socio‐economic backgrounds have only modest impact on land‐use decisions in the model region. However, relative effects of climate and land‐use changes on biodiversity may differ drastically in other regions, especially where landscapes are still dominated by natural or semi‐natural habitat. We conclude that agent‐based modelling of land use is able to provide scenarios at scales relevant to individual species distribution and suggest that coupling ABMs with models of species' range change should be intensified to provide more realistic biodiversity forecasts.

## Graphical abstract

Land‐use and climate change will have important effects on the future of biodiversity. We predict changes to the potential distribution of vascular plants in response to these two drivers in a region of the Austrian Alps. We therefore combine agent‐based modelling of land owners' decisions with species distribution modelling. We find that both drivers determine the realized niches of the studied plants, with land use having a stronger effect than any single climatic variable in the model. Nevertheless, the plants' future distributions appear more responsive to climate than to land‐use changes.

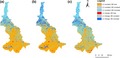

## 1 INTRODUCTION

Land use is considered the most important human threat to terrestrial biodiversity (Marques et al., 2019; Maxwell, Fuller, Brooks, & Watson, 2016; Newbold et al., 2015; Pimm & Raven, 2000). Land use potentially degrades or destroys natural ecosystems and increases the fragmentation of natural or semi‐natural habitats. Negative impacts of that kind are already widespread and currently affect approximately three quarters of the earth's ice‐free land mass (Ellis et al., 2013; Erb et al., 2007). They are predicted to further rise in extent and intensity, driven by the rapidly growing human population, economic growth, as well as changes in lifestyle and diets (Erb et al., 2016; Foley et al., 2011; Tilman, Balzer, Hill, & Befort, 2011).

However, human agency threatens biodiversity in multiple ways (Ehrlich & Pringle, 2008; Vitousek, Mooney, Lubchenco, & Melillo, 1997). Besides habitat conversion through land use, climate change is considered another particularly powerful driver of biodiversity loss (Pereira et al., 2010). Climate change forces species to either adapt to the changing conditions or to shift their distribution to more suitable environments (e.g. cooler ones, Bellard, Bertelsmeier, Leadley, Thuiller, & Courchamp, 2012). The consequences of such forcing on local or regional species richness so far appear highly context dependent (e.g. Pauli et al., 2012; Vellend et al., 2017). In the future, however, the possible scale (Foster, Royer, & Lunt, 2017) and pace (Corlett & Westcott, 2013; Loarie et al., 2009) of climate change may go beyond what many species can tolerate (Urban, 2015), especially those adapted to cold conditions at high latitudes or elevations (e.g. Engler et al., 2011; McElwain, 2018). It has hence been speculated that climate change may increasingly rival land use as the dominant threat to biodiversity in the decades to come (Maxwell et al., 2016).

To evaluate the possible magnitude of future human impacts on biodiversity, and to guide societal responses, forecasting trajectories of biodiversity under different scenarios of future development has become an active field of research (Lurgi, Brook, Saltré, & Fordham, 2015; Pereira et al., 2010; Urban et al., 2016; Zurell et al., 2016). In this context, the predominant impact that land use has had on biodiversity so far is in salient contrast to the modest attention it receives by predictive biodiversity models. Recent reviews document that forecasts of how biodiversity may develop in the 21st century have concentrated on the effects of climate change but much less so on land‐use change, with this imbalance even increasing over time (Sirami et al., 2017; Titeux et al., 2016). Similarly, the combined and interactive effects of both drivers appear understudied (Forero‐Medina, Joppa, & Pimm, 2011; Pimm, 2008; Titeux et al., 2016). As a consequence, the relative impacts of land‐use and climate change on the unfolding biodiversity crisis are difficult to evaluate (de Chazal & Rounsevell, 2009; Pereira et al., 2010).

An important reason for the modest attention that land use receives in biodiversity forecasts is the scarcity of future land‐use scenarios (Titeux et al., 2016). A number of such scenarios have become available over the last one and a half decades (Busch, 2006; Hurtt et al., 2011; Rounsevell et al., 2006; Spangenberg et al., 2012; Verburg, Schulp, Witte, & Veldkamp, 2006), and some of these scenarios have also been used to predict how species richness of local assemblages may develop in the future (Kehoe et al., 2017; Newbold et al., 2015). However, the spatial and thematic resolution of these scenarios is usually considered inappropriate to represent habitat suitability for individual species (Barbet‐Massin, Thuiller, & Jiguet, 2012; Martin, Dyck, Dendoncker, & Titeux, 2013). The ecological requirements, or niches, of individual species are the basis of most models that predict climate effects on biodiversity such as species distribution models (SDMs, e.g. Araújo, Alagador, Cabeza, Nogués‐Bravo, & Thuiller, 2011; Thuiller et al., 2011), joint SDMs (e.g. Clark, Gelfand, Woodall, & Zhu, 2014; Maguire, Nieto‐Lugilde, Fitzpatrick, Williams, & Blois, 2015), hybrid SDMs (e.g. Dullinger, Gattringer, et al., 2012; Wessely et al., 2017) and most other types of dynamic range models (e.g. Fordham et al., 2018; Lurgi et al., 2015; Zurell et al., 2016). Linking climate and land‐use effects in such a type of modelling hence requires scenarios of future land use that deliver more fine‐grained spatial and thematic information.

To derive such scenarios, the complex interplay of land users (e.g. farmers) and the wider socio‐economic context in which they act needs to be taken into account. At landscape to regional scales, agent‐based modelling (ABM) represents a way towards appropriately considering these intricacies (Matthews, Gilbert, Roach, Polhill, & Gotts, 2007; Valbuena, Verburg, Bregt, & Ligtenberg, 2010). ABMs simulate human decision‐making, and with individual land owners/users as agents, they can simulate trajectories of usage for individual parcels of land at thematically fine resolutions (e.g. Gaube et al., 2009; Troost & Berger, 2014). Ideally, these simulations are based on understanding how farmers make decisions, including on the basis of anticipated strategies, adaptive behaviour and social interactions (An, 2012; Huber et al., 2018; Rounsevell & Reay, 2009). To develop such an understanding, participatory approaches are useful because they include stakeholders' perspectives in model calibration and thereby account for culture and traditions that often play an important role in land‐use decisions. Being closer to stakeholder perspectives, moreover, increases the policy relevance of such land‐use models (Bithell & Brasington, 2009; Kelly et al., 2013; Reeves & Zellner, 2010).

Although progress has been made in combining land‐use and biodiversity models (Kehoe et al., 2017; Newbold et al., 2015), coupling such individual‐ or farm‐scale ABMs with models like SDMs or their derivatives has hardly been attempted so far and could represent a powerful tool to integrate land‐use and climate effects in biodiversity forecasts, particularly on a regional scale. Here, we present such a model combination and apply it to predict changes in plant distribution across a Central European study region until the mid of the century. Apart from demonstrating the concept, we thereby compare the relative effects of climate and land‐use change scenarios on the future shrinkage or expansion of regional species ranges.

## 2 METHODS

### 2.1 Study region

**Figure 1:**
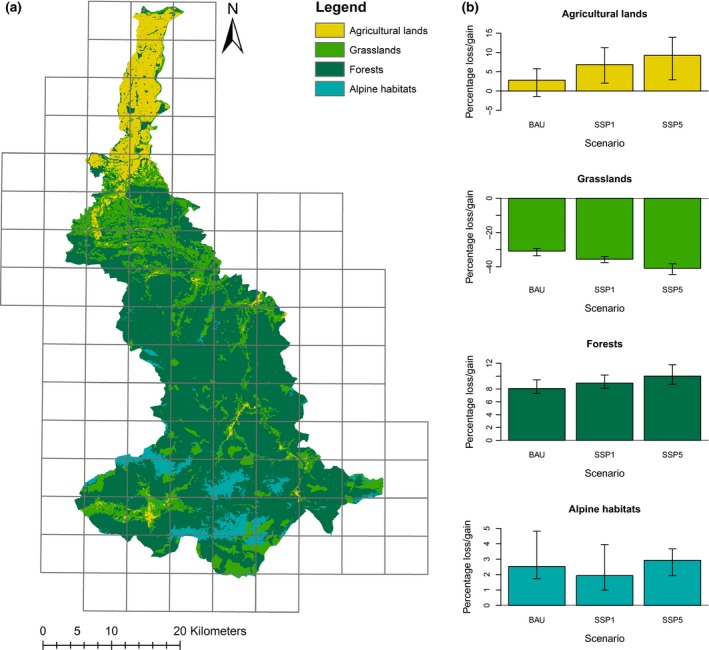
(a) Map of study area with current land use summarized into four habitat groups. The raster overlaying the map represents cells (each 3′ × 5′ in size) of the floristic mapping grid of Austria; (b) projected percentage changes of the area covered by these four habitat groups under different scenarios of land use (BAU, SSP1, SSP5) in comparison to their current extent. Barplots depict mean values for all five simulation runs for each scenario (combination), while error bars depict the minimum and maximum changes from all five simulation runs

The coupled model was developed for a subarea of the long‐term socio‐ecological research (LTSER) region ‘Eisenwurzen’ in Austria's Northern Limestone Alps. The area covers the upper part of the valley of the river Enns (Figure 1a). The region encompasses 18 municipalities, spread across 1,426 km^2^, is topographically highly diverse and includes a broad variety of land‐use systems. The southern parts are characterized by high mountains (highest elevation: 2,309 m), rugged topographic conditions, a cold climate (c. 5–6°C mean annual temperature), high annual precipitation (c. 1,200–1,800 mm) and a dominance of forests and livestock‐centred agriculture. Towards the north, elevation and precipitation decline (c. 800 mm), temperature rises (c. 9°C mean annual temperature) and arable lands gain importance. The largest city of the region, Steyr (elevation: 310 m a.s.l., c. 40,000 inhabitants), is situated in the northern part of the study area which is dominated by intensive cropland agriculture.

### 2.2 Data

#### 2.2.1 Species selection and data

We focused on all vascular plant species currently occurring in the study region and its immediate surroundings according to the floristic mapping of Austria (Niklfeld, 1998). The floristic mapping database holds complete species records for each cell of a 3′ × 5′ raster covering the entire country. Figure 1a shows the cells considered for compiling our species list.

For these species, we collated plot‐scale information on their current distribution from different existing databases (Office of the State of Upper Austria, 1993–2013; Pascher et al., 2011; Willner, Berg, & Heiselmayer, 2012). We only included plots that provided complete lists of vascular plant species, that is, where observers had intended to record all species present. We used plot data from the entire territory of Austria to get a better representation of the environmental niches of species. We supplemented the collated plots by 155 own ones that we recorded within the study region and its immediate surroundings to improve representation of under‐sampled land‐use types in the data set.

The final data set included 12,498 plots spread across Austria (Figure S1; Table S1). Accuracy of the geographical location of each plot varied between <10 and c. 250 m. Plot sizes varied between 2 and c. 2,000 m^2^. In case of the data of the Biotope Mapping Upper Austria (Office of the State of Upper Austria, 1993–2013), plots, mostly between 10 and 1,000 m^2^ in size, characterize polygons of the biotope map which themselves vary in size between 50 and 100,000 m^2^. The exact location of the recorded plots within these polygons is unknown. We thus assigned the location of the plots (i.e. their geographical coordinates) from this source to the centre of gravity of ‘their’ polygon.

We cross‐checked all vascular plant taxa names listed for the 12,498 plots and applied a consistent taxonomy following Fischer, Oswald, and Adler (2008). Crop species were excluded. Taxonomically critical species or subspecies were combined to aggregates or species (e.g. *Achillea millefolium* agg., *Aconitum variegatum* agg., cf. Table S2). From the remaining taxa, we further excluded all those occurring in <50 of the 12,498 records because model accuracy may significantly decrease with fewer observations (e.g. Wisz et al., 2008). The final set for modelling included 834 vascular plant taxa which we call species for convenience henceforth (Table S2).

#### 2.2.2 Current and future land‐use data

Land‐use information was assigned to the 12,498 plots in different ways (cf. Table S1). For a subset of 5,897 plots, land use had been directly classified in the field at the time of recording. This information was harmonized into 22 land‐use classes that combine information about type of usage and, for grasslands and arable lands, intensity of usage (Table 1). The remaining plots were taken from the Austrian Vegetation Database (Willner et al., 2012) which provides an assignment of each plot to alliances of the European phytosociological system of plant communities that also underlies standard European habitat typologies (Rodwell et al., 2002). Based on this assignment, we could cross‐tabulate each plot to one of our 22 land‐use classes.

**Table 1:** The 22 land‐use classes distinguished, the four broad habitat groups they have been assigned to, and the percentage area of each land‐use class under current conditions (CURRENT) or predicted (by the respective centroid simulation runs) under the three land‐use scenarios BAU (business‐as‐usual), SSP1 (sustainability‐oriented) and SSP5 (unconstrained economic growth)

Habitat group	Land‐use class	CURRENT	BAU	SSP1	SSP5
Agricultural lands	Arable land fallow and low input	0.41	0.51	0.59	0.6
Agricultural lands	Cereal crop	2.46	0.97	0.77	1.23
Agricultural lands	Cereal crop low input	0.21	0.78	0.42	0.26
Agricultural lands	Energy crop	0.01	0.7	2.43	0.69
Agricultural lands	Misc. arable land	0.79	0.83	0.82	0.82
Agricultural lands	Non‐cereal crop	2.21	1.62	0.35	3.1
Agricultural lands	Non‐cereal crop low input	0.19	1.36	1.35	0.33
Agricultural lands	Ruderal	2.53	2.53	2.53	2.53
Grasslands	Dry grassland	0.03	0.03	0.03	0.03
Grasslands	Extensive meadow (one‐ or two‐cut per year)	2.33	3.19	3.35	1.91
Grasslands	Extensive pasture (max. 1.5 livestock units per hectare)	6.67	7.4	7.23	5.07
Grasslands	Intensive meadow (min. three‐cut per year)	3.96	0.51	0.34	1.83
Grasslands	Intensive pasture (min. 1.5 livestock units per hectare)	4.6	0.45	0.25	0.94
Grasslands	Orchard meadow and fruit plantation	0.02	0.02	0.02	0.02
Grasslands	Riparian	1.02	1.02	1.02	1.02
Grasslands	Wetland	0.1	0.1	0.1	0.98
Forests	Broad‐leaved forest	23.6	22.31	27.85	33.22
Forests	Conifer forest	41.7	49.24	44.24	38.55
Forests	Felling area	1.28	0.38	0.35	1.64
Alpine habitats	Alpine grassland	1.02	1.02	1.02	1.02
Alpine habitats	Rock and scree	3.51	3.51	3.51	3.51
Alpine habitats	Scrub & Shrub (incl. krummholz)	1.39	1.5	1.45	1.58

A map of current land use in the study region was compiled by using information from the Integrated Administration and Control System (IACS). IACS provides information on land‐use type and intensity (number of cuts for meadows, average livestock density per farm and whether the farmer receives subsidies for organic farming in case of croplands) for each parcel of agricultural land (croplands, pastures, meadows, orchards) in Austria for the year 2014. It additionally provides data on the farm holds that use these parcels (e.g. farm size, farm type). We used these IACS data to define the land‐use intensity classes that we distinguished (Table 1). For meadows, we defined those as low intensity which are mown once or twice a year as has been common practice for centuries until the second half of the 20th century (Ellenberg, 2009). In case of pastures, we set the threshold of low versus high intensity to 1.5 livestock units per hectare following results that have shown that least impact of dairy farming occurs at stocking density of 1–2 livestock units/ha (Guerci et al., 2013). For croplands, the only information we could base intensity classification on was whether the farm follows standards of organic production which actually has a significant effect on the diversity of many taxonomic groups in agricultural land (Tuck et al., 2014). We acknowledge that variation in other features of land‐use practices such as crop selection (Seifert, Leuschner, & Culmsee, 2015) or time of mowing (Dengler, Janišová, Török, & Wellstein, 2014) is also important for biodiversity. However, as systematic information on these features was not available for the study region, it could not be considered in our models.

Outside of agricultural areas, land‐use information was complemented by a fine‐scale (10 × 10 m) raster map of EUNIS land cover types for Austria (Umweltbundesamt, 2014). Land‐use and land cover information from these sources was cross‐tabulated to the same 22 classes as used for the vegetation plots (Table 1). The resulting map was finally resampled to a 25 m raster using a majority rule in the case that several land‐use categories were overlapping one cell.

Corresponding maps of future land use (year 2050) at the same grain size of 25 m were modelled by calculating trajectories of future land‐use change according to three different scenario projections from the shared socioeconomic pathways (SSPs) family (O'Neill et al., 2014, 2017): a scenario describing a world of sustainability‐oriented growth and equality (SSP1), another one describing a world of rapid and unconstrained growth in economic output and energy use (SSP5) and a business as usual scenario (BAU). Based on the narratives of the scenarios and information from regional applications of the SSPs (Absar & Preston, 2015; Popp et al., 2017; Steininger et al., 2015), we made different assumptions on the development of yields, prices, subsidies, income, workload and forest development for each of these scenarios (see Table S3). These assumptions set the socio‐economic background for human decision‐making simulated with the ABM.

The ABM included the 1,329 agents (farm owners, two national parks) active in the region in the year 2014. We assigned each agent to the particular areas of land he or she managed at the time of initialization (2014) according to the IACS database. Based on a quantitative analysis of IACS farm data, we moreover assigned each farm to one of three farming types (either cash crop, processing or livestock) and one of five intensity levels (low‐input, three intermediate levels defined by the Austrian Agri‐Environmental Programme, intense production). Finally, we also classified each agent by a farming style. Farming styles represent different value systems held by land users (Schmitzberger et al., 2005). Based on 29 semi‐structured qualitative interviews with farmers and six additional ones with regional experts, each lasting up to 4 hr, we identified and refined five farming styles relevant for the study region: traditionalist, yield optimizer, support optimizer, idealist, innovative. We randomly assigned one of these styles to each farm at the beginning of each simulation run.

Agents are assumed to act goal oriented and are parameterized based on expected utility theory including stochastic modifications (Groeneveld et al., 2017). The farmers invest labour to generate economic income from their agricultural lands. Their main goal is a satisfactory balance between income and workload (= invested time). Each year, they review whether labour input and income meet certain thresholds: workload should not exceed 1,800 hr/year (average annual workload in Austria) and income should in any case exceed 20,000 €/year, and, in addition, the average income of all farmers of the same type in the region (i.e. farmers compare themselves to their peers). The outcome of this evaluation (i.e. whether one or both thresholds are met) determines whether an agent decides for one of 10 different actions (such as switch from low to high intensity cropland or grassland as defined above, or vice versa, switch to different land‐use types, termination etc., see Table S4 for a full list of options) for the subsequent year. Apart from the evaluation outcome, the probability of taking a particular decision depends on farming type and style (e.g. an idealist and a yield optimizer tend to respond differently to the same combination of income and workload). We assigned probabilities for particular decisions to the different types of agents based on information derived from the 35 qualitative interviews. Patches of land that are abandoned (due to termination or area reduction of a farm) become part of the rental market where they are available to farms that take the decision to expand (cf. Beckers et al., 2018). Agricultural areas that are not cultivated underlie a succession process, making them unsuitable for agricultural activity after 5 years. The consequences, in terms of labour and income, of the action taken in a specific year are the basis of the evaluation in the next year. These consequences are calculated based on exogenous socio‐economic input variables such as standard gross margins per agricultural activity, yields of croplands and grasslands, market prices for agricultural goods (crops and livestock products) and subsidies (area related). These exogenous parameters are predefined for each year, and vary among the scenarios. Eventually, results of each individual farmer's decisions are translated into annual updates of the land cover map. Further explanation of the ABM, its design and its parameterization can be found in the Appendix (Figure S2; Tables S3 and S4).

As decisions of land owners are not modelled in a deterministic, but in a probabilistic way (i.e. options are randomly selected according to the assigned probabilities as described above), we repeated simulations 100 times per land‐use scenario. From the resulting 300 land‐use maps for the year 2050, we selected five per scenario, that is, a total of 15, for further modelling of biodiversity response. As these five simulations should span the full breadth of possible land‐use change under the respective scenario, we applied the following selection procedure: for each simulated future land‐use map, two indices were calculated: evenness, that is, homogeneity of land‐use classes in terms of the area they cover, and the total area of intensively used land. Intensively used land was defined as cropland belonging to farms that do not receive subsidies for organic practices, pastures with more than 1.5 livestock units/ha and meadows with more than two cuts per year. For each scenario, the values of these two indices resulting from each of the 100 simulation runs were plotted against each other. We then selected the five simulations closest to the centroid and the four corners of a minimum bounding rectangle across the index results from all 100 simulations.

#### 2.2.3 Current and future climate data

Maps of current climatic conditions were derived from combining Worldclim data for temperature with a high‐resolution data set for precipitation of the European Alps (Isotta et al., 2014) providing average (1970–2005) monthly precipitation sums at a resolution of 5 km. The precipitation data were downscaled to a resolution of 100 m using ordinary kriging with elevation as co‐variable. Worldclim monthly temperature variables (mean, minimal and maximal monthly temperature) were downscaled as in Dullinger, Gattringer, et al. (2012). As the Worldclim data represent average values from 1950 to 2000, these were corrected using the E‐OBS climate grids available online (https://www.ecad.eu/download/ensembles/download.php) to average values ranging from 1970 to 2005 to equal the reference period of the precipitation data.

Future climate was characterized by three different IPCC5 scenarios from the Representative Concentration Pathways family (Moss et al., 2010): RCP2.6 (‘mild’ climate scenario), RCP4.5 (‘intermediate’ climate scenario) and RCP8.5 (‘severe’ climate scenario). We therefore downloaded climatic models which were generated by Météo‐France/Centre National de Recherches Météorologiques using the CNRM‐ALADIN53 model, fed by output from the global circulation model CNRM‐CM5, available at the Cordex portal (http://www.euro-cordex.net/) for a 35 year period around 2050. These monthly time series were statistically downscaled from the original 11′ resolution by (a) calculating differences (‘deltas’) in monthly temperature and precipitation values between hindcasted historical (mean 1970–2005) and forecasted future climatic parameters (mean 2033–2067) at the original spatial resolution; (b) spatially interpolating these differences to a resolution of 100 × 100 m using cubic splines; and (c) adding these differences to the downscaled current climate data of the same climatic variables (Dullinger, Gattringer, et al., 2012; Zimmermann et al., 2009). Subsequently, we used these spatially refined temperature and precipitation grids to derive maps of four bioclimatic variables which, in combination, represent temperature and precipitation conditions together with their seasonal variability, and which are known to influence species distributions (Root et al., 2003): (a) minimum temperature of the coldest month (BIO6), (b) temperature annual range (BIO7), (c) precipitation seasonality (BIO15), (d) precipitation of the warmest quarter (BIO18). Correlations (Pearson's *r*) among these variables were <.75 throughout.

#### 2.2.4 Other environmental data

We used two additional variables to better characterize the environmental niche of the species: solar radiation and percentage of calcareous bedrock substrate. In temperate regions of the northern hemisphere, solar radiation income mainly distinguishes topographically warmer sites facing southern directions from cooler ones facing north. We calculated direct and diffuse solar radiation income (in kWh/m^2^) using the Potential Incoming Solar Radiation Tool of the System for Automated Geoscientific Analyses (version 2.2.0, http://www.saga-gis.org). As input data we used a Digital Model of the European Environmental Agency (https://www.eea.europa.eu/data-and-maps/data/eu-dem) with a cell size of 100 m. Calculations were done for the days 20 March and 21 June on an hourly basis, and resulting radiation income values finally summed.

Calcareous substrate is important because many species of the central European flora are known to be sensitive to soil chemistry and especially to the contrast between siliceous and calcareous soils (Ellenberg, 2009). We therefore derived the presence and absence of calcareous bedrock in each 100 × 100 m cell of the study area from a 1:200,000 geological map of Austria (cf. Dullinger, Willner, et al., 2012).

### 2.3 Species distribution models

#### 2.3.1 Model calibration and evaluation

We modelled the realized niche of each species by combining presence/absence at the 12,498 vegetation plots with information on current land use, current climate, solar radiation income and presence/absence of calcareous substrates at the sampling sites. We applied three statistical modelling techniques available in the biomod2 (Thuiller, Lafourcade, Engler, & Araújo, 2009) library in R (R Development Core Team, 2014): random forests (RF), artificial neural networks (ANN) and gradient boosting machines (GBM). We selected these techniques because they are relatively insensitive to a low ratio of the number of occurrences to the number of predictors (land use with 22 categories + 4 climatic variables + 2 additional environmental variables in our case, Cutler et al., 2007; Dasgupta, Sun, König, Bailey‐Wilson, & Malley, 2011). All models were run with the default settings in biomod2.

We evaluated the models by randomly dividing the original data set into two parts, one for calibrating models (80%) and one for evaluating them (20%) by means of the true skill statistic (TSS, Allouche, Tsoar, & Kadmon, 2006). This process was repeated three times to make sure that the estimated predictive accuracy was not influenced by the random partitioning. To assess the importance of predictor variables in the fitted models, we used a built‐in permutation function that scores the impact the respective variable has on the discrimination ability of the model on a scale ranging from 0 to 1 (Thuiller et al., 2009).

#### 2.3.2 Model projections

Calibrated models were used to project the occurrence probability of all species across the study region under current and possible future (= year 2050) land use and climatic conditions by means of an ensemble forecast approach (Araújo & New, 2007). The contribution of each of the three models to the ensemble forecast of each species was weighted according to its TSS score. Models with a TSS score <0.5 were excluded from contributing to projections. To match the resolution of the land‐use data, projections were done onto a 25 m raster of the study region, with constant climate, solar radiation and substrate across all cells within a 100 × 100 m cell of these coarser grids of these variables. Solar radiation and calcareous substrates were assumed to remain constant in projections for 2050. The probabilistic ensemble forecasts were translated into binary maps using the value that maximizes the TSS score as the threshold for distinguishing presence and absence predictions.

### 2.4 Analysis of projections

To assess the effects of the different land‐use and climate scenarios on the distribution of the 834 species, we computed the projected range size change (= number of 25 × 25 m cells predicted to be suitable in the future/number of 25 × 25 m cells predicted to be suitable under current land‐use and climatic conditions) and the so‐called exposure (Choe et al., 2017). Exposure is calculated as (C − O)/C, where C is the currently suitable area and O is the overlap between the area suitable under both current and under future conditions. Hence, the first index measures the change in range size and the second one the spatial displacement of ranges.

To evaluate whether results differ for subsets of species specifically adapted to particular habitats, we defined four broad habitat categories: forests, agricultural lands, grasslands and alpine habitats. All 22 land‐use classes were assigned to these categories (see Table 1). Species which had at least 75% of their occurrences in vegetation plots assigned to one category were defined as specialists of the respective habitat. For the species in each of these four groups, we then calculated a log response ratio as the natural logarithm of projected range size change (as defined above) to make the possible values of suitable area expansion and shrinkage, respectively, symmetric around zero. We repeated computations after defining habitat specialists by lower (50% of occurrences) and higher (90%) thresholds, but results were essentially the same and are hence not reported.

To compare the magnitude of effects of land‐use and climate scenarios on changes of potential range size of species or exposure, we fitted Generalized Linear Mixed Effects models, with either the natural logarithm of range size change or exposure as response, land‐use and climate scenarios (including the baseline, i.e. current land use and climate) and their interaction as fixed effects, and a random intercept term for species identity. We assumed a normal distribution of the response in case of the logarithm of the range size change and a binomial distribution (with a logit link function) in case of exposure. We partitioned the variation explained by climate change scenarios, land‐use scenarios and their interaction by recalculating *R* ^2^‐values for models that had either one or the other of these two predictors (land use or climate) omitted, or the interaction term replaced by an additive combination. *R* ^2^‐values were calculated by means of the ‘r.squaredGLMM’ function in the ‘MuMIn’ package of R (Barton, 2019).

Finally, we explored the spatial change of potential (local) species richness resulting from the (potential) range shifts of the individual species. We therefore overlaid the projected distribution maps of all species for each scenario and computed changes in the number of species per 25 m cell as compared to current conditions.

## 3 RESULTS

### 3.1 Trends in land use and climate within the study region

Summarized into broad categories, the study region is currently covered by 67% deciduous and conifer forests (including felling area), 19% grasslands, 9% agricultural lands and 6% alpine areas (see Figure 1a). The ABMs simulated low to moderate changes to these percentages until the year 2050, with little differences among scenarios. In general, forests are predicted to increase at the expense of grasslands, while the size of agricultural lands slightly increases (Figure 1b). At the level of the 22 land‐use classes, trends differ more strongly among land‐use change scenarios (Table 1).

Predicted regional temperature increase until 2050 is relatively pronounced, with changes in the means of the coldest month (as compared to the reference period 1970–2005) of +2.4°C, +3.0°C and +3.8°C for RCP2.6, RCP4.5 and RCP8.5 respectively (Table S5). Only slight changes, which differ among scenarios, are predicted for annual temperature ranges and precipitation‐related variables.

### 3.2 Species distribution models

The discrimination ability of SDMs was high to very high in general. TSS values of the Ensemble Models ranged from 0.647 to 0.995 and ROC values from 0.894 to 0.999 (see Table S2 for full information on model performance). For two of the 834 species, we could not fit any single model with a TSS > 0.5, and consequently, they were omitted from further analysis.

### 3.3 Variable importance

**Figure 2:**
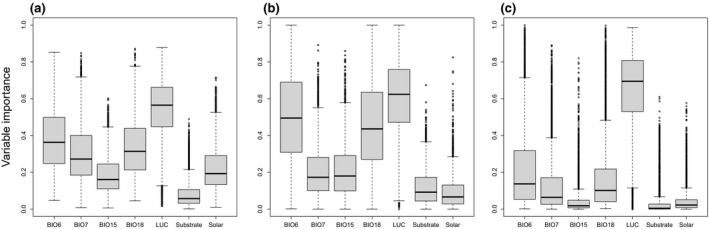
Importance of predictor variables in models, evaluated separately for the three modelling techniques: (a) random forests, (b) artificial neuronal networks and (c) gradient boosting machine. Each boxplot represents results for 832 species. Black lines within the boxes mark the median, box boundaries the upper and lower quartiles and whiskers the 10th and 90th percentiles. The variables modelled and tested are: BIO6 (Min Temperature of the Coldest Month), BIO7 (Temperature Annual Range), BIO15 (Precipitation Seasonality), BIO18 (Precipitation of Warmest Quarter), LUC (22 land‐use classes), Substrate (presence/absence of calcareous bedrocks) and Solar (solar radiation income kWh/m^2^)

Importance of the seven predictor variables in the SDMs varied strongly among species, but also between modelling techniques (RF, ANN, GBM, see Figure 2). On average across species, however, land use was the most important of all single predictor variables included, independent of the modelling technique. The predominance of land use was particularly clear with GBMs, and somewhat less pronounced with RF and ANN. The median importance score of land use was relatively constant (c. 0.6–0.7) across the three modelling techniques (see Figure 2).

From the four bioclimatic variables used, minimum temperature of the coldest month (BIO6) was consistently the most important one in RF, ANN and GBM. However, its numerical importance value varied considerably among modelling techniques (c. 0.1–0.5). The other bioclimatic variables showed similar variation around a lower level of the median. Solar radiation and substrate were, on average across species, the least important predictors in the SDMs (see Figure 2).

### 3.4 Projected changes in potential range size and exposure

#### 3.4.1 Under current climate but varying land‐use scenarios

**Figure 3:**
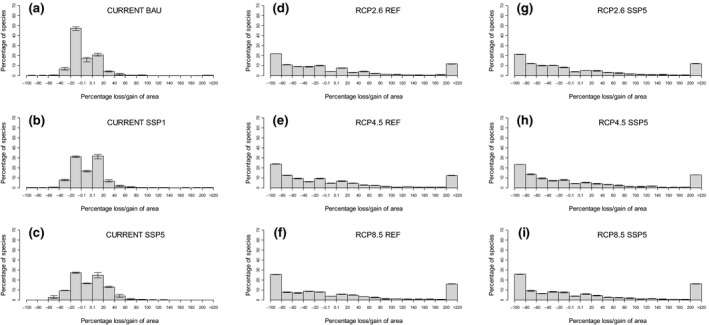
Projected changes in the size of suitable ranges of 832 species in the study region. Changes are depicted for different combinations of land‐use and climate change scenarios: (a–c) proportional loss/gain of suitable area under current climate (‘CURRENT’) but varying land‐use scenarios, (d–f) under varying climate but current land use (‘REF’), and (g–i) under varying climate and the SSP5 land‐use scenario. Barplots depict mean values of five simulation runs for each scenario, while error bars depict the minimum and maximum changes from these five simulation runs

If neither the climate nor the socio‐economic background conditions would change until 2050 (current climate + BAU land‐use scenario), SDMs forecast that a majority of species will lose moderate fractions of their currently suitable ranges (Figure 3a). In particular, c. 20% of the species are predicted to expand their ranges by up to 20%, 50% of the species face shrinkage of ranges by up to 20% and c. 17% of species will not experience measurable changes to the size of their suitable ranges. More pronounced range losses or gains are rare. Similarly, spatial displacement of suitable ranges is moderate (Figure S3a). For c. 90% of the species, >60% of the currently suitable range remains suitable in the future (i.e. exposure index <0.4).

In both the sustainability‐oriented (SSP1) and unconstrained growth (SSP5) scenarios, proportions of winners and losers are approximately balanced, but changes are slightly more pronounced in SSP5. While c. 85% of the species do not change their range sizes by more than 20% in SSP1 (Figure 3b), shrinkage or expansion of up to 60% is predicted for c. 30% of the species in SSP5 (Figure 3c). The results are similar by trend for exposure, with spatial displacement rates slightly higher in the case of SSP5 (Figure S3b‐c).

#### 3.4.2 Under varying climate but current land use

Predictions are vastly different if, by contrast, land use is kept constant (= at current conditions) but climate changes (Figure 3d–f; Figure S3d–f). Under RCP2.6 (Figure 3d), there are many more losers (c. 60%) than winners (c. 35%), and both losers and winners vary pronouncedly in the magnitude of range loss or gain. In particular, more than 20% of the species are predicted to even lose 80%–100% of their current ranges while c. 10% will expand their ranges by more than 200%. Predictions are similar, in principle, for the stronger climate scenarios, but both extreme ‘losers’ and ‘winners’ tend to become more frequent the more severe the climatic scenario (Figure 3d–f).

Again, results for exposure are similar (Figure S3d–f). Climate change effects are much stronger than those of land‐use change. Under RCP 2.6, approximately 20% of the species experience a more or less complete spatial displacement (>90%) of suitable areas. This percentage increases to about 25% under RCP 8.5. By contrast, c. 20% of the species retain >90% of their suitable areas in the future across all scenarios. Exposure values in between 0.1 and 0.9 are more or less uniformly distributed across the remaining 50%–60% of the species.

#### 3.4.3 Under varying climate and SSP5 land use

When varying both climate and land use in combination, predictions for potential range size change closely resemble those achieved under constant (current) land use and changing climate (Figure 3g–i). However, for exposure, the combination of climate and land‐use change results in even stronger displacement than under changing climate but constant land use (Figure S3g–i). In particular, under SSP5, the number of species that retain more than 90% of their currently suitable area shrinks to 5%–8%, depending on the climate change scenario, while all other exposure values increase at approximately the same rate (as compared to a combination of changing climate and constant land use). Very high displacement values (>90%) are, again, most frequent under the most pronounced RCP8.5 scenario.

In summary, these predictions indicate that future climate change will have a much stronger effect on regional plant distribution than land‐use change. These descriptive results are corroborated by partitioning the variance in range change and exposure explained by the GLMR among the land‐use and climate scenarios (Table 2; Table S6). As evidenced by conditional *R* ^2^‐values, the big part of the variation in range change, and to a lesser degree also of exposure, is due to idiosyncratic response of species to the changing conditions. The marginal *R* ^2^‐values of fixed effects, however, are much higher for climate than for land‐use scenarios, in case of both range size change and exposure. Interactions among climate and land‐use scenarios had negligible effects on both indicators.

**Table 2:** Results of a linear mixed‐effects model relating the natural logarithm of the ratio of the number of cells predicted to be suitable to the 832 model species in the future and under current conditions, respectively, to climate change scenario, land‐use change scenario and their interaction. Lower AIC (Akaike information criterion) values indicate better models. $R_{m}^{2}$ and $R_{c}^{2}$ are the marginal and conditional *R* ^2^‐values of the model

Predictors	Estimate	*SE*	*p*‐value	AIC	$R_{m}^{2}$	$R_{c}^{2}$
Climate change scenario × Land‐use change scenario				257,592	0.018	0.668
RCP2.6	−0.548	0.036	<.001			
RCP4.5	−0.644	0.036	<.001			
RCP8.5	−1.044	0.036	<.001			
BAU	−0.018	0.036	.613			
SSP1	−0.002	0.036	.945			
SSP5	0.008	0.036	.823			
RCP2.6:BAU	−0.024	0.050	.631			
RCP4.5:BAU	−0.022	0.050	.660			
RCP8.5:BAU	−0.046	0.050	.357			
RCP2.6:SSP1	0.002	0.050	.971			
RCP4.5:SSP1	0.001	0.050	.985			
RCP8.5:SSP1	−0.020	0.050	.698			
RCP2.6:SSP5	−0.004	0.050	.930			
RCP4.5:SSP5	−0.001	0.050	.989			
RCP8.5:SSP5	−0.020	0.050	.687			
Excluding						
Climate change scenario				261,041	<0.001	0.650
Land‐use change scenario				257,516	0.018	0.668
Climate change scenario:Land‐use change scenario				257,534	0.018	0.668

### 3.5 Loss/gain of suitable area for subsets of species

**Figure 4:**
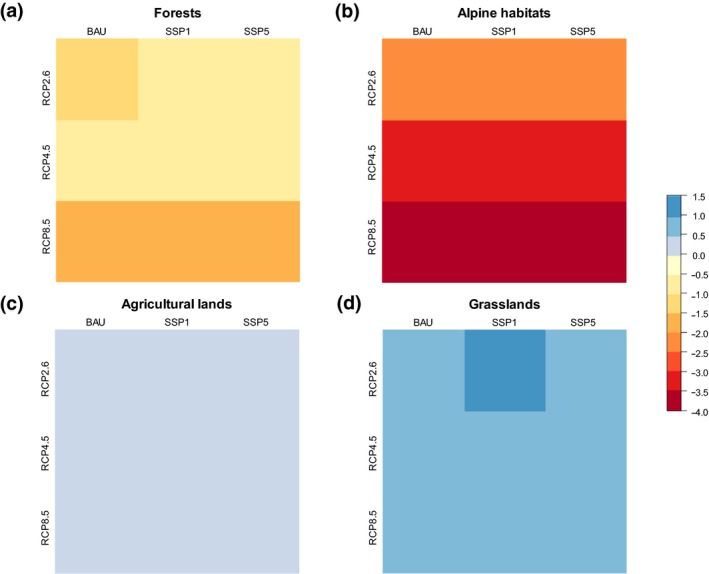
The amount of range change calculated separately for species specialized to (a) forests, (b) alpine habitats, (c) agricultural lands, (d) grasslands. Changes are depicted for the land‐use and climate change scenario combinations indicated along the *x*‐ and *y*‐axes and have been calculated as the natural logarithm of the ratio of the number of cells predicted to be suitable in the future and under current conditions respectively. Colours represent average change values across all species belonging to the respective habitats (forests: 160 species, alpine habitats: 147 species, agricultural lands: 57 species, grasslands: 165 species)

Among habitat specialists, alpine species are those likely suffering most under the predicted changes. The amount of range they are predicted to lose depends on the climatic, but not on the land‐use scenario (Figure 4b). Forest species rank second among losers (Figure 4a). Similar to alpine species, range loss of forest species tends to be more pronounced under strong climate change, with some additional effect of the business‐as‐usual land‐use scenario under RCP2.6. Specialists of agricultural lands and grasslands both show moderate increases in range size under all land‐use and climatic scenarios (Figure 4c,d).

### 3.6 Spatial distribution of loss and gain

**Figure 5:**
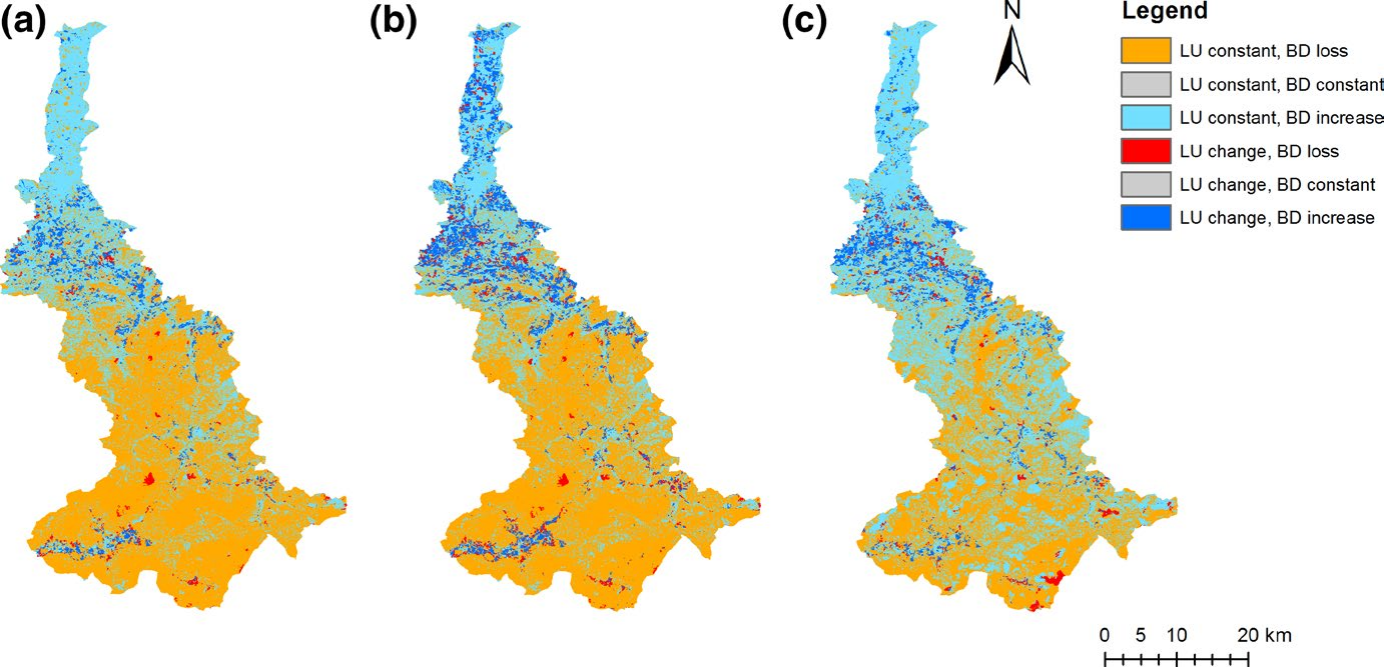
Maps of the study area combining information on changes in land use (LU) and potential species richness (BD) for the three most likely combinations of land‐use and climate change scenarios: (a) SSP1 and RCP2.6, (b) BAU and RCP4.5, (c) SSP5 and RCP8.5. Changes were calculated in comparison to current land use and climate and associated species richness. Land‐use change is defined as change from one habitat group (agricultural lands, grasslands, forests and alpine habitats) to another

The change in the distribution of (local) potential species richness across the landscape reflects the range size changes of different specialist groups. Potential richness increases in those parts of the region which are dominated by arable lands and grasslands, mainly in the north, while it decreases where forests or alpine habitats prevail, that is, in the south (Figure 5). The maps also demonstrate that land‐use change has a relatively greater effect of species gains in the north than on species losses in the south (darker blue areas are more prominent than darker red areas in Figure 5). Interestingly, the area with a rise in potential species number is larger and expands further to the south under RCP8.5 than under the less pronounced climate scenarios.

## 4 DISCUSSION

Taken together, the key findings of our study are, first, that both climate and land use determine the realized niches of the studied plants, with variation in type and intensity of land use having a stronger effect than any single climatic variable in the model. And second, that in contrast to the strong effect that land use has on the current distribution of species, the future distribution of suitable ranges in our study region appears much more responsive to climate than to land‐use scenarios. The apparent contradiction arises from the weak effect that alternative future socio‐economic backgrounds have on future land use, whereas the regionally pronounced temperature increase has a marked impact on the study area's climate.

### 4.1 Land‐use change effects

While expert assessments consistently emphasize the overwhelming effect of human land use on species threat (Baillie, Hilton‐Taylor, & Stuart, 2004; Maxwell et al., 2016), the land‐use signal is often relatively weak in SDMs (e.g. Barbet‐Massin et al., 2012; Riordan & Rundel, 2014; Thuiller, Araújo, & Lavorel, 2004). This apparent contradiction is usually attributed to a lack of both spatial and thematic resolution of available land‐use information (e.g. Austin & Van Niel, 2011; Martin et al., 2013; Pearson, Dawson, & Liu, 2004). Indeed, the land‐use information underlying many SDM applications is derived from remotely sensed land cover maps that often have spatial resolutions too coarse for matching them with plot‐scale species distribution data (e.g. Keil et al., 2012; Luoto, Virkkala, & Heikkinen, 2007). Moreover, these maps do not reflect important distinctions among land management practices (e.g. grazing vs. mowing: Schläpfer, Zoller, & Körner, 1998) and intensities of usage (Erb et al., 2013; Laliberté et al., 2010; Newbold et al., 2015). Our results corroborate this interpretation. Using a relatively detailed classification that accounts for both the type of usage and its intensity at the spatial scale of local species assemblages, we find that variation in land‐use type and intensity has a stronger effect on current plant species distribution in Austria (i.e. the region covered by our vegetation plots) than either temperature or precipitation gradients. This result is particularly remarkable because Austria is a mountainous country with strong climatic contrasts between the warm and dry eastern lowlands and the cold and humid high elevations of the Alps. The predominant effect of land use on species distributions reflects pre‐adaptation of plant species to particular types of habitats, such as forests or grasslands, and the associated differences in resource availability, ambient conditions and disturbance regimes (Grime, 2001). It is in line with the well‐known impact that a long history of human usage has had on the composition of plant communities across Europe (e.g. Ellenberg, 2009).

Despite the importance of land use in the SDMs, the impact of land‐use scenarios on potential future plant distribution in the study region is much weaker than the impact of climatic scenarios. The apparent contradiction is explained by the modest changes in land use predicted by the scenarios. This relative stability occurs although the number of active farms decreases considerably (by 36% in the BAU scenario, continuing a decrease of about the same magnitude over the 35 years preceding the year 2014), that is, the average farm size increases. It can be explained by a number of real constraints on land‐use development in the area. First, the region's topography, especially in the southern parts, represents terrain not easily accessible by machinery. As a result, establishing production systems other than the currently dominating ones, that is, permanent grassland‐based dairy farming and forestry, is difficult (Trnka et al., 2009). Second, there are legal constraints on land‐use development as the region overlaps with two national parks which take approximately 8% of the modelled area, and forests are protected in Austria from being converted into other forms of land use. As forests cover c. 65% of the area, this restriction is particularly important in the study region and precludes loss of forest species as a consequence of deforestation. Third, regionally specific traditions of farming systems additionally restrict the options perceived by farmers. As an example, types of land use that have no recent history in the area but could become attractive under a warming climate, such as winegrowing, did hardly play a role in decision‐making according to our interviews. Taken together, there are a number of environmental, legal and cultural constraints that restrict the options of farmers to cope with socio‐economic changes and to adapt to climate change impacts over the next decades. As a consequence, ABM simulations result in broadly similar development of land cover maps despite partly pronounced differences in the assumed future development of prices, subsidies, income and workload. It might, however, be that future climate change and/or societal changes will encourage or even force land owners to consider new farming strategies beyond what currently appears conceivable to them (Li, Juhász‐Horváth, Pintér, Rounsevell, & Harrison, 2018; Trnka et al., 2009). Coupling land‐use and climate scenarios such that they integrate possible climatic effects on yields, prices and workloads associated with currently grown or alternative crops and cultivars could be an appropriate way towards widening the perceived option space of agents.

### 4.2 Climate change effects

Our coupled model suggests a strong effect of future climate on potential regional plant ranges. These results corroborate other modelling studies in mountainous environments (e.g. Dirnböck, Dullinger, & Grabherr, 2003; Hämmerle et al., 2018; Randin et al., 2009). However, the strong climate change effect is not independent of land use but rather results from an interplay with regional topography and land‐use patterns. On the one hand, available area for plant colonization changes with elevation. Both in Austria as a whole (Essl et al., 2009) and in the study region, available area peaks at montane to subalpine elevations and strongly decreases above the treeline (c. 1,900 m a.s.l. in the study region). This topographical constraint is mainly responsible for the particular threat that the warming climate poses to alpine species, as found in other studies (Dullinger, Gattringer, et al., 2012; Engler et al., 2011). For forest species, climate‐driven shift of suitable ranges is restricted by both topography and land‐use patterns. Forest species show declining range sizes even though all land‐use scenarios predict an increase of forest cover in the region. This apparent contradiction arises because simulated forest expansion mainly occurs at the expense of abandoned grasslands in montane areas. At the same time, climate warming shifts the ranges of many montane forest species upward to elevations where there is no forest anymore, either because they are above the current climatic treeline or because the rugged terrain (widespread rock faces, debris cones and avalanche paths) does not allow for forest establishment or because the areas are used for summer farming and thus kept free of forest by pasturing and regular clearing. Consequently, these species cannot make full use of their expanding habitat because the local climate at the ‘new’ forest sites is not suitable to them anymore, and the sites that become climatically suitable are free of forests. In the long run, this contradiction may attenuate with the rise of the climatic treeline. However, this process is likely slow (e.g. Dullinger, Dirnböck, & Grabherr, 2004), and constraints from topography and land use will strongly limit forest expansion at and above the treeline over the next centuries in the European Alps (Holtmeier & Broll, 2005; Tasser, Leitinger, & Tappeiner, 2017).

At the other end of the elevational gradient, species that are currently restricted to the lowlands are among the potential winners of climate change. The number of such winners would have further increased, if we had included species from warmer areas of Austria that do not grow within or in the surroundings of the study area today. Range increases of warmth‐adapted species likely also drive the increase in potential local species richness at many forest sites under strong climate change despite range losses of forest specialists. There are obviously a number of generalist species from (currently) lower and warmer elevations that can grow in forests and could hence colonize the mountain forests in the south of the study region when warming is substantial. In addition, species of montane grasslands will find climatically suitable areas in current alpine grasslands thus potentially increasing species numbers at high elevations despite the loss of alpine specialists. However, species bound to particular types of land use may not be able to realize their climatic potential under climate change unless land‐use decisions have adapted to the upward shift of climatic conditions. Specialists of agricultural lands, for example, the land‐use type most widespread in the warmest part of the study area, show no or only moderate expansion of suitable area in our simulations, mainly because ABMs predict agricultural production to remain more or less where it is today.

### 4.3 Do we need land‐use scenarios for predicting future species distributions?

The simulated response of species to climate versus land‐use scenarios might be interpreted in the sense that focusing on climate instead of land use in biodiversity forecasts is actually justified. However, there are a number of caveats. First, a considerable part of the biodiversity in the study region, and in cultural landscapes of Europe in general, is concentrated in small remnants of natural or semi‐natural habitats such as mires, dry and wet grasslands or rock outcrops which are out of use or managed for conservation purposes (Dengler et al., 2014; Ellenberg, 2009; Marini, Fontana, Scotton, & Klimek, 2008; Tscharntke, Klein, Kruess, Steffan‐Dewenter, & Thies, 2005). For these remnants we have assumed that current ‘no‐land‐use’ regimes remain unchanged, that is, that they are conserved as such. These assumptions are not unrealistic because at least part of these remnants is under legal protection. However, there will still be many cases where such patches fall victim to competing interests (Henle et al., 2008). Such changes have little effects on area statistics but potentially large ones on regional species pools (Haddad et al., 2015). Consequently, the future of biodiversity in many regions of Europe may be determined more by the fate of these small patches than by changes to the usage of the matrix (Wessely et al., 2017; Wintle et al., 2019). Further land‐use scenario development for biodiversity forecasts should hence particularly address the fate of these remnants. Second, the results achieved in our case study region could be representative for other cultural landscapes of Central and Western Europe, especially those at the margins of the Alps and other mountain regions. These landscapes do not only have a long tradition of usage, they have also undergone at least half a century of intensification including application of fertilizers, herbicides and insecticides, multiple mowing of grasslands, land consolidation or amelioration techniques (Benton, Vickery, & Wilson, 2003; Graf, Müller, Korner, Jenny, & Jenni, 2014; Poschlod, Bakker, & Kahmen, 2005). In combination with legal constraints, options for further land‐use intensification (apart from destruction of remaining semi‐natural remnants, see above) are thus relatively limited, and future alterations may rather result from the abandonment or reforestation of economically marginal parts of the land (Giupponi, Ramanzin, Sturaro, & Fuser, 2006; Henle et al., 2008). As a consequence, major changes to land‐use patterns and thus species distributions are unlikely unless legal or economical frameworks change more drastically than supposed in the SSP scenarios and our ABM assumptions. The situation is completely different, however, where regions still contain large tracts of natural areas that are of agricultural interest or where low‐intensity land‐use systems still prevail, and where, simultaneously, legal constraints on land‐use changes are weaker than in the study area. In these regions, future habitat transformations resulting from, for example, the clearing of forests (e.g. Wearn, Reuman, & Ewers, 2012), or the intensification of cropland use (Kehoe et al., 2017) will certainly have a drastic impact on the distribution of species and their diversity. This is particularly true in case of tropical regions where deforestation is the major issue for conservation (Klink & Machado, 2005; Rudel, Defries, Asner, & Laurance, 2009) and including land‐use change into modelling is hence key to realistic biodiversity scenarios.

For these reasons, we argue that including land‐use scenarios into biodiversity forecasts is generally important, because the relative effects of climate and land‐use change may vary considerably across regions. ABMs are particularly well‐suited tool in this respect, especially on landscape to regional scales, because, first, they can represent future land‐use patterns at a spatial and thematic resolution relevant to species distribution. Second, by explicitly integrating individual decision‐making based on rules derived from stakeholder interviews, they better capture the complex motivation that often drives plot‐level land‐use change (Janssen & Ostrom, 2006). As an example, modelling future land use in our area based exclusively on principles of yield or income optimization would have ignored important differences in farmers' attitudes that entered our model via the five farming styles. These attitudes modified the agents' responses to particular changes in socio‐economic conditions and, as discussed, generally restricted the ‘option space’ perceived by farmers in their cultural contexts. ABMs thus allow a realistic representation of the farmers' decision context which is crucial for assessments of policy instruments in terms of their implications for biodiversity. We hence suggest that the development and implementation of such scenarios via ABMs and their linkage with biodiversity models should be intensified. Moreover, ABMs can potentially be coupled with any type of biodiversity models, not only with SDMs that model potential species distributions. Links with dynamic range models (Lurgi et al., 2015; Zurell et al., 2016) that integrate the demography and dispersal of species and simulate real instead of potential range changes may be especially attractive as both types of models are able to represent transient change. They can therefore link the spatio‐temporal dynamics of species' populations with the spatio‐temporal dynamics of landscape patterns—and this may lead to important differences in predictions among species or taxonomic groups in dependence of their dispersal capacity (Fordham et al., 2018; Wessely et al., 2017). When coupling ABMs with such dynamic range models, it is moreover possible to include the feedbacks of changing biodiversity on farming practices. These feedbacks can be particularly important when changes to biodiversity affect ecosystem services relevant to farmers, such as crop pollination (Synes et al., 2019). The necessary effort to parameterize and run such combined socio‐ecological models is certainly larger, but its forecasts will also be more realistic than those of climate impact models in isolation (Evans et al., 2013; Fordham et al., 2018).

## Data Availability

Vegetation plot data from Willner et al. (2012) are part of the European Vegetation Archive (http://euroveg.org/eva-database) and are available from there, or directly from the authors, on request. Vegetation plot data recorded from this study are also available from the authors on request. Vegetation plot data from Pascher et al. (2011) and Office of the State of Upper Austria (1993–2013) were used under license for this study. These data are available from kathrin.pascher@boku.ac.at and from guenter.dorninger@ooe.gv.at, respectively, with the permission of the owners. Access to regional farm data (IACS Austria) was provided by the Austrian Ministry of Sustainability and Tourism under license for this study.
