# Supplementary material for: A socio‐ecological model for predicting impacts of land‐use and climate change on regional plant diversity in the Austrian Alps
Source: Glob Chang Biol. 2020 Jan 29;26(4):2336–52. doi: 10.1111/gcb.14977 (PMC7155135; doi:10.1111/gcb.14977)
Supplement: Supplementary file 1 [file GCB-26-2336-s001.docx]

**Supporting Information**

**A socio-ecological model for predicting impacts of land-use and climate change on regional plant diversity**

Iwona Dullinger, Andreas Gattringer, Johannes Wessely, Dietmar Moser, Christoph Plutzar, Wolfgang Willner, Claudine Egger, Veronika Gaube, Helmut Haberl, Andreas Mayer, Andreas Bohner, Christian Gilli, Kathrin Pascher, Franz Essl and Stefan Dullinger


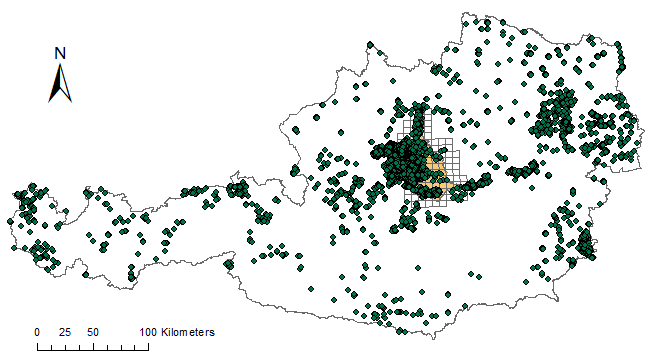


**Figure S1:** Distribution of the 12,498 vegetation plots across Austria. Plots clustered in and around the study area are mostly derived from Office of the State of Upper Austria (1993-2013).


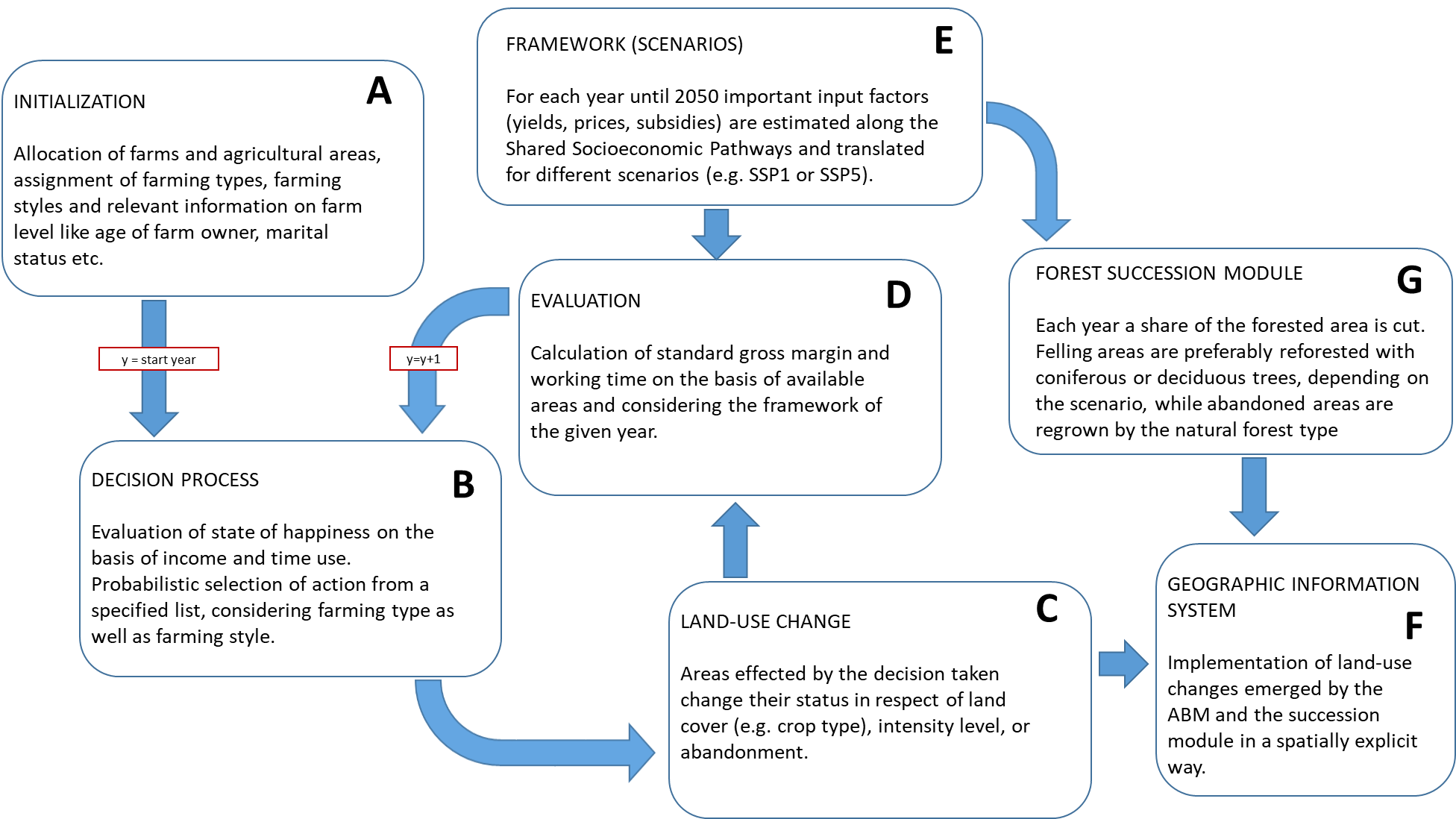


**Figure S2:** Overview of the modelling process and data processing in the Agent Based Model.

**Figure S3:** Projected changes in the exposure of the 832 modelled species in the study region. Exposure is calculated as C – O / C, where C is the currently suitable area and O is the overlap between the area suitable under both current and under future conditions, and thus, measures the spatial stability of ranges (Choe *et al.*, 2017). Changes are depicted for different combinations of land-use and climate change scenarios: (a)-(c) under current climate (‘CURRENT’) but varying land-use scenarios, (d)-(f) under varying climate but current land use (‘REF’), and (h)-(i) under varying climate and the SSP5 land-use scenario. Barplots depict mean values of five simulation runs for each scenario, while error bars depict the minimum and maximum changes from these five simulation runs.

**Table S1.** Information on vegetation plot data. The table lists, for all vegetation plot databases, the number of plot records, the year of sampling, the accuracy of spatial localization, and information on how land-use classes have been assigned to the plots.

| **Database** | **Pascher *et al.* (2011)** | **Office of the State of Upper Austria (1993-2013)** | **Willner *et al.* (2012)** | **own records** |
| --- | --- | --- | --- | --- |
| **Number of plots** | 1,786 | 3,938 | 6,619 | 155 |
| **Year of sampling** | 2007 | 1993-2013 | 1991-2014 | 2016 |
| **Accuracy of geographical localisation** | c. 20 m | Plots were assigned to the centre of gravity of the biotope polygons they characterize. | < 250 m | < 10 m |
| **Plot size** | Plots vary in size between 2 and 76 m^2^. | Not consistently documented, but mostly between 10 and 1,000 m². Recorded plots characterize larger polygons which themselves  vary in size between 50 and 100,000 m^2^. | Plots vary in size between 3 and 1,920 m^2^. | 25 m^2^ |
| **Assignment of land-use information (see Tab. 1) to plots** | Direct classification in the field | Direct classification in the field | Land use information was derived from available phytosociological classification of each plot | Direct classification in the field |

**Table S2.** List of modelled species, information on model performance and habitat group assignment.
The table lists, for all species and each modelling technique, the mean True Skill Statistics value (TSS) over all SDM replications, the number of failed replicate runs, the number of replicates with TSS < 0.5, and the TSS value of the Ensemble Model. Additionally, information on habitat specialization of species is given: species have been assigned to one of four habitat groups (forests, alpine habitats, agricultural lands, grasslands) when having at least 75 % of their occurrences in vegetation plots assigned to this group (a blank indciates that the species has less than 75 % of their occurrences in plots assigned to any of these groups).

| **Species** | **RF: mean TSS** | **RF: failed  / TSS < 0.5** | **GBM: mean TSS** | **GBM: failed / TSS < 0.5** | **ANN: mean TSS** | **ANN: failed  / TSS < 0.5** | **Ensemble Model: TSS** | **Habitat group** |
| --- | --- | --- | --- | --- | --- | --- | --- | --- |
| Abies alba | 0.773 | 0 / 0 | 0.774 | 0 / 0 | 0.764 | 0 / 0 | 0.85 | forests |
| Acer campestre | 0.825 | 0 / 0 | 0.808 | 0 / 0 | 0.784 | 0 / 0 | 0.887 | forests |
| Acer platanoides | 0.751 | 0 / 0 | 0.715 | 0 / 0 | 0.681 | 0 / 0 | 0.858 | forests |
| Acer pseudoplatanus | 0.765 | 0 / 0 | 0.746 | 0 / 0 | 0.742 | 0 / 0 | 0.797 | forests |
| Achillea atrata | 0.884 | 0 / 0 | 0.912 | 0 / 0 | 0.821 | 0 / 0 | 0.939 | alpine habitats |
| Achillea clavennae | 0.849 | 0 / 0 | 0.858 | 0 / 0 | 0.84 | 0 / 0 | 0.915 | alpine habitats |
| Achillea clusiana | 0.785 | 0 / 0 | 0.899 | 0 / 0 | 0.852 | 0 / 0 | 0.921 | alpine habitats |
| Achillea millefolium agg. | 0.762 | 0 / 0 | 0.75 | 0 / 0 | 0.735 | 0 / 0 | 0.8 |  |
| Aconitum lycoctonum | 0.751 | 0 / 0 | 0.73 | 0 / 0 | 0.712 | 0 / 0 | 0.865 | forests |
| Aconitum napellus agg. | 0.722 | 0 / 0 | 0.746 | 0 / 0 | 0.732 | 0 / 0 | 0.85 |  |
| Aconitum variegatum agg. | 0.595 | 0 / 0 | 0.737 | 0 / 0 | 0.682 | 0 / 0 | 0.834 |  |
| Actaea spicata | 0.687 | 0 / 0 | 0.708 | 0 / 0 | 0.66 | 0 / 0 | 0.849 | forests |
| Adenostyles alliariae | 0.722 | 0 / 0 | 0.763 | 0 / 0 | 0.687 | 0 / 0 | 0.882 |  |
| Adenostyles alpina | 0.763 | 0 / 0 | 0.744 | 0 / 0 | 0.721 | 0 / 0 | 0.867 |  |
| Adoxa moschatellina | 0.784 | 0 / 0 | 0.829 | 0 / 0 | 0.811 | 0 / 0 | 0.947 | forests |
| Aegopodium podagraria | 0.693 | 0 / 0 | 0.662 | 0 / 0 | 0.669 | 0 / 0 | 0.791 |  |
| Aethusa cynapium | 0.503 | 0 / 0 | 0.79 | 0 / 0 | 0.582 | 0 / 0 | 0.914 | agricultural lands |
| Agrimonia eupatoria | 0.767 | 0 / 0 | 0.62 | 0 / 0 | 0.61 | 0 / 0 | 0.887 |  |
| Agrostis alpina | 0.846 | 0 / 0 | 0.898 | 0 / 0 | 0.874 | 0 / 0 | 0.93 | alpine habitats |
| Agrostis canina | 0.752 | 0 / 0 | 0.775 | 0 / 0 | 0.78 | 1 / 0 | 0.853 | grasslands |
| Agrostis capillaris | 0.647 | 0 / 0 | 0.596 | 0 / 0 | 0.533 | 0 / 1 | 0.832 |  |
| Agrostis rupestris | 0.822 | 0 / 0 | 0.863 | 0 / 0 | 0.858 | 0 / 0 | 0.938 | alpine habitats |
| Agrostis stolonifera | 0.624 | 0 / 0 | 0.578 | 0 / 0 | 0.542 | 0 / 1 | 0.857 |  |
| Ajuga reptans | 0.628 | 0 / 0 | 0.538 | 0 / 0 | 0.532 | 0 / 0 | 0.767 |  |
| Alliaria petiolata | 0.815 | 0 / 0 | 0.829 | 0 / 0 | 0.687 | 0 / 0 | 0.926 | forests |
| Allium ursinum | 0.555 | 0 / 0 | 0.738 | 0 / 0 | 0.655 | 0 / 0 | 0.88 | forests |
| Allium victorialis | 0.924 | 0 / 0 | 0.936 | 0 / 0 | 0.935 | 0 / 0 | 0.981 | alpine habitats |
| Alnus glutinosa | 0.695 | 0 / 0 | 0.659 | 0 / 0 | 0.687 | 0 / 0 | 0.811 |  |
| Alnus incana | 0.705 | 0 / 0 | 0.613 | 0 / 0 | 0.587 | 0 / 0 | 0.818 |  |
| Alopecurus pratensis | 0.842 | 0 / 0 | 0.759 | 0 / 0 | 0.748 | 0 / 0 | 0.888 |  |
| Amaranthus retroflexus | 0.878 | 0 / 0 | 0.91 | 0 / 0 | 0.877 | 0 / 0 | 0.955 | agricultural lands |
| Amelanchier ovalis | 0.688 | 0 / 0 | 0.763 | 0 / 0 | 0.689 | 1 / 0 | 0.872 | forests |
| Anagallis arvensis | 0.769 | 0 / 0 | 0.877 | 0 / 0 | 0.749 | 0 / 0 | 0.934 | agricultural lands |
| Andromeda polifolia | 0.734 | 0 / 0 | 0.871 | 0 / 0 | 0.855 | 0 / 0 | 0.892 | grasslands |
| Androsace chamaejasme | 0.903 | 0 / 0 | 0.936 | 0 / 0 | 0.877 | 0 / 0 | 0.967 | alpine habitats |
| Anemonastrum narcissiflorum | 0.875 | 0 / 0 | 0.886 | 0 / 0 | 0.855 | 0 / 0 | 0.947 | alpine habitats |
| Anemone nemorosa | 0.679 | 0 / 0 | 0.616 | 0 / 0 | 0.58 | 0 / 0 | 0.789 |  |
| Angelica sylvestris | 0.579 | 0 / 0 | 0.569 | 0 / 0 | 0.558 | 0 / 0 | 0.761 |  |
| Antennaria dioica | NA | 0 / 3 | 0.576 | 0 / 0 | 0.634 | 1 / 1 | 0.837 |  |
| Anthemis arvensis | 0.944 | 0 / 0 | 0.831 | 0 / 0 | 0.836 | 0 / 0 | 0.949 | agricultural lands |
| Anthericum ramosum | 0.664 | 0 / 0 | 0.661 | 0 / 0 | 0.679 | 0 / 0 | 0.855 |  |
| Anthoxanthum alpinum | 0.86 | 0 / 0 | 0.893 | 0 / 0 | 0.878 | 0 / 0 | 0.926 | alpine habitats |
| Anthoxanthum odoratum | 0.766 | 0 / 0 | 0.681 | 0 / 0 | 0.693 | 0 / 0 | 0.79 | grasslands |
| Anthriscus nitidus | 0.715 | 0 / 1 | 0.732 | 0 / 0 | 0.715 | 2 / 0 | 0.863 | forests |
| Anthriscus sylvestris s. str. | 0.675 | 0 / 0 | 0.658 | 0 / 0 | 0.563 | 0 / 0 | 0.852 |  |
| Anthyllis vulneraria subsp. alpicola | 0.865 | 0 / 0 | 0.878 | 0 / 0 | 0.824 | 0 / 0 | 0.934 | alpine habitats |
| Anthyllis vulneraria | 0.854 | 0 / 0 | 0.851 | 0 / 0 | 0.869 | 0 / 0 | 0.924 | grasslands |
| Apera spica-venti | 0.843 | 0 / 0 | 0.826 | 0 / 0 | 0.794 | 0 / 0 | 0.94 | agricultural lands |
| Aphanes arvensis | 0.873 | 0 / 0 | 0.876 | 0 / 0 | 0.714 | 0 / 0 | 0.967 | agricultural lands |
| Aposeris foetida | 0.685 | 0 / 0 | 0.708 | 0 / 0 | 0.673 | 0 / 0 | 0.908 | forests |
| Aquilegia atrata | 0.69 | 0 / 0 | 0.696 | 0 / 0 | 0.627 | 0 / 0 | 0.841 |  |
| Arabidopsis arenosa | 0.629 | 0 / 0 | 0.63 | 0 / 0 | 0.57 | 0 / 0 | 0.832 |  |
| Arabidopsis thaliana | 0.915 | 0 / 0 | 0.814 | 0 / 0 | 0.753 | 0 / 0 | 0.931 |  |
| Arabis alpina subsp. alpina | 0.682 | 0 / 0 | 0.755 | 0 / 0 | 0.746 | 0 / 0 | 0.891 |  |
| Arabis ciliata | 0.553 | 0 / 0 | 0.662 | 0 / 0 | 0.568 | 0 / 2 | 0.89 |  |
| Arabis hirsuta s. str. | 0.668 | 0 / 0 | 0.77 | 0 / 0 | 0.72 | 0 / 0 | 0.927 | grasslands |
| Arabis pumila agg. | 0.822 | 0 / 0 | 0.837 | 0 / 0 | 0.805 | 0 / 0 | 0.925 | alpine habitats |
| Arctium lappa | 0.672 | 0 / 0 | 0.672 | 0 / 0 | 0.566 | 0 / 1 | 0.893 |  |
| Arctostaphylos alpinus | 0.798 | 0 / 0 | 0.872 | 0 / 0 | 0.775 | 0 / 0 | 0.943 | alpine habitats |
| Arenaria ciliata s. str. | 0.823 | 0 / 0 | 0.919 | 0 / 0 | 0.888 | 0 / 0 | 0.945 | alpine habitats |
| Arenaria serpyllifolia s. str. | 0.778 | 0 / 0 | 0.737 | 0 / 0 | 0.752 | 0 / 0 | 0.891 |  |
| Armeria alpina s. str. | 0.92 | 0 / 0 | 0.916 | 0 / 0 | 0.86 | 0 / 0 | 0.96 | alpine habitats |
| Arnica montana | 0.795 | 0 / 0 | 0.831 | 0 / 0 | 0.791 | 0 / 0 | 0.905 |  |
| Arrhenatherum elatius | 0.774 | 0 / 0 | 0.717 | 0 / 0 | 0.706 | 0 / 0 | 0.806 |  |
| Aruncus dioicus | 0.664 | 0 / 0 | 0.681 | 0 / 0 | 0.681 | 0 / 0 | 0.832 | forests |
| Asarum europaeum | 0.71 | 0 / 0 | 0.72 | 0 / 0 | 0.707 | 0 / 0 | 0.82 | forests |
| Asperula cynanchica s. str. | 0.806 | 0 / 0 | 0.829 | 0 / 0 | 0.768 | 0 / 0 | 0.947 | grasslands |
| Asplenium ruta-muraria | 0.697 | 0 / 0 | 0.654 | 0 / 0 | 0.687 | 0 / 0 | 0.86 |  |
| Asplenium scolopendrium | 0.612 | 0 / 1 | 0.768 | 0 / 0 | 0.572 | 2 / 0 | 0.861 | forests |
| Asplenium trichomanes | 0.69 | 0 / 0 | 0.657 | 0 / 0 | 0.65 | 0 / 0 | 0.846 | forests |
| Asplenium viride | 0.711 | 0 / 0 | 0.751 | 0 / 0 | 0.75 | 0 / 0 | 0.852 |  |
| Aster alpinus | 0.708 | 0 / 0 | 0.864 | 0 / 0 | 0.627 | 0 / 1 | 0.958 | alpine habitats |
| Astragalus glycyphyllos | 0.613 | 0 / 0 | 0.546 | 0 / 1 | NA | 1 / 2 | 0.91 |  |
| Astrantia major | 0.691 | 0 / 0 | 0.674 | 0 / 0 | 0.647 | 0 / 0 | 0.781 |  |
| Athamanta cretensis | 0.873 | 0 / 0 | 0.873 | 0 / 0 | 0.805 | 0 / 0 | 0.949 | alpine habitats |
| Athyrium filix-femina | 0.689 | 0 / 0 | 0.688 | 0 / 0 | 0.66 | 0 / 0 | 0.813 | forests |
| Atropa bella-donna | 0.719 | 0 / 0 | 0.78 | 0 / 0 | 0.723 | 1 / 0 | 0.854 | forests |
| Avena fatua | 0.938 | 0 / 0 | 0.885 | 0 / 0 | 0.88 | 0 / 0 | 0.945 | agricultural lands |
| Avenella flexuosa | 0.659 | 0 / 0 | 0.596 | 0 / 0 | 0.59 | 0 / 0 | 0.861 |  |
| Avenula versicolor | 0.805 | 0 / 0 | 0.939 | 0 / 0 | 0.909 | 0 / 0 | 0.973 | alpine habitats |
| Ballota nigra | 0.932 | 0 / 0 | 0.886 | 0 / 0 | 0.869 | 0 / 0 | 0.958 |  |
| Bartsia alpina | 0.798 | 0 / 0 | 0.833 | 0 / 0 | 0.848 | 0 / 0 | 0.884 | alpine habitats |
| Bellidiastrum michelii | 0.62 | 0 / 0 | 0.646 | 0 / 0 | 0.63 | 0 / 0 | 0.796 |  |
| Bellis perennis | 0.764 | 0 / 0 | 0.746 | 0 / 0 | 0.688 | 0 / 0 | 0.884 | grasslands |
| Berberis vulgaris | 0.642 | 0 / 0 | 0.696 | 0 / 0 | 0.688 | 0 / 0 | 0.822 |  |
| Betonica alopecuros | 0.703 | 0 / 0 | 0.73 | 0 / 0 | 0.695 | 0 / 0 | 0.871 |  |
| Betonica officinalis | 0.756 | 0 / 0 | 0.751 | 0 / 0 | 0.714 | 0 / 0 | 0.831 | grasslands |
| Betula pendula | 0.536 | 0 / 0 | 0.536 | 0 / 0 | 0.556 | 0 / 0 | 0.737 | forests |
| Betula pubescens | 0.605 | 0 / 1 | 0.826 | 0 / 0 | 0.624 | 0 / 0 | 0.899 | grasslands |
| Biscutella laevigata | 0.891 | 0 / 0 | 0.884 | 0 / 0 | 0.873 | 0 / 0 | 0.919 | alpine habitats |
| Blechnum spicant | 0.696 | 0 / 0 | 0.71 | 0 / 0 | 0.699 | 0 / 0 | 0.85 | forests |
| Botrychium lunaria | 0.732 | 0 / 0 | 0.867 | 0 / 0 | 0.814 | 0 / 0 | 0.955 | alpine habitats |
| Brachypodium pinnatum | 0.709 | 0 / 0 | 0.653 | 0 / 0 | 0.623 | 0 / 0 | 0.809 |  |
| Brachypodium sylvaticum | 0.705 | 0 / 0 | 0.73 | 0 / 0 | 0.714 | 0 / 0 | 0.838 | forests |
| Briza media | 0.72 | 0 / 0 | 0.711 | 0 / 0 | 0.725 | 0 / 0 | 0.797 | grasslands |
| Bromus benekenii | 0.693 | 0 / 0 | 0.687 | 0 / 0 | 0.718 | 0 / 0 | 0.858 | forests |
| Bromus erectus | 0.913 | 0 / 0 | 0.87 | 0 / 0 | 0.861 | 0 / 0 | 0.919 | grasslands |
| Bromus hordeaceus | 0.768 | 0 / 0 | 0.725 | 0 / 0 | 0.703 | 0 / 0 | 0.86 |  |
| Bromus sterilis | 0.959 | 0 / 0 | 0.895 | 0 / 0 | 0.886 | 0 / 0 | 0.951 | agricultural lands |
| Bromus tectorum | 0.955 | 0 / 0 | 0.925 | 0 / 0 | 0.88 | 0 / 0 | 0.963 | agricultural lands |
| Buphthalmum salicifolium | 0.709 | 0 / 0 | 0.69 | 0 / 0 | 0.686 | 0 / 0 | 0.823 |  |
| Calamagrostis canescens | 0.946 | 0 / 0 | 0.942 | 0 / 0 | 0.968 | 0 / 0 | 0.989 | grasslands |
| Calamagrostis epigejos | 0.514 | 0 / 1 | NA | 0 / 3 | NA | 0 / 3 | 0.922 |  |
| Calamagrostis varia | 0.732 | 0 / 0 | 0.742 | 0 / 0 | 0.736 | 0 / 0 | 0.795 | forests |
| Calamagrostis villosa | 0.646 | 0 / 1 | 0.738 | 0 / 0 | 0.533 | 0 / 2 | 0.851 |  |
| Calluna vulgaris | 0.695 | 0 / 0 | 0.722 | 0 / 0 | 0.698 | 0 / 0 | 0.769 | grasslands |
| Caltha palustris | 0.528 | 0 / 0 | 0.523 | 0 / 1 | 0.554 | 0 / 1 | 0.777 |  |
| Calystegia sepium s. str. | 0.707 | 0 / 0 | 0.752 | 0 / 0 | 0.69 | 0 / 0 | 0.884 |  |
| Camelina microcarpa | 0.931 | 0 / 0 | 0.919 | 0 / 0 | 0.9 | 0 / 0 | 0.962 | agricultural lands |
| Campanula alpina | 0.904 | 0 / 0 | 0.927 | 0 / 0 | 0.898 | 0 / 0 | 0.952 | alpine habitats |
| Campanula barbata | 0.852 | 0 / 0 | 0.883 | 0 / 0 | 0.845 | 0 / 0 | 0.976 | alpine habitats |
| Campanula cespitosa | 0.63 | 0 / 0 | 0.681 | 0 / 0 | 0.568 | 0 / 0 | 0.894 |  |
| Campanula cochleariifolia | 0.71 | 0 / 0 | 0.745 | 0 / 0 | 0.734 | 0 / 0 | 0.868 |  |
| Campanula patula | 0.771 | 0 / 0 | 0.776 | 0 / 0 | 0.761 | 0 / 0 | 0.854 | grasslands |
| Campanula persicifolia | 0.653 | 0 / 0 | 0.703 | 0 / 0 | 0.664 | 0 / 0 | 0.835 |  |
| Campanula pulla | 0.848 | 0 / 0 | 0.87 | 0 / 0 | 0.852 | 0 / 0 | 0.932 | alpine habitats |
| Campanula rapunculoides | 0.602 | 0 / 0 | 0.631 | 0 / 0 | 0.596 | 0 / 1 | 0.851 |  |
| Campanula rotundifolia | 0.634 | 0 / 0 | 0.588 | 0 / 0 | 0.582 | 0 / 0 | 0.832 |  |
| Campanula scheuchzeri | 0.779 | 0 / 0 | 0.787 | 0 / 0 | 0.795 | 0 / 0 | 0.849 | alpine habitats |
| Campanula trachelium | 0.668 | 0 / 0 | 0.631 | 0 / 0 | 0.624 | 0 / 0 | 0.818 | forests |
| Capsella bursa-pastoris | 0.816 | 0 / 0 | 0.825 | 0 / 0 | 0.812 | 0 / 0 | 0.898 | agricultural lands |
| Cardamine amara | 0.588 | 0 / 0 | 0.598 | 0 / 0 | 0.52 | 0 / 1 | 0.806 |  |
| Cardamine bulbifera | 0.713 | 0 / 0 | 0.752 | 0 / 0 | 0.676 | 0 / 0 | 0.877 | forests |
| Cardamine enneaphyllos | 0.782 | 0 / 0 | 0.796 | 0 / 0 | 0.815 | 0 / 0 | 0.863 | forests |
| Cardamine flexuosa | 0.505 | 0 / 1 | 0.703 | 1 / 1 | 0.546 | 0 / 0 | 0.816 | forests |
| Cardamine impatiens | 0.595 | 0 / 0 | 0.744 | 0 / 0 | 0.702 | 0 / 0 | 0.822 | forests |
| Cardamine pratensis agg. | 0.754 | 0 / 0 | 0.684 | 0 / 0 | 0.681 | 1 / 0 | 0.844 | grasslands |
| Cardamine trifolia | 0.787 | 0 / 0 | 0.781 | 0 / 0 | 0.784 | 0 / 0 | 0.861 | forests |
| Carduus acanthoides | 0.907 | 0 / 0 | 0.884 | 0 / 0 | 0.873 | 0 / 0 | 0.94 | agricultural lands |
| Carduus defloratus s. lat. | 0.715 | 0 / 0 | 0.708 | 0 / 0 | 0.728 | 0 / 0 | 0.824 |  |
| Carex acuta | 0.79 | 0 / 0 | 0.87 | 0 / 0 | 0.853 | 0 / 0 | 0.94 | grasslands |
| Carex acutiformis | 0.743 | 0 / 0 | 0.755 | 0 / 0 | 0.714 | 0 / 0 | 0.875 |  |
| Carex alba | 0.807 | 0 / 0 | 0.788 | 0 / 0 | 0.781 | 0 / 0 | 0.832 | forests |
| Carex atrata | 0.857 | 0 / 0 | 0.868 | 0 / 0 | 0.823 | 0 / 0 | 0.942 | alpine habitats |
| Carex brachystachys | 0.697 | 0 / 1 | 0.723 | 0 / 0 | 0.722 | 0 / 1 | 0.874 | forests |
| Carex brizoides | 0.791 | 0 / 0 | 0.763 | 0 / 0 | 0.7 | 0 / 0 | 0.855 |  |
| Carex canescens | 0.596 | 0 / 1 | 0.776 | 0 / 0 | 0.606 | 1 / 0 | 0.86 | grasslands |
| Carex capillaris | 0.838 | 0 / 0 | 0.84 | 0 / 0 | 0.846 | 0 / 0 | 0.916 | alpine habitats |
| Carex caryophyllea | 0.784 | 0 / 0 | 0.83 | 0 / 0 | 0.848 | 0 / 0 | 0.894 | grasslands |
| Carex curvula | 0.927 | 0 / 0 | 0.918 | 0 / 0 | 0.912 | 0 / 0 | 0.967 | alpine habitats |
| Carex davalliana | 0.882 | 0 / 0 | 0.807 | 1 / 0 | 0.814 | 0 / 0 | 0.861 | grasslands |
| Carex digitata | 0.66 | 0 / 0 | 0.621 | 0 / 0 | 0.628 | 0 / 0 | 0.854 | forests |
| Carex echinata | 0.733 | 0 / 0 | 0.796 | 0 / 0 | 0.797 | 0 / 0 | 0.836 | grasslands |
| Carex elata | 0.854 | 0 / 0 | 0.881 | 0 / 0 | 0.87 | 0 / 0 | 0.918 | grasslands |
| Carex ferruginea | 0.749 | 0 / 0 | 0.787 | 0 / 0 | 0.743 | 0 / 0 | 0.879 |  |
| Carex firma | 0.845 | 0 / 0 | 0.875 | 0 / 0 | 0.846 | 0 / 0 | 0.92 | alpine habitats |
| Carex flacca | 0.623 | 0 / 0 | 0.552 | 0 / 0 | 0.556 | 0 / 0 | 0.758 |  |
| Carex flava agg. | 0.644 | 0 / 0 | 0.706 | 0 / 0 | 0.703 | 0 / 0 | 0.796 | grasslands |
| Carex fuliginosa | 0.89 | 0 / 0 | 0.906 | 0 / 0 | 0.908 | 0 / 0 | 0.953 | alpine habitats |
| Carex hirta | 0.628 | 0 / 0 | 0.642 | 0 / 0 | 0.516 | 0 / 0 | 0.823 |  |
| Carex hostiana | 0.792 | 0 / 0 | 0.821 | 0 / 0 | 0.82 | 0 / 0 | 0.88 | grasslands |
| Carex humilis | 0.653 | 0 / 0 | 0.692 | 0 / 0 | 0.66 | 0 / 0 | 0.892 |  |
| Carex leporina | 0.51 | 0 / 2 | 0.646 | 0 / 1 | NA | 1 / 2 | 0.788 |  |
| Carex limosa | 0.675 | 0 / 0 | 0.848 | 0 / 0 | 0.8 | 0 / 0 | 0.896 | grasslands |
| Carex montana | 0.671 | 0 / 0 | 0.655 | 0 / 0 | 0.618 | 0 / 0 | 0.867 |  |
| Carex mucronata | 0.714 | 0 / 0 | 0.75 | 0 / 0 | 0.738 | 0 / 0 | 0.897 | alpine habitats |
| Carex muricata agg. | 0.611 | 0 / 0 | 0.638 | 0 / 0 | 0.51 | 0 / 0 | 0.826 |  |
| Carex nigra | 0.762 | 0 / 0 | 0.778 | 0 / 0 | 0.762 | 0 / 0 | 0.836 | grasslands |
| Carex ornithopoda | 0.573 | 0 / 0 | 0.623 | 0 / 0 | 0.668 | 1 / 0 | 0.847 |  |
| Carex pallescens | 0.702 | 0 / 0 | 0.665 | 0 / 0 | 0.611 | 0 / 0 | 0.808 | grasslands |
| Carex panicea | 0.764 | 0 / 0 | 0.752 | 0 / 0 | 0.73 | 0 / 0 | 0.827 | grasslands |
| Carex paniculata | 0.664 | 0 / 0 | 0.664 | 0 / 0 | 0.578 | 0 / 0 | 0.836 | grasslands |
| Carex parviflora | 0.653 | 0 / 0 | 0.844 | 0 / 0 | 0.788 | 0 / 0 | 0.958 | alpine habitats |
| Carex pauciflora | 0.822 | 0 / 0 | 0.872 | 0 / 0 | 0.883 | 0 / 0 | 0.908 | grasslands |
| Carex pendula | 0.689 | 0 / 0 | 0.713 | 0 / 0 | 0.687 | 0 / 0 | 0.856 | forests |
| Carex pilosa | 0.815 | 0 / 0 | 0.851 | 0 / 0 | 0.774 | 0 / 0 | 0.912 | forests |
| Carex pilulifera | 0.614 | 0 / 0 | 0.691 | 0 / 0 | 0.678 | 0 / 1 | 0.871 |  |
| Carex praecox | 0.941 | 0 / 0 | 0.928 | 0 / 0 | 0.917 | 0 / 0 | 0.973 | grasslands |
| Carex pulicaris | 0.822 | 0 / 0 | 0.854 | 0 / 0 | 0.8 | 0 / 0 | 0.94 | grasslands |
| Carex remota | 0.549 | 0 / 0 | 0.663 | 0 / 0 | 0.603 | 0 / 1 | 0.838 | forests |
| Carex rostrata | 0.836 | 0 / 0 | 0.847 | 0 / 0 | 0.829 | 0 / 0 | 0.866 | grasslands |
| Carex sempervirens | 0.807 | 0 / 0 | 0.843 | 0 / 0 | 0.829 | 0 / 0 | 0.893 | alpine habitats |
| Carex sylvatica | 0.694 | 0 / 0 | 0.65 | 0 / 0 | 0.658 | 0 / 0 | 0.806 | forests |
| Carex tomentosa | 0.697 | 0 / 0 | 0.675 | 0 / 0 | 0.644 | 0 / 0 | 0.905 | grasslands |
| Carex umbrosa | 0.557 | 0 / 2 | 0.691 | 0 / 0 | 0.621 | 1 / 1 | 0.818 |  |
| Carex vesicaria | 0.666 | 0 / 0 | 0.761 | 0 / 0 | 0.734 | 0 / 0 | 0.93 | grasslands |
| Carex vulpina agg. | 0.625 | 0 / 0 | 0.708 | 0 / 0 | 0.679 | 0 / 0 | 0.927 | grasslands |
| Carlina acaulis | 0.786 | 0 / 0 | 0.787 | 0 / 0 | 0.721 | 0 / 0 | 0.884 |  |
| Carpinus betulus | 0.766 | 0 / 0 | 0.707 | 0 / 0 | 0.747 | 0 / 0 | 0.842 | forests |
| Carum carvi | 0.714 | 0 / 0 | 0.728 | 0 / 0 | 0.7 | 0 / 0 | 0.878 | grasslands |
| Castanea sativa^[[1]](#footnote-1)^ | NA |  | NA | 3 / 0 | NA | 3 /0 | NA |  |
| Centaurea jacea | 0.787 | 0 / 0 | 0.758 | 0 / 0 | 0.755 | 0 / 0 | 0.834 | grasslands |
| Centaurea scabiosa | 0.786 | 0 / 0 | 0.735 | 0 / 0 | 0.725 | 0 / 0 | 0.863 | grasslands |
| Centaurea stoebe | 0.789 | 0 / 0 | 0.841 | 0 / 0 | 0.757 | 0 / 0 | 0.967 | grasslands |
| Cephalanthera damasonium | 0.546 | 0 / 1 | 0.739 | 0 / 0 | 0.624 | 1 / 0 | 0.812 | forests |
| Cephalanthera longifolia | 0.539 | 0 / 0 | 0.615 | 0 / 0 | 0.649 | 1 / 1 | 0.861 | forests |
| Cerastium alpinum agg. | 0.927 | 0 / 0 | 0.941 | 0 / 0 | 0.835 | 1 / 0 | 0.987 | alpine habitats |
| Cerastium arvense s. lat. | 0.805 | 0 / 0 | 0.822 | 0 / 0 | 0.796 | 0 / 0 | 0.873 |  |
| Cerastium carinthiacum | 0.869 | 0 / 0 | 0.925 | 0 / 0 | 0.845 | 0 / 0 | 0.932 | alpine habitats |
| Cerastium holosteoides | 0.773 | 0 / 0 | 0.738 | 0 / 0 | 0.736 | 0 / 0 | 0.824 |  |
| Chaerophyllum aureum | 0.7 | 0 / 0 | 0.698 | 0 / 0 | 0.626 | 0 / 0 | 0.887 |  |
| Chaerophyllum hirsutum s. str. | 0.661 | 0 / 0 | 0.626 | 0 / 0 | 0.638 | 0 / 0 | 0.793 |  |
| Chaerophyllum villarsii | 0.566 | 0 / 1 | 0.699 | 0 / 0 | 0.548 | 0 / 1 | 0.825 |  |
| Chamaecytisus supinus | 0.699 | 0 / 0 | 0.705 | 0 / 0 | 0.72 | 0 / 0 | 0.958 |  |
| Chelidonium majus | 0.655 | 0 / 0 | 0.552 | 0 / 0 | 0.553 | 1 / 0 | 0.897 |  |
| Chenopodium album agg. | 0.881 | 0 / 0 | 0.874 | 0 / 0 | 0.866 | 0 / 0 | 0.925 | agricultural lands |
| Chenopodium hybridum | 0.913 | 0 / 0 | 0.91 | 0 / 0 | 0.815 | 0 / 0 | 0.952 | agricultural lands |
| Chenopodium polyspermum | 0.921 | 0 / 0 | 0.745 | 0 / 0 | 0.708 | 0 / 0 | 0.974 | agricultural lands |
| Chrysosplenium alternifolium | 0.633 | 0 / 0 | 0.737 | 0 / 0 | 0.632 | 0 / 0 | 0.83 | forests |
| Cichorium intybus | 0.837 | 0 / 0 | 0.814 | 0 / 0 | 0.703 | 0 / 0 | 0.97 | agricultural lands |
| Circaea lutetiana | 0.683 | 0 / 0 | 0.733 | 0 / 0 | 0.713 | 0 / 0 | 0.827 | forests |
| Cirsium arvense | 0.626 | 0 / 0 | 0.592 | 0 / 0 | 0.558 | 0 / 0 | 0.803 |  |
| Cirsium erisithales | 0.693 | 0 / 0 | 0.67 | 0 / 0 | 0.661 | 0 / 0 | 0.841 | forests |
| Cirsium oleraceum | 0.616 | 0 / 0 | 0.604 | 0 / 0 | 0.557 | 0 / 0 | 0.78 |  |
| Cirsium palustre | 0.526 | 0 / 0 | 0.581 | 0 / 0 | 0.53 | 0 / 0 | 0.769 |  |
| Cirsium pannonicum | 0.871 | 0 / 0 | 0.917 | 0 / 0 | 0.78 | 0 / 0 | 0.955 | grasslands |
| Cirsium rivulare | 0.714 | 0 / 0 | 0.801 | 0 / 0 | 0.796 | 1 / 0 | 0.919 | grasslands |
| Cirsium vulgare | 0.618 | 0 / 0 | 0.605 | 0 / 1 | NA | 1 / 2 | 0.933 |  |
| Clematis alpina | 0.842 | 0 / 0 | 0.864 | 0 / 0 | 0.668 | 0 / 0 | 0.913 | forests |
| Clematis vitalba | 0.694 | 0 / 0 | 0.659 | 0 / 0 | 0.686 | 0 / 0 | 0.799 | forests |
| Clinopodium alpinum | 0.729 | 0 / 0 | 0.768 | 0 / 0 | 0.788 | 0 / 0 | 0.889 |  |
| Clinopodium vulgare | 0.602 | 0 / 0 | 0.551 | 0 / 0 | 0.551 | 0 / 0 | 0.799 |  |
| Coeloglossum viride | 0.73 | 0 / 0 | 0.795 | 0 / 0 | 0.699 | 0 / 0 | 0.87 | alpine habitats |
| Colchicum autumnale | 0.722 | 0 / 0 | 0.716 | 0 / 0 | 0.713 | 0 / 0 | 0.847 | grasslands |
| Comarum palustre | 0.584 | 0 / 0 | 0.802 | 0 / 0 | 0.777 | 0 / 0 | 0.897 | grasslands |
| Consolida regalis | 0.932 | 0 / 0 | 0.917 | 0 / 0 | 0.911 | 0 / 0 | 0.966 | agricultural lands |
| Convallaria majalis | 0.73 | 0 / 0 | 0.721 | 0 / 0 | 0.698 | 0 / 0 | 0.869 | forests |
| Convolvulus arvensis | 0.859 | 0 / 0 | 0.846 | 0 / 0 | 0.821 | 0 / 0 | 0.885 | agricultural lands |
| Cornus mas | 0.771 | 0 / 0 | 0.806 | 0 / 0 | 0.728 | 0 / 0 | 0.921 | forests |
| Cornus sanguinea | 0.709 | 0 / 0 | 0.702 | 0 / 0 | 0.699 | 0 / 0 | 0.814 |  |
| Corydalis solida | 0.837 | 0 / 0 | 0.946 | 0 / 0 | 0.771 | 0 / 0 | 0.975 | forests |
| Corylus avellana | 0.743 | 0 / 0 | 0.706 | 0 / 0 | 0.707 | 0 / 0 | 0.802 | forests |
| Crataegus laevigata | 0.675 | 0 / 0 | 0.749 | 0 / 0 | 0.726 | 0 / 0 | 0.848 |  |
| Crataegus monogyna | 0.663 | 0 / 0 | 0.632 | 0 / 0 | 0.622 | 0 / 0 | 0.794 |  |
| Crepis aurea | 0.809 | 0 / 0 | 0.797 | 0 / 0 | 0.748 | 0 / 0 | 0.88 |  |
| Crepis biennis | 0.804 | 0 / 0 | 0.74 | 0 / 0 | 0.75 | 0 / 0 | 0.877 | grasslands |
| Crepis mollis | 0.532 | 0 / 0 | 0.628 | 0 / 0 | 0.536 | 1 / 0 | 0.905 | grasslands |
| Crepis paludosa | 0.612 | 0 / 0 | 0.63 | 0 / 0 | 0.56 | 0 / 0 | 0.805 |  |
| Cruciata glabra | 0.946 | 0 / 0 | 0.919 | 0 / 0 | 0.787 | 0 / 0 | 0.969 | forests |
| Cruciata laevipes | 0.697 | 0 / 0 | 0.672 | 0 / 0 | 0.627 | 0 / 0 | 0.831 |  |
| Cuscuta epithymum | 0.562 | 0 / 1 | 0.779 | 0 / 0 | 0.591 | 0 / 1 | 0.855 | grasslands |
| Cyanus montanus | 0.64 | 0 / 0 | 0.714 | 1 / 0 | 0.633 | 0 / 0 | 0.865 | forests |
| Cyanus segetum | 0.923 | 0 / 0 | 0.88 | 0 / 0 | 0.862 | 0 / 0 | 0.964 | agricultural lands |
| Cyclamen purpurascens | 0.754 | 0 / 0 | 0.746 | 0 / 0 | 0.748 | 0 / 0 | 0.831 | forests |
| Cynosurus cristatus | 0.795 | 0 / 0 | 0.8 | 0 / 0 | 0.823 | 0 / 0 | 0.878 | grasslands |
| Cystopteris alpina | 0.811 | 0 / 0 | 0.908 | 0 / 0 | 0.754 | 0 / 0 | 0.972 | alpine habitats |
| Cystopteris fragilis s. str. | 0.743 | 0 / 0 | 0.784 | 0 / 0 | 0.674 | 0 / 0 | 0.887 |  |
| Dactylis glomerata | 0.68 | 0 / 0 | 0.637 | 0 / 0 | 0.637 | 0 / 0 | 0.78 |  |
| Dactylis polygama | 0.934 | 0 / 0 | 0.919 | 0 / 0 | 0.921 | 0 / 0 | 0.971 | forests |
| Dactylorhiza incarnata | 0.506 | 0 / 2 | 0.687 | 0 / 0 | 0.607 | 0 / 0 | 0.819 | grasslands |
| Dactylorhiza maculata s. lat. | 0.615 | 0 / 0 | 0.646 | 0 / 0 | 0.582 | 0 / 0 | 0.822 |  |
| Dactylorhiza majalis | 0.769 | 0 / 0 | 0.758 | 0 / 0 | 0.741 | 0 / 0 | 0.831 | grasslands |
| Danthonia decumbens | 0.719 | 0 / 0 | 0.769 | 0 / 0 | 0.71 | 0 / 0 | 0.88 | grasslands |
| Daphne laureola | 0.756 | 0 / 0 | 0.802 | 0 / 0 | 0.796 | 0 / 0 | 0.928 | forests |
| Daphne mezereum | 0.724 | 0 / 0 | 0.715 | 0 / 0 | 0.675 | 0 / 0 | 0.81 | forests |
| Daucus carota | 0.707 | 0 / 0 | 0.706 | 0 / 0 | 0.694 | 1 / 0 | 0.86 |  |
| Deschampsia cespitosa | 0.563 | 0 / 0 | NA | 0 / 3 | NA | 0 / 3 | 0.874 |  |
| Descurainia sophia | 0.948 | 0 / 0 | 0.921 | 0 / 0 | 0.906 | 0 / 0 | 0.966 | agricultural lands |
| Dianthus alpinus | 0.957 | 0 / 0 | 0.926 | 0 / 0 | 0.931 | 0 / 0 | 0.96 | alpine habitats |
| Dianthus carthusianorum agg. | 0.803 | 0 / 0 | 0.793 | 0 / 0 | 0.728 | 0 / 0 | 0.918 | grasslands |
| Digitalis grandiflora | 0.72 | 0 / 0 | 0.702 | 0 / 0 | 0.665 | 0 / 0 | 0.831 | forests |
| Doronicum austriacum | 0.588 | 0 / 0 | 0.688 | 0 / 0 | 0.723 | 0 / 0 | 0.897 | forests |
| Doronicum clusii agg. | 0.876 | 0 / 0 | 0.93 | 0 / 0 | 0.885 | 0 / 0 | 0.94 | alpine habitats |
| Dorycnium germanicum | 0.836 | 0 / 0 | 0.682 | 0 / 0 | 0.784 | 0 / 0 | 0.992 | grasslands |
| Drosera rotundifolia | 0.71 | 0 / 0 | 0.821 | 0 / 0 | 0.804 | 0 / 0 | 0.87 | grasslands |
| Dryas octopetala | 0.888 | 0 / 0 | 0.907 | 0 / 0 | 0.893 | 0 / 0 | 0.941 | alpine habitats |
| Dryopteris affinis s. lat. | 0.642 | 0 / 0 | 0.684 | 0 / 0 | 0.67 | 0 / 0 | 0.848 | forests |
| Dryopteris carthusiana s. str. | 0.527 | 0 / 0 | 0.647 | 0 / 0 | 0.618 | 0 / 0 | 0.786 | forests |
| Dryopteris dilatata | 0.68 | 0 / 0 | 0.757 | 0 / 0 | 0.685 | 0 / 0 | 0.854 | forests |
| Dryopteris filix-mas agg. | 0.756 | 0 / 0 | 0.843 | 0 / 0 | 0.66 | 0 / 0 | 0.937 | forests |
| Dryopteris filix-mas s. str. | 0.667 | 0 / 0 | 0.715 | 0 / 0 | 0.714 | 0 / 0 | 0.814 | forests |
| Echinochloa crus-galli | 0.912 | 0 / 0 | 0.86 | 0 / 0 | 0.868 | 0 / 0 | 0.959 | agricultural lands |
| Echium vulgare | 0.706 | 0 / 0 | 0.789 | 0 / 0 | 0.607 | 0 / 0 | 0.942 |  |
| Eleocharis palustris agg. | 0.587 | 0 / 1 | 0.794 | 0 / 0 | 0.632 | 0 / 0 | 0.892 | grasslands |
| Elymus caninus | 0.664 | 0 / 0 | 0.738 | 0 / 0 | 0.65 | 0 / 1 | 0.957 |  |
| Elymus repens | 0.814 | 0 / 0 | 0.791 | 0 / 0 | 0.777 | 0 / 0 | 0.868 |  |
| Empetrum nigrum agg. | 0.67 | 0 / 1 | 0.821 | 0 / 0 | 0.737 | 1 / 0 | 0.915 |  |
| Epilobium hirsutum | 0.51 | 0 / 2 | 0.584 | 0 / 2 | NA | 0 / 3 | 0.711 |  |
| Epilobium montanum | 0.588 | 0 / 0 | 0.573 | 0 / 0 | 0.547 | 0 / 0 | 0.808 | forests |
| Epilobium palustre | 0.634 | 0 / 1 | 0.726 | 0 / 0 | 0.667 | 1 / 0 | 0.857 | grasslands |
| Epilobium parviflorum | 0.553 | 0 / 2 | 0.544 | 0 / 1 | NA | 1 / 2 | 0.763 |  |
| Epipactis atrorubens | 0.72 | 0 / 0 | 0.788 | 0 / 0 | 0.689 | 0 / 0 | 0.84 |  |
| Epipactis helleborine s. lat. | 0.648 | 0 / 0 | 0.674 | 0 / 0 | 0.617 | 0 / 0 | 0.826 | forests |
| Epipactis palustris | 0.755 | 0 / 0 | 0.789 | 0 / 0 | 0.793 | 0 / 0 | 0.862 | grasslands |
| Equisetum arvense | 0.612 | 0 / 0 | 0.608 | 0 / 0 | 0.577 | 0 / 0 | 0.785 |  |
| Equisetum fluviatile | 0.693 | 0 / 0 | 0.78 | 0 / 0 | 0.81 | 0 / 0 | 0.875 | grasslands |
| Equisetum palustre | 0.771 | 0 / 0 | 0.762 | 0 / 0 | 0.741 | 0 / 0 | 0.812 | grasslands |
| Equisetum sylvaticum | 0.645 | 0 / 0 | 0.676 | 0 / 0 | 0.667 | 0 / 0 | 0.851 |  |
| Equisetum telmateia | 0.752 | 0 / 0 | 0.674 | 0 / 0 | 0.675 | 0 / 0 | 0.854 |  |
| Erica carnea | 0.681 | 0 / 0 | 0.672 | 0 / 0 | 0.658 | 0 / 0 | 0.856 |  |
| Erigeron annuus | 0.827 | 0 / 0 | 0.754 | 0 / 0 | 0.601 | 0 / 0 | 0.877 |  |
| Erigeron canadensis | 0.835 | 0 / 0 | 0.772 | 0 / 0 | 0.736 | 0 / 0 | 0.912 | agricultural lands |
| Eriophorum angustifolium | 0.761 | 0 / 0 | 0.835 | 0 / 0 | 0.831 | 0 / 0 | 0.841 | grasslands |
| Eriophorum latifolium | 0.833 | 0 / 0 | 0.825 | 0 / 0 | 0.814 | 0 / 0 | 0.875 | grasslands |
| Eriophorum vaginatum | 0.809 | 0 / 0 | 0.852 | 0 / 0 | 0.839 | 0 / 0 | 0.873 | grasslands |
| Erodium cicutarium s. str. | 0.844 | 0 / 0 | 0.748 | 0 / 0 | 0.73 | 0 / 0 | 0.938 | agricultural lands |
| Eryngium campestre | 0.904 | 0 / 0 | 0.87 | 0 / 0 | 0.789 | 0 / 0 | 0.957 |  |
| Euonymus europaeus | 0.767 | 0 / 0 | 0.663 | 0 / 0 | 0.689 | 0 / 0 | 0.839 |  |
| Euonymus latifolius | 0.664 | 0 / 0 | 0.744 | 0 / 0 | 0.592 | 2 / 0 | 0.887 | forests |
| Eupatorium cannabinum | 0.675 | 0 / 0 | 0.656 | 0 / 0 | 0.674 | 0 / 0 | 0.782 | forests |
| Euphorbia amygdaloides | 0.71 | 0 / 0 | 0.702 | 0 / 0 | 0.662 | 0 / 0 | 0.834 | forests |
| Euphorbia austriaca | 0.694 | 0 / 0 | 0.713 | 0 / 0 | 0.546 | 0 / 1 | 0.895 |  |
| Euphorbia cyparissias | 0.636 | 0 / 0 | 0.62 | 0 / 0 | 0.566 | 0 / 0 | 0.846 |  |
| Euphorbia dulcis | 0.669 | 0 / 0 | 0.785 | 0 / 0 | 0.605 | 0 / 0 | 0.842 | forests |
| Euphorbia esula s. str. | 0.675 | 0 / 0 | 0.622 | 0 / 0 | 0.562 | 0 / 0 | 0.874 |  |
| Euphorbia helioscopia | 0.895 | 0 / 0 | 0.825 | 0 / 0 | 0.635 | 0 / 0 | 0.922 | agricultural lands |
| Euphorbia verrucosa | 0.588 | 0 / 1 | 0.586 | 0 / 0 | 0.516 | 0 / 0 | 0.878 | grasslands |
| Euphrasia minima agg. | 0.875 | 0 / 0 | 0.862 | 0 / 0 | 0.863 | 0 / 0 | 0.93 | alpine habitats |
| Euphrasia officinalis agg. | 0.666 | 0 / 0 | 0.69 | 0 / 0 | 0.647 | 0 / 0 | 0.776 |  |
| Euphrasia salisburgensis s. str. | 0.824 | 0 / 0 | 0.848 | 0 / 0 | 0.799 | 0 / 0 | 0.904 | alpine habitats |
| Fagus sylvatica | 0.841 | 0 / 0 | 0.834 | 0 / 0 | 0.828 | 0 / 0 | 0.866 | forests |
| Falcaria vulgaris | 0.897 | 0 / 0 | 0.858 | 0 / 0 | 0.852 | 0 / 0 | 0.939 |  |
| Fallopia convolvulus | 0.9 | 0 / 0 | 0.89 | 0 / 0 | 0.837 | 0 / 0 | 0.92 | agricultural lands |
| Fallopia dumetorum | 0.862 | 0 / 0 | 0.925 | 0 / 0 | 0.87 | 0 / 0 | 0.989 | forests |
| Festuca amethystina | 0.758 | 0 / 0 | 0.814 | 0 / 0 | 0.666 | 0 / 0 | 0.935 |  |
| Festuca arundinacea | 0.792 | 0 / 0 | 0.72 | 0 / 0 | 0.643 | 0 / 0 | 0.919 |  |
| Festuca gigantea | 0.621 | 0 / 0 | 0.676 | 0 / 0 | 0.691 | 0 / 0 | 0.809 | forests |
| Festuca heterophylla | 0.788 | 0 / 0 | 0.908 | 0 / 0 | 0.886 | 0 / 0 | 0.966 | forests |
| Festuca norica | 0.906 | 0 / 0 | 0.909 | 0 / 0 | 0.894 | 0 / 0 | 0.98 | alpine habitats |
| Festuca picturata | 0.913 | 0 / 0 | 0.911 | 0 / 0 | 0.776 | 0 / 0 | 0.978 | alpine habitats |
| Festuca pratensis s. str. | 0.858 | 0 / 0 | 0.801 | 0 / 0 | 0.787 | 0 / 0 | 0.856 | grasslands |
| Festuca pseudodura | 0.98 | 0 / 0 | 0.945 | 0 / 0 | 0.939 | 0 / 0 | 0.983 | alpine habitats |
| Festuca pumila | 0.909 | 0 / 0 | 0.907 | 1 / 0 | 0.903 | 0 / 0 | 0.941 | alpine habitats |
| Festuca rubra agg. | 0.673 | 0 / 0 | 0.64 | 0 / 0 | 0.662 | 0 / 0 | 0.792 |  |
| Festuca rupicaprina | 0.888 | 0 / 0 | 0.876 | 0 / 0 | 0.767 | 0 / 0 | 0.935 | alpine habitats |
| Festuca rupicola | 0.828 | 0 / 0 | 0.845 | 0 / 0 | 0.794 | 0 / 0 | 0.918 | grasslands |
| Festuca versicolor | 0.894 | 0 / 0 | 0.88 | 0 / 0 | 0.853 | 0 / 0 | 0.94 | alpine habitats |
| Ficaria verna agg. | 0.649 | 0 / 0 | 0.656 | 0 / 0 | 0.601 | 0 / 0 | 0.864 |  |
| Filipendula ulmaria | 0.769 | 0 / 0 | 0.682 | 0 / 0 | 0.664 | 0 / 0 | 0.822 |  |
| Filipendula vulgaris | 0.87 | 0 / 0 | 0.878 | 0 / 0 | 0.86 | 0 / 0 | 0.939 | grasslands |
| Fragaria moschata | 0.602 | 0 / 1 | 0.556 | 0 / 0 | 0.563 | 1 / 0 | 0.864 |  |
| Fragaria vesca | 0.664 | 0 / 0 | 0.627 | 0 / 0 | 0.638 | 0 / 0 | 0.801 |  |
| Fragaria viridis | 0.771 | 0 / 0 | 0.792 | 0 / 0 | 0.712 | 0 / 0 | 0.899 |  |
| Frangula alnus | 0.609 | 0 / 0 | 0.619 | 1 / 0 | 0.575 | 0 / 0 | 0.773 |  |
| Fraxinus excelsior | 0.763 | 0 / 0 | 0.733 | 0 / 0 | 0.739 | 0 / 0 | 0.81 | forests |
| Galeobdolon flavidum | 0.555 | 0 / 0 | 0.759 | 0 / 0 | 0.694 | 1 / 0 | 0.882 |  |
| Galeobdolon montanum | 0.717 | 0 / 0 | 0.706 | 0 / 0 | 0.691 | 0 / 0 | 0.854 | forests |
| Galeopsis pubescens | 0.759 | 0 / 0 | 0.724 | 0 / 0 | 0.66 | 0 / 0 | 0.895 | forests |
| Galeopsis speciosa | 0.602 | 0 / 0 | 0.626 | 0 / 0 | 0.631 | 0 / 0 | 0.791 | forests |
| Galeopsis tetrahit | 0.646 | 0 / 0 | 0.619 | 0 / 0 | 0.656 | 0 / 1 | 0.877 |  |
| Galium anisophyllon | 0.798 | 0 / 0 | 0.781 | 0 / 0 | 0.81 | 0 / 0 | 0.861 | alpine habitats |
| Galium aparine s. str. | 0.858 | 0 / 0 | 0.852 | 0 / 0 | 0.849 | 0 / 0 | 0.872 |  |
| Galium boreale s. str. | 0.791 | 0 / 0 | 0.804 | 0 / 0 | 0.754 | 0 / 0 | 0.879 | grasslands |
| Galium elongatum | 0.971 | 0 / 0 | 0.973 | 0 / 0 | 0.973 | 0 / 0 | 0.993 | grasslands |
| Galium lucidum s. str. | 0.638 | 0 / 0 | 0.683 | 0 / 0 | 0.616 | 0 / 0 | 0.878 |  |
| Galium mollugo agg. | 0.647 | 0 / 0 | 0.613 | 0 / 0 | 0.626 | 0 / 0 | 0.775 |  |
| Galium noricum | 0.841 | 0 / 0 | 0.883 | 0 / 0 | 0.853 | 0 / 0 | 0.931 | alpine habitats |
| Galium odoratum | 0.749 | 0 / 0 | 0.763 | 0 / 0 | 0.759 | 0 / 0 | 0.848 | forests |
| Galium palustre agg. | 0.804 | 0 / 0 | 0.906 | 0 / 0 | 0.903 | 0 / 0 | 0.972 | grasslands |
| Galium palustre s. str. | 0.656 | 0 / 0 | 0.679 | 0 / 0 | 0.683 | 0 / 0 | 0.815 | grasslands |
| Galium pumilum | 0.816 | 0 / 0 | 0.823 | 0 / 0 | 0.745 | 0 / 0 | 0.933 | grasslands |
| Galium rotundifolium | 0.708 | 0 / 0 | 0.708 | 0 / 0 | 0.677 | 0 / 0 | 0.868 | forests |
| Galium sylvaticum | 0.724 | 0 / 0 | 0.738 | 0 / 0 | 0.73 | 0 / 0 | 0.853 | forests |
| Galium uliginosum | 0.645 | 0 / 0 | 0.739 | 0 / 0 | 0.742 | 0 / 0 | 0.814 | grasslands |
| Galium verum s. str. | 0.825 | 0 / 0 | 0.779 | 0 / 0 | 0.729 | 0 / 0 | 0.852 | grasslands |
| Gentiana acaulis | 0.9 | 0 / 0 | 0.942 | 0 / 0 | 0.935 | 0 / 0 | 0.972 | alpine habitats |
| Gentiana asclepiadea | 0.63 | 0 / 0 | 0.57 | 0 / 0 | 0.58 | 0 / 0 | 0.803 |  |
| Gentiana bavarica s. str. | 0.754 | 0 / 0 | 0.886 | 0 / 0 | 0.761 | 0 / 0 | 0.925 | alpine habitats |
| Gentiana clusii | 0.8 | 0 / 0 | 0.859 | 0 / 0 | 0.88 | 0 / 0 | 0.904 | alpine habitats |
| Gentiana nivalis | 0.718 | 0 / 0 | 0.89 | 0 / 0 | 0.813 | 1 / 0 | 0.934 | alpine habitats |
| Gentiana orbicularis | 0.834 | 0 / 0 | 0.909 | 0 / 0 | 0.533 | 0 / 0 | 0.922 | alpine habitats |
| Gentiana pannonica | 0.775 | 0 / 0 | 0.815 | 0 / 0 | 0.7 | 0 / 0 | 0.918 |  |
| Gentiana pneumonanthe | 0.854 | 0 / 0 | 0.907 | 0 / 0 | 0.867 | 0 / 0 | 0.968 | grasslands |
| Gentiana pumila | 0.886 | 0 / 0 | 0.892 | 0 / 0 | 0.839 | 0 / 0 | 0.952 | alpine habitats |
| Gentiana verna s. str. | 0.677 | 0 / 0 | 0.784 | 0 / 0 | 0.788 | 0 / 0 | 0.874 | alpine habitats |
| Gentianella aspera | 0.516 | 0 / 1 | 0.644 | 0 / 0 | 0.523 | 0 / 1 | 0.858 |  |
| Gentianella austriaca | 0.958 | 0 / 0 | 0.941 | 0 / 0 | 0.813 | 0 / 0 | 0.967 | alpine habitats |
| Gentianella germanica s. lat. | 0.813 | 0 / 0 | 0.856 | 0 / 0 | 0.861 | 0 / 0 | 0.937 | alpine habitats |
| Gentianopsis ciliata | 0.503 | 0 / 2 | 0.653 | 0 / 0 | 0.519 | 1 / 1 | 0.81 |  |
| Geranium dissectum | 0.714 | 0 / 0 | 0.538 | 0 / 0 | 0.725 | 0 / 0 | 0.871 | agricultural lands |
| Geranium phaeum | 0.7 | 0 / 0 | 0.705 | 0 / 0 | 0.696 | 0 / 0 | 0.874 |  |
| Geranium pusillum | 0.862 | 0 / 0 | 0.77 | 0 / 0 | 0.785 | 0 / 0 | 0.902 | agricultural lands |
| Geranium robertianum s. str. | 0.67 | 0 / 0 | 0.643 | 0 / 0 | 0.623 | 0 / 0 | 0.822 | forests |
| Geranium sylvaticum | 0.683 | 0 / 0 | 0.613 | 0 / 0 | 0.604 | 0 / 0 | 0.847 |  |
| Geum montanum | 0.87 | 0 / 0 | 0.885 | 0 / 0 | 0.849 | 0 / 0 | 0.928 | alpine habitats |
| Geum rivale | 0.579 | 0 / 0 | 0.533 | 0 / 0 | 0.503 | 1 / 0 | 0.805 |  |
| Geum urbanum | 0.726 | 0 / 0 | 0.706 | 0 / 0 | 0.71 | 0 / 0 | 0.81 |  |
| Glechoma hederacea | 0.72 | 0 / 0 | 0.669 | 0 / 0 | 0.653 | 0 / 0 | 0.834 |  |
| Globularia cordifolia | 0.78 | 0 / 0 | 0.708 | 0 / 0 | 0.714 | 1 / 0 | 0.908 |  |
| Globularia nudicaulis | 0.554 | 0 / 0 | 0.532 | 0 / 0 | NA | 1 / 2 | 0.858 |  |
| Glyceria fluitans agg. | NA | 0 / 3 | 0.566 | 0 / 2 | 0.583 | 1 / 1 | 0.647 |  |
| Gymnadenia conopsea s. lat. | 0.65 | 0 / 0 | 0.662 | 0 / 0 | 0.664 | 0 / 0 | 0.816 |  |
| Gymnocarpium dryopteris | 0.716 | 0 / 0 | 0.775 | 0 / 0 | 0.744 | 0 / 0 | 0.876 | forests |
| Gymnocarpium robertianum | 0.745 | 0 / 0 | 0.779 | 0 / 0 | 0.763 | 0 / 0 | 0.851 | forests |
| Hedera helix | 0.767 | 0 / 0 | 0.767 | 0 / 0 | 0.754 | 0 / 0 | 0.841 | forests |
| Hedysarum hedysaroides | 0.891 | 0 / 0 | 0.898 | 0 / 0 | 0.908 | 0 / 0 | 0.941 | alpine habitats |
| Helianthemum alpestre s. str. | 0.868 | 0 / 0 | 0.901 | 0 / 0 | 0.905 | 0 / 0 | 0.942 | alpine habitats |
| Helianthemum nummularium s. lat. | 0.781 | 0 / 0 | 0.774 | 0 / 0 | 0.755 | 0 / 0 | 0.853 |  |
| Helianthus annuus | 0.87 | 0 / 0 | 0.862 | 0 / 0 | 0.78 | 0 / 0 | 0.943 | agricultural lands |
| Helictotrichon parlatorei | 0.867 | 0 / 0 | 0.881 | 0 / 0 | 0.814 | 0 / 0 | 0.967 | alpine habitats |
| Heliosperma alpestre | 0.85 | 0 / 0 | 0.827 | 0 / 0 | 0.823 | 0 / 0 | 0.924 | alpine habitats |
| Heliosperma pusillum s. lat. | 0.748 | 0 / 0 | 0.822 | 0 / 0 | 0.755 | 0 / 0 | 0.894 | alpine habitats |
| Helleborus niger | 0.755 | 0 / 0 | 0.748 | 0 / 0 | 0.749 | 0 / 0 | 0.841 | forests |
| Hepatica nobilis | 0.701 | 0 / 0 | 0.718 | 0 / 0 | 0.688 | 0 / 0 | 0.831 | forests |
| Heracleum austriacum | 0.705 | 0 / 0 | 0.711 | 0 / 0 | 0.7 | 0 / 0 | 0.869 |  |
| Heracleum sphondylium | 0.628 | 0 / 0 | 0.554 | 0 / 0 | 0.535 | 0 / 0 | 0.78 |  |
| Hieracium alpinum s. lat. | 0.796 | 0 / 0 | 0.849 | 0 / 0 | 0.812 | 0 / 0 | 0.937 | alpine habitats |
| Hieracium bauhini | 0.584 | 0 / 0 | 0.837 | 0 / 0 | 0.863 | 1 / 1 | 0.938 | grasslands |
| Hieracium bifidum | 0.609 | 0 / 0 | 0.577 | 0 / 0 | 0.572 | 0 / 1 | 0.834 |  |
| Hieracium lachenalii | 0.543 | 0 / 1 | 0.593 | 0 / 0 | 0.6 | 0 / 1 | 0.858 |  |
| Hieracium lactucella | 0.529 | 0 / 2 | 0.688 | 0 / 0 | 0.632 | 1 / 1 | 0.854 | grasslands |
| Hieracium murorum | 0.632 | 0 / 0 | 0.616 | 0 / 0 | 0.606 | 0 / 0 | 0.786 | forests |
| Hieracium pilosella | 0.79 | 0 / 0 | 0.791 | 0 / 0 | 0.774 | 0 / 0 | 0.895 |  |
| Hieracium racemosum | 0.803 | 0 / 0 | 0.847 | 0 / 0 | 0.811 | 0 / 0 | 0.974 | forests |
| Hieracium villosum | 0.806 | 0 / 0 | 0.861 | 0 / 0 | 0.851 | 0 / 0 | 0.942 | alpine habitats |
| Hippocrepis comosa | 0.835 | 0 / 0 | 0.805 | 0 / 0 | 0.781 | 0 / 0 | 0.909 |  |
| Hippocrepis emerus | 0.582 | 0 / 0 | 0.704 | 0 / 0 | 0.606 | 0 / 0 | 0.883 | forests |
| Holcus lanatus | 0.803 | 0 / 0 | 0.783 | 0 / 0 | 0.733 | 0 / 0 | 0.853 | grasslands |
| Holcus mollis | 0.61 | 0 / 0 | 0.613 | 0 / 0 | 0.56 | 2 / 0 | 0.891 |  |
| Holosteum umbellatum | 0.827 | 0 / 0 | 0.794 | 0 / 0 | 0.632 | 0 / 0 | 0.955 |  |
| Homalotrichon pubescens | 0.849 | 0 / 0 | 0.829 | 0 / 0 | 0.819 | 0 / 0 | 0.888 | grasslands |
| Homogyne alpina | 0.64 | 0 / 0 | 0.686 | 0 / 0 | 0.687 | 0 / 0 | 0.804 |  |
| Homogyne discolor | 0.88 | 0 / 0 | 0.885 | 0 / 0 | 0.838 | 0 / 0 | 0.919 | alpine habitats |
| Hordelymus europaeus | 0.712 | 0 / 0 | 0.711 | 0 / 0 | 0.754 | 0 / 0 | 0.886 | forests |
| Hornungia alpina s. lat. | 0.889 | 0 / 0 | 0.937 | 0 / 0 | 0.941 | 0 / 0 | 0.956 | alpine habitats |
| Humulus lupulus | 0.772 | 0 / 0 | 0.76 | 0 / 0 | 0.71 | 0 / 0 | 0.878 |  |
| Huperzia selago | 0.599 | 0 / 0 | 0.617 | 0 / 0 | 0.659 | 1 / 0 | 0.844 |  |
| Hylotelephium telephium agg. | 0.595 | 0 / 1 | 0.542 | 0 / 0 | NA | 1 / 2 | 0.865 |  |
| Hypericum hirsutum | 0.555 | 0 / 0 | 0.635 | 0 / 0 | 0.559 | 0 / 0 | 0.813 | forests |
| Hypericum maculatum s. str. | 0.672 | 0 / 0 | 0.642 | 0 / 0 | 0.675 | 0 / 0 | 0.801 |  |
| Hypericum perforatum | 0.51 | 0 / 1 | 0.5 | 0 / 1 | NA | 0 / 3 | 0.883 |  |
| Hypericum tetrapterum | NA | 0 / 3 | 0.519 | 0 / 1 | NA | 0 / 3 | 0.713 |  |
| Hypochaeris radicata | 0.801 | 0 / 0 | 0.773 | 0 / 0 | 0.782 | 0 / 0 | 0.912 | grasslands |
| Hypochaeris uniflora | 0.968 | 0 / 0 | 0.966 | 0 / 0 | 0.952 | 0 / 0 | 0.995 | alpine habitats |
| Impatiens glandulifera | 0.796 | 0 / 0 | 0.757 | 0 / 0 | 0.638 | 1 / 0 | 0.936 | forests |
| Impatiens noli-tangere | 0.669 | 0 / 0 | 0.666 | 0 / 0 | 0.662 | 1 / 0 | 0.807 | forests |
| Impatiens parviflora | 0.652 | 0 / 0 | 0.642 | 0 / 0 | 0.624 | 0 / 0 | 0.829 |  |
| Inula britannica | 0.832 | 0 / 0 | 0.932 | 0 / 0 | 0.916 | 0 / 0 | 0.981 | grasslands |
| Inula conyzae | NA | 0 / 3 | 0.566 | 0 / 1 | 0.581 | 0 / 2 | 0.797 |  |
| Inula salicina | 0.769 | 0 / 0 | 0.767 | 0 / 0 | 0.769 | 0 / 0 | 0.901 | grasslands |
| Iris pseudacorus | 0.877 | 0 / 0 | 0.916 | 0 / 0 | 0.882 | 0 / 0 | 0.945 |  |
| Juglans regia | 0.675 | 0 / 0 | 0.589 | 0 / 0 | 0.53 | 0 / 0 | 0.848 |  |
| Juncus alpinoarticulatus | 0.846 | 0 / 0 | 0.873 | 0 / 0 | 0.861 | 0 / 0 | 0.893 | grasslands |
| Juncus articulatus | 0.612 | 0 / 0 | 0.714 | 0 / 0 | 0.578 | 0 / 0 | 0.82 | grasslands |
| Juncus effusus | 0.613 | 0 / 0 | 0.563 | 0 / 0 | 0.536 | 0 / 0 | 0.769 |  |
| Juncus filiformis | 0.648 | 0 / 0 | 0.756 | 0 / 0 | 0.708 | 0 / 0 | 0.856 | grasslands |
| Juncus inflexus | 0.667 | 0 / 0 | 0.709 | 0 / 0 | 0.644 | 0 / 0 | 0.839 | grasslands |
| Juncus jacquinii | 0.858 | 0 / 0 | 0.929 | 0 / 0 | 0.932 | 0 / 0 | 0.959 | alpine habitats |
| Juncus monanthos | 0.778 | 0 / 0 | 0.833 | 0 / 0 | 0.861 | 0 / 0 | 0.919 | alpine habitats |
| Juncus trifidus | 0.948 | 0 / 0 | 0.925 | 0 / 0 | 0.908 | 0 / 0 | 0.967 | alpine habitats |
| Juniperus communis | NA | 0 / 3 | NA | 0 / 3 | NA | 0 / 3 | NA |  |
| Kernera saxatilis | 0.574 | 0 / 0 | 0.703 | 0 / 0 | 0.567 | 1 / 0 | 0.886 |  |
| Knautia arvensis s. str. | 0.811 | 0 / 0 | 0.725 | 0 / 0 | 0.753 | 1 / 0 | 0.861 | grasslands |
| Knautia drymeia s. lat. | 0.822 | 0 / 0 | 0.87 | 0 / 0 | 0.83 | 0 / 0 | 0.934 |  |
| Knautia maxima | 0.641 | 0 / 0 | 0.615 | 0 / 0 | 0.615 | 0 / 0 | 0.799 |  |
| Koeleria macrantha | 0.991 | 0 / 0 | 0.994 | 0 / 0 | 0.993 | 0 / 0 | 0.994 | grasslands |
| Koeleria pyramidata | 0.786 | 0 / 0 | 0.843 | 0 / 0 | 0.827 | 0 / 0 | 0.928 | grasslands |
| Lactuca muralis | 0.703 | 0 / 0 | 0.721 | 0 / 0 | 0.701 | 0 / 0 | 0.832 | forests |
| Lactuca serriola | 0.848 | 0 / 0 | 0.812 | 0 / 0 | 0.762 | 0 / 0 | 0.927 | agricultural lands |
| Lamium amplexicaule | 0.961 | 0 / 0 | 0.876 | 0 / 0 | 0.868 | 0 / 0 | 0.953 | agricultural lands |
| Lamium maculatum | 0.699 | 0 / 0 | 0.653 | 0 / 0 | 0.635 | 0 / 0 | 0.809 |  |
| Lamium purpureum | 0.814 | 0 / 0 | 0.744 | 0 / 0 | 0.768 | 0 / 0 | 0.891 | agricultural lands |
| Lapsana communis | 0.755 | 0 / 0 | 0.823 | 0 / 0 | 0.601 | 0 / 0 | 0.875 |  |
| Larix decidua | 0.753 | 0 / 0 | 0.742 | 0 / 0 | 0.745 | 0 / 0 | 0.824 | forests |
| Laserpitium latifolium | 0.646 | 0 / 0 | 0.703 | 0 / 0 | 0.649 | 0 / 0 | 0.82 |  |
| Lathyrus pratensis | 0.736 | 0 / 0 | 0.67 | 0 / 0 | 0.677 | 0 / 0 | 0.79 | grasslands |
| Lathyrus tuberosus | 0.884 | 0 / 0 | 0.914 | 0 / 0 | 0.809 | 1 / 0 | 0.954 | agricultural lands |
| Lathyrus vernus | 0.799 | 0 / 0 | 0.906 | 0 / 0 | 0.912 | 0 / 0 | 0.972 | forests |
| Leontodon hispidus | 0.671 | 0 / 0 | 0.632 | 0 / 0 | 0.603 | 0 / 0 | 0.767 |  |
| Leontodon incanus | 0.581 | 0 / 1 | 0.675 | 0 / 0 | 0.584 | 0 / 0 | 0.833 |  |
| Lepidium draba | 0.957 | 0 / 0 | 0.849 | 0 / 0 | 0.505 | 0 / 0 | 0.959 | agricultural lands |
| Leucanthemopsis alpina | 0.836 | 0 / 0 | 0.966 | 0 / 0 | 0.932 | 0 / 0 | 0.981 | alpine habitats |
| Leucanthemum atratum s. str. | 0.88 | 0 / 0 | 0.871 | 0 / 0 | 0.815 | 0 / 0 | 0.92 | alpine habitats |
| Leucanthemum ircutianum | 0.809 | 0 / 0 | 0.766 | 0 / 0 | 0.78 | 0 / 0 | 0.884 | grasslands |
| Leucanthemum vulgare agg. | 0.766 | 0 / 0 | 0.753 | 0 / 0 | 0.693 | 0 / 0 | 0.887 | grasslands |
| Leucanthemum vulgare s. str. | 0.845 | 0 / 0 | 0.824 | 0 / 0 | 0.785 | 0 / 0 | 0.885 | grasslands |
| Ligustrum vulgare | 0.749 | 0 / 0 | 0.727 | 0 / 0 | 0.735 | 0 / 0 | 0.836 |  |
| Lilium martagon | 0.617 | 0 / 0 | 0.682 | 0 / 0 | 0.636 | 0 / 0 | 0.816 |  |
| Linaria alpina | 0.895 | 0 / 0 | 0.896 | 0 / 0 | 0.813 | 1 / 0 | 0.954 | alpine habitats |
| Linum alpinum | 0.776 | 0 / 0 | 0.798 | 0 / 0 | 0.604 | 0 / 0 | 0.925 | alpine habitats |
| Linum catharticum | 0.66 | 0 / 0 | 0.652 | 0 / 0 | 0.646 | 0 / 0 | 0.776 | grasslands |
| Listera ovata | 0.519 | 0 / 0 | 0.523 | 0 / 0 | 0.53 | 0 / 1 | 0.792 |  |
| Loiseleuria procumbens | 0.828 | 0 / 0 | 0.82 | 0 / 0 | 0.825 | 0 / 0 | 0.948 | alpine habitats |
| Lolium multiflorum | 0.959 | 0 / 0 | 0.894 | 0 / 0 | 0.874 | 0 / 0 | 0.941 |  |
| Lolium perenne | 0.83 | 0 / 0 | 0.79 | 0 / 0 | 0.771 | 0 / 0 | 0.857 |  |
| Lonicera alpigena | 0.714 | 0 / 0 | 0.739 | 0 / 0 | 0.696 | 0 / 0 | 0.86 | forests |
| Lonicera nigra | 0.546 | 0 / 0 | 0.682 | 0 / 0 | 0.655 | 0 / 0 | 0.881 | forests |
| Lonicera xylosteum | 0.766 | 0 / 0 | 0.732 | 0 / 0 | 0.73 | 0 / 0 | 0.828 | forests |
| Lotus corniculatus s. str. | 0.702 | 0 / 0 | 0.617 | 0 / 0 | 0.612 | 0 / 0 | 0.772 |  |
| Lunaria rediviva | 0.569 | 0 / 0 | 0.628 | 0 / 0 | 0.583 | 1 / 0 | 0.873 | forests |
| Luzula alpinopilosa | 0.886 | 0 / 0 | 0.972 | 0 / 0 | 0.91 | 0 / 0 | 0.984 | alpine habitats |
| Luzula campestris agg. | 0.666 | 0 / 0 | 0.697 | 0 / 0 | 0.574 | 0 / 0 | 0.824 | grasslands |
| Luzula glabrata | 0.899 | 0 / 0 | 0.926 | 0 / 0 | 0.851 | 0 / 0 | 0.949 | alpine habitats |
| Luzula luzulina | 0.647 | 0 / 0 | 0.76 | 0 / 0 | 0.632 | 1 / 0 | 0.924 |  |
| Luzula luzuloides | 0.669 | 0 / 0 | 0.658 | 0 / 0 | 0.599 | 0 / 0 | 0.806 | forests |
| Luzula multiflora s. lat. | 0.695 | 0 / 0 | 0.693 | 0 / 0 | 0.641 | 0 / 0 | 0.798 |  |
| Luzula pilosa | 0.618 | 0 / 0 | 0.61 | 0 / 0 | 0.58 | 0 / 0 | 0.827 | forests |
| Luzula spicata | 0.887 | 0 / 0 | 0.967 | 0 / 0 | 0.947 | 0 / 0 | 0.953 | alpine habitats |
| Luzula sylvatica s. lat. | 0.688 | 0 / 0 | 0.737 | 0 / 0 | 0.756 | 0 / 0 | 0.819 |  |
| Lychnis flos-cuculi | 0.745 | 0 / 0 | 0.707 | 0 / 0 | 0.652 | 0 / 0 | 0.802 | grasslands |
| Lycopodium annotinum | 0.747 | 0 / 0 | 0.807 | 0 / 0 | 0.775 | 0 / 0 | 0.877 |  |
| Lycopus europaeus | 0.616 | 0 / 0 | 0.669 | 0 / 0 | 0.616 | 0 / 0 | 0.854 |  |
| Lysimachia nemorum | 0.655 | 0 / 0 | 0.564 | 1 / 0 | 0.615 | 0 / 0 | 0.812 |  |
| Lysimachia nummularia | 0.637 | 0 / 0 | 0.627 | 0 / 0 | 0.628 | 0 / 0 | 0.815 |  |
| Lysimachia vulgaris | 0.774 | 0 / 0 | 0.728 | 0 / 0 | 0.71 | 0 / 0 | 0.858 | grasslands |
| Lythrum salicaria | 0.74 | 0 / 0 | 0.703 | 0 / 0 | 0.719 | 0 / 0 | 0.861 | grasslands |
| Maianthemum bifolium | 0.682 | 0 / 0 | 0.683 | 0 / 0 | 0.703 | 0 / 0 | 0.824 | forests |
| Malus domestica | 0.554 | 0 / 2 | NA | 0 / 3 | NA | 0 / 3 | 0.72 |  |
| Matricaria chamomilla | 0.953 | 0 / 0 | 0.955 | 0 / 0 | 0.893 | 0 / 0 | 0.976 | agricultural lands |
| Matricaria discoidea | 0.812 | 0 / 0 | 0.749 | 0 / 0 | 0.728 | 0 / 0 | 0.906 |  |
| Medicago falcata | 0.788 | 0 / 0 | 0.79 | 0 / 0 | 0.709 | 0 / 0 | 0.919 | grasslands |
| Medicago lupulina | 0.793 | 0 / 0 | 0.782 | 0 / 0 | 0.68 | 0 / 0 | 0.827 |  |
| Medicago sativa agg. | 0.824 | 0 / 0 | 0.8 | 0 / 0 | 0.707 | 0 / 0 | 0.895 |  |
| Melampyrum pratense | NA | 0 / 3 | 0.582 | 0 / 0 | 0.595 | 0 / 0 | 0.711 |  |
| Melampyrum sylvaticum s. str. | 0.698 | 0 / 0 | 0.689 | 0 / 0 | 0.702 | 0 / 0 | 0.829 |  |
| Melica nutans | 0.642 | 0 / 0 | 0.66 | 0 / 0 | 0.657 | 0 / 0 | 0.818 | forests |
| Melittis melissophyllum | 0.895 | 0 / 0 | 0.844 | 0 / 0 | 0.842 | 0 / 0 | 0.938 | forests |
| Mentha aquatica | 0.773 | 0 / 0 | 0.761 | 0 / 0 | 0.784 | 0 / 0 | 0.887 | grasslands |
| Mentha arvensis | 0.626 | 0 / 0 | 0.671 | 0 / 0 | 0.646 | 0 / 0 | 0.835 | grasslands |
| Mentha longifolia | 0.612 | 0 / 0 | 0.561 | 0 / 0 | 0.529 | 0 / 1 | 0.781 |  |
| Menyanthes trifoliata | 0.805 | 0 / 0 | 0.844 | 0 / 0 | 0.853 | 0 / 0 | 0.873 | grasslands |
| Mercurialis annua | 0.968 | 0 / 0 | 0.845 | 0 / 0 | 0.773 | 0 / 0 | 0.98 | agricultural lands |
| Mercurialis perennis s. str. | 0.729 | 0 / 0 | 0.728 | 0 / 0 | 0.738 | 0 / 0 | 0.823 | forests |
| Meum athamanticum | 0.804 | 0 / 0 | 0.823 | 0 / 0 | 0.768 | 0 / 0 | 0.959 | alpine habitats |
| Microthlaspi perfoliatum | 0.926 | 0 / 0 | 0.845 | 0 / 0 | 0.707 | 0 / 0 | 0.951 |  |
| Milium effusum | 0.704 | 0 / 0 | 0.725 | 0 / 0 | 0.662 | 0 / 0 | 0.859 | forests |
| Minuartia austriaca | 0.865 | 0 / 0 | 0.873 | 0 / 0 | 0.839 | 1 / 0 | 0.972 | alpine habitats |
| Minuartia gerardii | 0.798 | 0 / 0 | 0.862 | 0 / 0 | 0.839 | 0 / 0 | 0.921 | alpine habitats |
| Minuartia sedoides | 0.905 | 0 / 0 | 0.902 | 0 / 0 | 0.903 | 0 / 0 | 0.949 | alpine habitats |
| Moehringia ciliata | 0.829 | 0 / 0 | 0.83 | 0 / 0 | 0.818 | 0 / 0 | 0.963 | alpine habitats |
| Moehringia muscosa | 0.717 | 0 / 0 | 0.784 | 0 / 0 | 0.765 | 1 / 0 | 0.873 | forests |
| Moehringia trinervia | 0.611 | 0 / 2 | 0.547 | 0 / 0 | 0.631 | 0 / 1 | 0.799 | forests |
| Molinia arundinacea | NA | 0 / 3 | 0.569 | 0 / 1 | 0.565 | 0 / 2 | 0.685 |  |
| Molinia caerulea agg. | 0.547 | 0 / 0 | 0.525 | 0 / 1 | 0.506 | 0 / 2 | 0.887 |  |
| Molinia caerulea | 0.712 | 0 / 0 | 0.661 | 0 / 0 | 0.668 | 0 / 0 | 0.795 | grasslands |
| Mutellina adonidifolia | 0.853 | 0 / 0 | 0.868 | 0 / 0 | 0.853 | 0 / 0 | 0.921 | alpine habitats |
| Myosotis alpestris | 0.849 | 0 / 0 | 0.873 | 0 / 0 | 0.866 | 0 / 0 | 0.909 | alpine habitats |
| Myosotis arvensis | 0.784 | 0 / 0 | 0.763 | 0 / 0 | 0.742 | 0 / 0 | 0.869 | agricultural lands |
| Myosotis palustris agg. | 0.587 | 0 / 0 | 0.501 | 0 / 0 | 0.563 | 0 / 2 | 0.791 |  |
| Myosotis sylvatica s. str. | 0.677 | 0 / 0 | 0.625 | 0 / 0 | 0.585 | 0 / 0 | 0.832 |  |
| Narcissus radiiflorus | 0.795 | 0 / 0 | 0.811 | 0 / 0 | 0.722 | 0 / 0 | 0.941 | grasslands |
| Nardus stricta | 0.642 | 0 / 0 | 0.626 | 0 / 0 | 0.634 | 0 / 0 | 0.772 |  |
| Neottia nidus-avis | 0.674 | 0 / 0 | 0.781 | 0 / 0 | 0.769 | 0 / 0 | 0.855 | forests |
| Nigritella nigra agg. | 0.695 | 0 / 0 | 0.834 | 0 / 0 | 0.73 | 0 / 0 | 0.928 | alpine habitats |
| Noccaea crantzii | 0.851 | 0 / 0 | 0.886 | 0 / 0 | 0.886 | 0 / 0 | 0.929 | alpine habitats |
| Onobrychis viciifolia agg. | 0.864 | 0 / 0 | 0.79 | 0 / 0 | 0.691 | 0 / 0 | 0.943 | grasslands |
| Ononis spinosa | 0.696 | 0 / 0 | 0.759 | 0 / 0 | 0.671 | 0 / 0 | 0.894 | grasslands |
| Orchis mascula s. lat. | 0.764 | 0 / 0 | 0.72 | 0 / 0 | 0.679 | 0 / 0 | 0.86 | grasslands |
| Oreochloa disticha | 0.942 | 0 / 0 | 0.859 | 0 / 0 | 0.894 | 0 / 0 | 0.978 | alpine habitats |
| Origanum vulgare s. str. | 0.654 | 0 / 0 | 0.591 | 0 / 0 | 0.602 | 0 / 0 | 0.789 |  |
| Oxalis acetosella | 0.762 | 0 / 0 | 0.764 | 0 / 0 | 0.767 | 0 / 0 | 0.836 | forests |
| Oxytropis campestris | 0.865 | 0 / 0 | 0.921 | 0 / 0 | 0.935 | 0 / 0 | 0.967 | alpine habitats |
| Oxytropis montana agg. | 0.746 | 0 / 0 | 0.7 | 0 / 0 | 0.689 | 0 / 0 | 0.936 | alpine habitats |
| Pachypleurum mutellinoides | 0.907 | 0 / 0 | 0.868 | 0 / 0 | 0.87 | 0 / 0 | 0.977 | alpine habitats |
| Papaver rhoeas | 0.904 | 0 / 0 | 0.908 | 0 / 0 | 0.905 | 0 / 0 | 0.934 | agricultural lands |
| Paris quadrifolia | 0.696 | 0 / 0 | 0.708 | 0 / 0 | 0.712 | 0 / 0 | 0.825 | forests |
| Parnassia palustris | 0.707 | 0 / 0 | 0.697 | 0 / 0 | 0.645 | 0 / 0 | 0.803 |  |
| Pastinaca sativa | 0.774 | 0 / 0 | 0.719 | 0 / 0 | 0.669 | 0 / 0 | 0.87 |  |
| Pedicularis foliosa | 0.935 | 0 / 0 | 0.961 | 0 / 0 | 0.919 | 0 / 0 | 0.981 | alpine habitats |
| Pedicularis palustris | 0.708 | 0 / 0 | 0.851 | 0 / 0 | 0.75 | 1 / 0 | 0.899 | grasslands |
| Pedicularis rostratocapitata | 0.884 | 0 / 0 | 0.91 | 0 / 0 | 0.905 | 0 / 0 | 0.941 | alpine habitats |
| Pedicularis verticillata | 0.817 | 0 / 0 | 0.874 | 0 / 0 | 0.868 | 0 / 0 | 0.922 | alpine habitats |
| Persicaria amphibia | 0.782 | 0 / 0 | 0.764 | 0 / 0 | 0.806 | 0 / 0 | 0.962 | grasslands |
| Persicaria bistorta | 0.679 | 0 / 1 | 0.586 | 0 / 0 | 0.539 | 2 / 0 | 0.837 |  |
| Persicaria hydropiper | 0.532 | 0 / 0 | 0.566 | 0 / 0 | 0.589 | 0 / 1 | 0.858 |  |
| Persicaria lapathifolia | 0.826 | 0 / 0 | 0.786 | 0 / 0 | 0.657 | 0 / 0 | 0.942 | agricultural lands |
| Persicaria maculosa | 0.896 | 0 / 0 | 0.896 | 0 / 0 | 0.74 | 0 / 0 | 0.943 | agricultural lands |
| Persicaria vivipara | 0.876 | 0 / 0 | 0.868 | 0 / 0 | 0.86 | 0 / 0 | 0.912 | alpine habitats |
| Petasites albus | 0.691 | 0 / 0 | 0.698 | 0 / 0 | 0.692 | 0 / 0 | 0.834 | forests |
| Petasites hybridus | 0.613 | 0 / 0 | 0.575 | 0 / 0 | 0.616 | 0 / 0 | 0.823 |  |
| Petasites paradoxus | 0.61 | 0 / 0 | 0.743 | 0 / 0 | 0.653 | 0 / 0 | 0.849 |  |
| Peucedanum oreoselinum | 0.719 | 0 / 0 | 0.745 | 0 / 0 | 0.614 | 0 / 0 | 0.925 |  |
| Peucedanum ostruthium | 0.744 | 0 / 0 | 0.747 | 0 / 0 | 0.706 | 0 / 0 | 0.892 |  |
| Peucedanum palustre | 0.725 | 0 / 0 | 0.853 | 0 / 0 | 0.842 | 0 / 0 | 0.929 | grasslands |
| Phacelia tanacetifolia | 0.845 | 0 / 0 | 0.858 | 0 / 0 | 0.805 | 0 / 0 | 0.914 | agricultural lands |
| Phalaris arundinacea | 0.807 | 0 / 0 | 0.81 | 0 / 0 | 0.742 | 0 / 0 | 0.897 |  |
| Phegopteris connectilis | 0.628 | 0 / 0 | 0.776 | 0 / 0 | 0.646 | 1 / 0 | 0.875 | forests |
| Phleum alpinum agg. | 0.752 | 0 / 0 | 0.833 | 0 / 0 | 0.769 | 0 / 0 | 0.906 |  |
| Phleum hirsutum | 0.737 | 0 / 0 | 0.804 | 0 / 0 | 0.699 | 0 / 0 | 0.932 | alpine habitats |
| Phleum phleoides | 0.769 | 0 / 0 | 0.967 | 0 / 0 | 0.787 | 0 / 0 | 0.987 | grasslands |
| Phleum pratense | 0.717 | 0 / 0 | 0.611 | 0 / 0 | 0.533 | 0 / 1 | 0.844 |  |
| Phragmites australis | 0.793 | 0 / 0 | 0.701 | 0 / 0 | 0.68 | 0 / 0 | 0.841 | grasslands |
| Phyteuma confusum | 0.858 | 0 / 0 | 0.92 | 0 / 0 | 0.68 | 1 / 0 | 0.949 | alpine habitats |
| Phyteuma globulariifolium | 0.989 | 0 / 0 | 0.956 | 0 / 0 | 0.982 | 0 / 0 | 0.985 | alpine habitats |
| Phyteuma orbiculare | 0.746 | 0 / 0 | 0.751 | 0 / 0 | 0.703 | 0 / 0 | 0.809 |  |
| Phyteuma spicatum | 0.72 | 0 / 0 | 0.709 | 0 / 0 | 0.689 | 0 / 0 | 0.83 | forests |
| Picea abies | 0.783 | 0 / 0 | 0.761 | 0 / 0 | 0.761 | 0 / 0 | 0.85 | forests |
| Picris hieracioides s. lat. | 0.846 | 0 / 0 | 0.822 | 0 / 0 | 0.683 | 0 / 0 | 0.923 |  |
| Pimpinella major | 0.545 | 0 / 0 | 0.522 | 0 / 0 | 0.541 | 0 / 0 | 0.796 |  |
| Pimpinella saxifraga agg. | 0.744 | 0 / 0 | 0.673 | 0 / 0 | 0.668 | 0 / 0 | 0.844 |  |
| Pinguicula alpina | 0.665 | 0 / 0 | 0.701 | 0 / 0 | 0.684 | 0 / 0 | 0.867 |  |
| Pinguicula vulgaris | 0.716 | 0 / 0 | 0.791 | 0 / 0 | 0.804 | 2 / 0 | 0.867 | grasslands |
| Pinus mugo s. str. | 0.653 | 0 / 0 | 0.749 | 0 / 0 | 0.704 | 0 / 0 | 0.812 |  |
| Pinus sylvestris | 0.608 | 0 / 0 | 0.642 | 0 / 0 | 0.594 | 0 / 0 | 0.789 | forests |
| Pisum sativum | 0.867 | 0 / 0 | 0.849 | 0 / 0 | 0.75 | 0 / 0 | 0.937 | agricultural lands |
| Plantago lanceolata | 0.814 | 0 / 0 | 0.757 | 0 / 0 | 0.756 | 0 / 0 | 0.841 | grasslands |
| Plantago major s. lat. | 0.709 | 0 / 0 | 0.583 | 0 / 0 | 0.561 | 0 / 0 | 0.81 |  |
| Plantago media | 0.81 | 0 / 0 | 0.796 | 0 / 0 | 0.768 | 1 / 0 | 0.876 | grasslands |
| Platanthera bifolia | NA | 0 / 3 | 0.511 | 0 / 0 | NA | 1 / 2 | 0.731 |  |
| Poa alpina | 0.86 | 0 / 0 | 0.863 | 0 / 0 | 0.853 | 0 / 0 | 0.898 | alpine habitats |
| Poa angustifolia | 0.813 | 0 / 0 | 0.786 | 0 / 0 | 0.776 | 0 / 0 | 0.885 |  |
| Poa annua | 0.803 | 0 / 0 | 0.768 | 0 / 0 | 0.721 | 0 / 0 | 0.875 |  |
| Poa compressa | 0.836 | 0 / 0 | 0.74 | 0 / 0 | 0.607 | 0 / 0 | 0.918 |  |
| Poa minor | 0.898 | 0 / 0 | 0.926 | 0 / 0 | 0.904 | 0 / 0 | 0.98 | alpine habitats |
| Poa nemoralis | 0.732 | 0 / 0 | 0.679 | 0 / 0 | 0.659 | 0 / 0 | 0.829 | forests |
| Poa palustris | 0.692 | 0 / 0 | 0.743 | 0 / 0 | 0.69 | 1 / 0 | 0.859 |  |
| Poa pratensis agg. | 0.955 | 0 / 0 | 0.949 | 0 / 0 | 0.902 | 0 / 0 | 0.971 | grasslands |
| Poa pratensis | 0.724 | 0 / 0 | 0.699 | 0 / 0 | 0.685 | 0 / 0 | 0.806 |  |
| Poa trivialis subsp. trivialis | 0.707 | 0 / 0 | 0.63 | 0 / 0 | 0.641 | 0 / 0 | 0.799 |  |
| Polygala amara | 0.666 | 0 / 0 | 0.62 | 0 / 0 | 0.666 | 1 / 0 | 0.878 |  |
| Polygala amarella | 0.789 | 0 / 0 | 0.834 | 0 / 0 | 0.744 | 0 / 0 | 0.895 | grasslands |
| Polygala chamaebuxus | 0.644 | 0 / 0 | 0.666 | 0 / 0 | 0.674 | 0 / 1 | 0.811 |  |
| Polygala comosa | 0.695 | 0 / 0 | 0.794 | 0 / 0 | 0.705 | 0 / 0 | 0.889 | grasslands |
| Polygala vulgaris | 0.783 | 0 / 0 | 0.876 | 0 / 0 | 0.845 | 0 / 0 | 0.904 | grasslands |
| Polygonatum multiflorum | 0.762 | 0 / 0 | 0.742 | 0 / 0 | 0.732 | 0 / 0 | 0.833 | forests |
| Polygonatum odoratum | 0.692 | 0 / 0 | 0.669 | 0 / 0 | 0.635 | 0 / 0 | 0.835 |  |
| Polygonatum verticillatum | 0.732 | 0 / 0 | 0.757 | 0 / 0 | 0.78 | 0 / 0 | 0.846 | forests |
| Polygonum aviculare s. lat. | 0.889 | 0 / 0 | 0.844 | 0 / 0 | 0.821 | 0 / 0 | 0.909 | agricultural lands |
| Polypodium vulgare s. str. | 0.558 | 0 / 1 | 0.588 | 0 / 0 | 0.595 | 1 / 0 | 0.802 | forests |
| Polystichum aculeatum s. str. | 0.751 | 0 / 0 | 0.766 | 0 / 0 | 0.773 | 0 / 0 | 0.859 | forests |
| Polystichum lonchitis | 0.668 | 0 / 0 | 0.748 | 0 / 0 | 0.73 | 0 / 0 | 0.876 |  |
| Populus tremula | 0.568 | 0 / 0 | 0.614 | 0 / 0 | 0.549 | 0 / 1 | 0.816 |  |
| Potentilla anserina | 0.742 | 0 / 0 | 0.718 | 0 / 0 | 0.589 | 1 / 0 | 0.894 |  |
| Potentilla aurea | 0.905 | 0 / 0 | 0.892 | 0 / 0 | 0.898 | 0 / 0 | 0.923 | alpine habitats |
| Potentilla clusiana | 0.827 | 0 / 0 | 0.866 | 0 / 0 | 0.814 | 0 / 0 | 0.933 | alpine habitats |
| Potentilla crantzii | 0.881 | 0 / 0 | 0.876 | 0 / 0 | 0.805 | 0 / 0 | 0.938 | alpine habitats |
| Potentilla erecta | 0.69 | 0 / 0 | 0.681 | 0 / 0 | 0.668 | 0 / 0 | 0.775 | grasslands |
| Potentilla reptans | 0.825 | 0 / 0 | 0.75 | 0 / 0 | 0.739 | 0 / 0 | 0.89 | grasslands |
| Potentilla sterilis | 0.594 | 0 / 0 | 0.703 | 0 / 0 | 0.586 | 0 / 1 | 0.862 |  |
| Potentilla verna agg. | 0.943 | 0 / 0 | 0.898 | 0 / 0 | 0.885 | 0 / 0 | 0.958 | grasslands |
| Prenanthes purpurea | 0.742 | 0 / 0 | 0.739 | 0 / 0 | 0.735 | 0 / 0 | 0.855 | forests |
| Primula auricula | 0.848 | 0 / 0 | 0.889 | 0 / 0 | 0.867 | 0 / 0 | 0.932 | alpine habitats |
| Primula clusiana | 0.861 | 0 / 0 | 0.881 | 0 / 0 | 0.883 | 0 / 0 | 0.931 | alpine habitats |
| Primula elatior s. str. | 0.619 | 0 / 0 | 0.627 | 0 / 0 | 0.617 | 0 / 0 | 0.795 |  |
| Primula farinosa | 0.835 | 0 / 0 | 0.851 | 0 / 0 | 0.818 | 0 / 0 | 0.891 | grasslands |
| Primula minima | 0.91 | 0 / 0 | 0.933 | 0 / 0 | 0.964 | 0 / 0 | 0.972 | alpine habitats |
| Primula veris subsp. veris | 0.794 | 0 / 0 | 0.783 | 0 / 0 | 0.773 | 0 / 0 | 0.891 | grasslands |
| Primula vulgaris | 0.929 | 0 / 0 | 0.896 | 0 / 0 | 0.848 | 0 / 0 | 0.965 | forests |
| Prunella grandiflora | 0.741 | 0 / 0 | 0.814 | 0 / 0 | 0.75 | 0 / 0 | 0.917 | grasslands |
| Prunella vulgaris | 0.65 | 0 / 0 | 0.597 | 0 / 0 | 0.568 | 0 / 1 | 0.775 | grasslands |
| Prunus avium | 0.753 | 0 / 0 | 0.713 | 0 / 0 | 0.684 | 0 / 0 | 0.825 |  |
| Prunus padus | 0.754 | 0 / 0 | 0.766 | 0 / 0 | 0.683 | 0 / 0 | 0.877 |  |
| Pteridium aquilinum | 0.672 | 0 / 0 | 0.71 | 0 / 0 | 0.679 | 0 / 0 | 0.82 | forests |
| Pulmonaria kerneri | 0.653 | 0 / 1 | 0.574 | 0 / 0 | 0.539 | 0 / 1 | 0.845 | forests |
| Pulmonaria officinalis s. str. | 0.664 | 0 / 0 | 0.687 | 0 / 0 | 0.689 | 0 / 0 | 0.824 | forests |
| Pulsatilla alpina s. lat. | 0.798 | 0 / 0 | 0.861 | 0 / 0 | 0.726 | 0 / 0 | 0.953 | alpine habitats |
| Pyrus pyraster | 0.732 | 0 / 0 | 0.776 | 0 / 0 | 0.78 | 0 / 0 | 0.942 | forests |
| Quercus petraea s. lat. | 0.929 | 0 / 0 | 0.921 | 0 / 0 | 0.89 | 0 / 0 | 0.959 | forests |
| Quercus robur | 0.676 | 0 / 0 | 0.663 | 0 / 0 | 0.61 | 0 / 0 | 0.787 |  |
| Ranunculus aconitifolius s. str. | NA | 0 / 3 | 0.515 | 0 / 0 | NA | 0 / 3 | 0.79 |  |
| Ranunculus acris s. lat. | 0.72 | 0 / 0 | 0.685 | 0 / 0 | 0.629 | 0 / 0 | 0.784 | grasslands |
| Ranunculus alpestris | 0.894 | 0 / 0 | 0.903 | 0 / 0 | 0.885 | 0 / 0 | 0.928 | alpine habitats |
| Ranunculus auricomus agg. | 0.821 | 0 / 0 | 0.797 | 0 / 0 | 0.735 | 1 / 0 | 0.926 |  |
| Ranunculus bulbosus | 0.859 | 0 / 0 | 0.844 | 0 / 0 | 0.821 | 0 / 0 | 0.926 | grasslands |
| Ranunculus hybridus | 0.7 | 0 / 0 | 0.859 | 0 / 0 | 0.69 | 0 / 0 | 0.924 | alpine habitats |
| Ranunculus lanuginosus | 0.649 | 0 / 0 | 0.698 | 0 / 0 | 0.692 | 0 / 0 | 0.835 | forests |
| Ranunculus montanus agg. | 0.86 | 0 / 0 | 0.839 | 0 / 0 | 0.83 | 0 / 0 | 0.859 | alpine habitats |
| Ranunculus nemorosus | 0.681 | 0 / 0 | 0.619 | 0 / 0 | 0.645 | 0 / 0 | 0.793 |  |
| Ranunculus polyanthemos s. str. | 0.886 | 0 / 0 | 0.76 | 0 / 0 | 0.819 | 0 / 0 | 0.969 | grasslands |
| Ranunculus repens | 0.66 | 0 / 0 | 0.626 | 0 / 0 | 0.578 | 0 / 0 | 0.809 |  |
| Reseda lutea | 0.842 | 0 / 0 | 0.928 | 0 / 0 | 0.865 | 0 / 0 | 0.943 | agricultural lands |
| Rhamnus cathartica | 0.657 | 0 / 0 | 0.699 | 0 / 0 | 0.537 | 1 / 0 | 0.835 |  |
| Rhinanthus alectorolophus s. lat. | 0.796 | 0 / 0 | 0.798 | 0 / 0 | 0.807 | 0 / 0 | 0.919 | grasslands |
| Rhinanthus glacialis | 0.764 | 0 / 0 | 0.814 | 0 / 0 | 0.761 | 0 / 0 | 0.895 |  |
| Rhinanthus minor | 0.774 | 0 / 0 | 0.769 | 0 / 0 | 0.702 | 0 / 0 | 0.877 | grasslands |
| Rhododendron hirsutum | 0.748 | 0 / 0 | 0.732 | 0 / 0 | 0.741 | 0 / 0 | 0.859 |  |
| Rhodothamnus chamaecistus | 0.768 | 0 / 0 | 0.828 | 0 / 0 | 0.693 | 0 / 0 | 0.902 | alpine habitats |
| Rhynchospora alba | 0.887 | 0 / 0 | 0.912 | 0 / 0 | 0.927 | 0 / 0 | 0.951 | grasslands |
| Robinia pseudacacia | 0.76 | 0 / 0 | 0.781 | 0 / 0 | 0.764 | 0 / 0 | 0.911 |  |
| Rosa arvensis | 0.67 | 0 / 0 | 0.714 | 0 / 0 | 0.661 | 0 / 0 | 0.853 |  |
| Rosa canina agg. | 0.693 | 0 / 0 | 0.618 | 0 / 0 | 0.601 | 0 / 0 | 0.811 |  |
| Rosa pendulina | 0.605 | 0 / 0 | 0.66 | 0 / 0 | 0.654 | 0 / 0 | 0.849 |  |
| Rubus caesius | 0.714 | 0 / 0 | 0.664 | 0 / 0 | 0.666 | 0 / 0 | 0.815 |  |
| Rubus fruticosus agg. | 0.673 | 0 / 0 | 0.645 | 0 / 0 | 0.631 | 0 / 0 | 0.787 | forests |
| Rubus idaeus | 0.63 | 0 / 0 | 0.618 | 0 / 0 | 0.612 | 0 / 0 | 0.786 | forests |
| Rubus saxatilis | 0.708 | 0 / 0 | 0.742 | 0 / 0 | 0.724 | 0 / 0 | 0.854 |  |
| Rumex acetosa | 0.823 | 0 / 0 | 0.788 | 0 / 0 | 0.771 | 0 / 0 | 0.875 | grasslands |
| Rumex alpestris | 0.872 | 0 / 0 | 0.823 | 0 / 0 | 0.822 | 0 / 0 | 0.916 |  |
| Rumex crispus | 0.649 | 0 / 0 | 0.706 | 0 / 0 | 0.739 | 0 / 0 | 0.867 |  |
| Rumex obtusifolius | 0.765 | 0 / 0 | 0.627 | 0 / 0 | 0.554 | 0 / 0 | 0.817 |  |
| Rumex scutatus | 0.744 | 0 / 0 | 0.812 | 0 / 0 | 0.78 | 1 / 0 | 0.94 | alpine habitats |
| Salix alba | 0.701 | 0 / 0 | 0.751 | 0 / 0 | 0.736 | 0 / 0 | 0.893 | forests |
| Salix alpina | 0.831 | 0 / 0 | 0.854 | 0 / 0 | 0.79 | 0 / 0 | 0.936 | alpine habitats |
| Salix appendiculata s. str. | 0.645 | 0 / 0 | 0.662 | 0 / 0 | 0.649 | 0 / 0 | 0.849 |  |
| Salix caprea | 0.559 | 0 / 1 | 0.581 | 0 / 0 | 0.52 | 0 / 2 | 0.76 |  |
| Salix cinerea s. str. | 0.579 | 0 / 0 | 0.742 | 0 / 0 | 0.578 | 2 / 0 | 0.873 |  |
| Salix eleagnos | 0.639 | 0 / 0 | 0.756 | 0 / 0 | 0.712 | 0 / 0 | 0.886 |  |
| Salix fragilis s. str. | 0.756 | 0 / 0 | 0.857 | 0 / 0 | 0.853 | 1 / 0 | 0.925 | forests |
| Salix myrsinifolia | 0.746 | 0 / 0 | 0.744 | 0 / 0 | 0.618 | 0 / 1 | 0.923 |  |
| Salix purpurea | 0.646 | 0 / 0 | 0.648 | 0 / 0 | 0.539 | 0 / 0 | 0.841 |  |
| Salix repens | 0.593 | 0 / 2 | 0.759 | 0 / 0 | 0.697 | 0 / 0 | 0.87 | grasslands |
| Salix reticulata | 0.874 | 0 / 0 | 0.886 | 0 / 0 | 0.872 | 0 / 0 | 0.937 | alpine habitats |
| Salix retusa s. str. | 0.885 | 0 / 0 | 0.897 | 0 / 0 | 0.887 | 0 / 0 | 0.938 | alpine habitats |
| Salix serpyllifolia | 0.919 | 0 / 0 | 0.912 | 0 / 0 | 0.915 | 0 / 0 | 0.963 | alpine habitats |
| Salvia glutinosa | 0.744 | 0 / 0 | 0.721 | 0 / 0 | 0.7 | 0 / 0 | 0.817 | forests |
| Salvia pratensis | 0.902 | 0 / 0 | 0.849 | 0 / 0 | 0.835 | 0 / 0 | 0.936 | grasslands |
| Salvia verticillata | 0.817 | 0 / 0 | 0.831 | 0 / 0 | 0.802 | 0 / 0 | 0.91 | grasslands |
| Sambucus ebulus | NA | 0 / 3 | 0.545 | 0 / 1 | NA | 2 / 1 | 0.737 | forests |
| Sambucus nigra | 0.696 | 0 / 0 | 0.638 | 0 / 0 | 0.645 | 0 / 0 | 0.792 | forests |
| Sambucus racemosa | 0.678 | 0 / 0 | 0.701 | 0 / 0 | 0.572 | 1 / 0 | 0.849 | forests |
| Sanguisorba minor | 0.906 | 0 / 0 | 0.855 | 0 / 0 | 0.804 | 0 / 0 | 0.939 | grasslands |
| Sanguisorba officinalis | 0.842 | 0 / 0 | 0.825 | 0 / 0 | 0.785 | 0 / 0 | 0.898 | grasslands |
| Sanicula europaea | 0.714 | 0 / 0 | 0.723 | 0 / 0 | 0.715 | 0 / 0 | 0.852 | forests |
| Saponaria pumila | 0.979 | 0 / 0 | 0.983 | 0 / 0 | 0.86 | 0 / 0 | 0.991 | alpine habitats |
| Saxifraga aizoides | 0.792 | 0 / 0 | 0.789 | 0 / 0 | 0.772 | 0 / 0 | 0.896 | alpine habitats |
| Saxifraga androsacea | 0.889 | 0 / 0 | 0.897 | 0 / 0 | 0.786 | 0 / 0 | 0.932 | alpine habitats |
| Saxifraga bryoides | 0.864 | 0 / 0 | 0.934 | 0 / 0 | 0.871 | 0 / 0 | 0.986 | alpine habitats |
| Saxifraga caesia | 0.864 | 0 / 0 | 0.888 | 0 / 0 | 0.852 | 0 / 0 | 0.936 | alpine habitats |
| Saxifraga moschata | 0.851 | 0 / 0 | 0.923 | 0 / 0 | 0.883 | 0 / 0 | 0.958 | alpine habitats |
| Saxifraga oppositifolia s. str. | 0.901 | 0 / 0 | 0.968 | 0 / 0 | 0.884 | 0 / 0 | 0.979 | alpine habitats |
| Saxifraga paniculata | 0.813 | 0 / 0 | 0.843 | 0 / 0 | 0.81 | 0 / 0 | 0.901 | alpine habitats |
| Saxifraga rotundifolia | 0.766 | 0 / 0 | 0.783 | 0 / 0 | 0.732 | 0 / 0 | 0.889 |  |
| Saxifraga sedoides | 0.927 | 0 / 0 | 0.953 | 0 / 0 | 0.94 | 0 / 0 | 0.979 | alpine habitats |
| Saxifraga stellaris | 0.785 | 0 / 0 | 0.825 | 0 / 0 | 0.797 | 0 / 0 | 0.91 | alpine habitats |
| Scabiosa columbaria s. str. | 0.749 | 0 / 0 | 0.762 | 0 / 0 | 0.703 | 0 / 0 | 0.906 | grasslands |
| Scabiosa lucida | 0.777 | 0 / 0 | 0.798 | 0 / 0 | 0.751 | 0 / 0 | 0.859 |  |
| Scabiosa ochroleuca | 0.848 | 0 / 0 | 0.841 | 0 / 0 | 0.824 | 0 / 0 | 0.957 | grasslands |
| Scheuchzeria palustris | 0.748 | 0 / 0 | 0.862 | 0 / 0 | 0.829 | 0 / 0 | 0.925 | grasslands |
| Schoenus ferrugineus | 0.915 | 0 / 0 | 0.903 | 0 / 0 | 0.895 | 0 / 0 | 0.941 | grasslands |
| Scirpus sylvaticus | 0.687 | 0 / 0 | 0.6 | 0 / 0 | 0.529 | 1 / 0 | 0.823 |  |
| Scorzonera humilis | 0.508 | 0 / 0 | 0.704 | 0 / 0 | 0.683 | 0 / 0 | 0.87 | grasslands |
| Scorzoneroides autumnalis | 0.768 | 0 / 0 | 0.723 | 0 / 0 | 0.676 | 0 / 0 | 0.93 |  |
| Scorzoneroides helvetica | 0.918 | 0 / 0 | 0.881 | 0 / 0 | 0.883 | 0 / 0 | 0.946 | alpine habitats |
| Scrophularia nodosa | 0.586 | 0 / 0 | 0.575 | 0 / 0 | 0.531 | 0 / 0 | 0.804 |  |
| Securigera varia | 0.804 | 0 / 0 | 0.829 | 0 / 0 | 0.754 | 0 / 0 | 0.925 |  |
| Sedum album | 0.69 | 0 / 0 | 0.618 | 0 / 0 | 0.6 | 0 / 1 | 0.895 |  |
| Sedum atratum | 0.776 | 0 / 0 | 0.832 | 0 / 0 | 0.795 | 0 / 0 | 0.913 | alpine habitats |
| Sedum sexangulare | 0.798 | 0 / 0 | 0.826 | 0 / 0 | 0.7 | 0 / 0 | 0.929 | grasslands |
| Selaginella selaginoides | 0.842 | 0 / 0 | 0.823 | 0 / 0 | 0.802 | 0 / 0 | 0.875 | alpine habitats |
| Selinum carvifolia | 0.772 | 0 / 0 | 0.82 | 0 / 0 | 0.776 | 0 / 0 | 0.932 | grasslands |
| Senecio abrotanifolius | 0.748 | 0 / 0 | 0.718 | 0 / 0 | 0.688 | 1 / 0 | 0.897 |  |
| Senecio incanus subsp. carniolicus | 0.939 | 0 / 0 | 0.95 | 0 / 0 | 0.948 | 0 / 0 | 0.982 | alpine habitats |
| Senecio jacobaea | 0.807 | 0 / 0 | 0.869 | 0 / 0 | 0.867 | 0 / 0 | 0.946 | grasslands |
| Senecio nemorensis agg. | 0.726 | 0 / 0 | 0.697 | 0 / 0 | 0.699 | 0 / 0 | 0.81 | forests |
| Senecio subalpinus | 0.818 | 0 / 0 | 0.81 | 0 / 0 | 0.701 | 0 / 0 | 0.92 |  |
| Senecio vulgaris | 0.545 | 0 / 0 | 0.611 | 0 / 0 | 0.533 | 0 / 0 | 0.895 |  |
| Serratula tinctoria | 0.918 | 0 / 0 | 0.912 | 0 / 0 | 0.906 | 0 / 0 | 0.959 | grasslands |
| Sesleria caerulea s. str. | 0.677 | 0 / 0 | 0.64 | 0 / 0 | 0.636 | 0 / 0 | 0.808 |  |
| Sesleria ovata | 0.941 | 0 / 0 | 0.917 | 0 / 0 | 0.902 | 0 / 0 | 0.969 | alpine habitats |
| Setaria pumila | 0.858 | 0 / 0 | 0.89 | 0 / 0 | 0.801 | 0 / 0 | 0.964 | agricultural lands |
| Silene acaulis s. lat. | 0.921 | 0 / 0 | 0.923 | 0 / 0 | 0.912 | 0 / 0 | 0.938 | alpine habitats |
| Silene dioica | 0.587 | 0 / 0 | 0.548 | 0 / 0 | 0.532 | 0 / 1 | 0.818 |  |
| Silene latifolia | 0.871 | 0 / 0 | 0.856 | 0 / 0 | 0.782 | 0 / 0 | 0.932 | agricultural lands |
| Silene nutans s. lat | 0.713 | 0 / 0 | 0.696 | 0 / 0 | 0.698 | 0 / 0 | 0.901 |  |
| Silene vulgaris | 0.693 | 0 / 0 | 0.602 | 0 / 0 | 0.558 | 0 / 0 | 0.824 |  |
| Sinapis alba | 0.919 | 0 / 0 | 0.883 | 0 / 0 | 0.812 | 0 / 0 | 0.963 | agricultural lands |
| Sinapis arvensis | 0.84 | 0 / 0 | 0.744 | 0 / 0 | 0.745 | 0 / 0 | 0.896 | agricultural lands |
| Solanum dulcamara | 0.614 | 0 / 0 | 0.666 | 0 / 0 | 0.685 | 1 / 0 | 0.832 |  |
| Solanum nigrum | 0.828 | 0 / 1 | 0.653 | 0 / 0 | 0.599 | 0 / 0 | 0.92 | agricultural lands |
| Solanum tuberosum | 0.931 | 0 / 0 | 0.892 | 0 / 0 | 0.739 | 0 / 0 | 0.96 | agricultural lands |
| Soldanella alpina | 0.869 | 0 / 0 | 0.875 | 0 / 0 | 0.853 | 0 / 0 | 0.899 | alpine habitats |
| Soldanella austriaca | 0.816 | 0 / 0 | 0.862 | 0 / 0 | 0.83 | 0 / 0 | 0.945 | alpine habitats |
| Soldanella pusilla | 0.77 | 0 / 0 | 0.891 | 0 / 0 | 0.812 | 0 / 0 | 0.938 | alpine habitats |
| Solidago gigantea | 0.742 | 0 / 0 | 0.779 | 0 / 0 | 0.744 | 0 / 0 | 0.893 |  |
| Solidago virgaurea | 0.652 | 0 / 0 | 0.625 | 0 / 0 | 0.585 | 0 / 0 | 0.776 |  |
| Sonchus arvensis | 0.673 | 0 / 0 | 0.77 | 0 / 0 | 0.513 | 0 / 0 | 0.942 | agricultural lands |
| Sonchus asper | 0.697 | 0 / 0 | 0.609 | 0 / 0 | 0.616 | 0 / 1 | 0.904 | agricultural lands |
| Sonchus oleraceus | 0.661 | 0 / 1 | 0.654 | 0 / 0 | 0.56 | 0 / 2 | 0.858 |  |
| Sorbus aria | 0.7 | 0 / 0 | 0.689 | 0 / 0 | 0.704 | 0 / 0 | 0.81 | forests |
| Sorbus aucuparia | 0.683 | 0 / 0 | 0.704 | 0 / 0 | 0.709 | 0 / 0 | 0.82 | forests |
| Sorbus torminalis | 0.939 | 0 / 0 | 0.962 | 0 / 0 | 0.919 | 0 / 0 | 0.98 | forests |
| Stachys alpina | 0.542 | 0 / 2 | 0.513 | 0 / 0 | NA | 1 / 2 | 0.774 |  |
| Stachys recta subsp. recta | 0.839 | 0 / 0 | 0.704 | 0 / 0 | 0.744 | 0 / 0 | 0.938 | grasslands |
| Stachys sylvatica | 0.692 | 0 / 0 | 0.705 | 0 / 0 | 0.69 | 0 / 0 | 0.787 | forests |
| Stellaria graminea | 0.74 | 0 / 0 | 0.76 | 0 / 0 | 0.706 | 1 / 0 | 0.889 | grasslands |
| Stellaria holostea | 0.904 | 0 / 0 | 0.937 | 0 / 0 | 0.93 | 0 / 0 | 0.963 | forests |
| Stellaria media agg. | 0.791 | 0 / 0 | 0.788 | 0 / 0 | 0.767 | 0 / 0 | 0.829 |  |
| Stellaria nemorum s. str. | 0.703 | 0 / 0 | 0.74 | 0 / 0 | 0.722 | 0 / 0 | 0.906 | forests |
| Succisa pratensis | 0.811 | 0 / 0 | 0.782 | 0 / 0 | 0.782 | 0 / 0 | 0.872 | grasslands |
| Symphyotrichum lanceolatum | 0.902 | 0 / 0 | 0.953 | 0 / 0 | 0.929 | 0 / 0 | 0.979 | grasslands |
| Symphytum officinale s. str. | 0.786 | 0 / 0 | 0.75 | 0 / 0 | 0.675 | 0 / 0 | 0.871 |  |
| Symphytum tuberosum s. lat. | 0.678 | 0 / 0 | 0.776 | 0 / 0 | 0.715 | 0 / 0 | 0.856 | forests |
| Tanacetum corymbosum subsp. corymbosum | 0.868 | 0 / 0 | 0.916 | 0 / 0 | 0.844 | 0 / 0 | 0.942 | forests |
| Taraxacum sect. Ruderalia | 0.708 | 0 / 0 | 0.678 | 0 / 0 | 0.665 | 0 / 0 | 0.764 |  |
| Taxus baccata | 0.66 | 0 / 0 | 0.785 | 0 / 0 | 0.719 | 0 / 0 | 0.857 | forests |
| Teucrium chamaedrys | 0.759 | 0 / 0 | 0.683 | 0 / 0 | 0.729 | 0 / 0 | 0.855 |  |
| Teucrium montanum | 0.738 | 0 / 0 | 0.878 | 0 / 0 | 0.698 | 0 / 0 | 0.932 |  |
| Thalictrum aquilegiifolium | 0.613 | 0 / 1 | 0.626 | 0 / 0 | 0.502 | 0 / 1 | 0.806 |  |
| Thalictrum minus s. lat. | 0.85 | 0 / 0 | 0.779 | 0 / 0 | 0.732 | 0 / 0 | 0.913 |  |
| Thelypteris limbosperma | 0.723 | 0 / 0 | 0.696 | 0 / 0 | 0.655 | 0 / 0 | 0.851 | forests |
| Thesium alpinum | 0.678 | 0 / 0 | 0.741 | 0 / 0 | 0.697 | 0 / 0 | 0.85 | alpine habitats |
| Thlaspi arvense | 0.851 | 0 / 0 | 0.824 | 0 / 0 | 0.77 | 0 / 0 | 0.912 | agricultural lands |
| Thymus praecox agg. | 0.786 | 0 / 0 | 0.802 | 0 / 0 | 0.78 | 0 / 0 | 0.88 | alpine habitats |
| Thymus pulegioides | 0.716 | 0 / 0 | 0.732 | 0 / 0 | 0.712 | 0 / 0 | 0.863 | grasslands |
| Tilia cordata | 0.758 | 0 / 0 | 0.786 | 0 / 0 | 0.78 | 0 / 0 | 0.86 | forests |
| Tilia platyphyllos | 0.777 | 0 / 0 | 0.807 | 0 / 0 | 0.807 | 0 / 0 | 0.87 | forests |
| Tofieldia calyculata | 0.601 | 0 / 0 | 0.635 | 0 / 0 | 0.623 | 0 / 0 | 0.758 |  |
| Tragopogon dubius | 0.952 | 0 / 0 | 0.893 | 0 / 0 | 0.931 | 0 / 0 | 0.966 |  |
| Tragopogon orientalis | 0.875 | 0 / 0 | 0.82 | 0 / 0 | 0.77 | 0 / 0 | 0.881 | grasslands |
| Traunsteinera globosa | 0.698 | 0 / 0 | 0.884 | 0 / 0 | 0.647 | 2 / 0 | 0.953 |  |
| Trichophorum alpinum | 0.516 | 0 / 0 | 0.83 | 0 / 0 | 0.805 | 0 / 0 | 0.886 | grasslands |
| Trichophorum cespitosum | 0.808 | 0 / 0 | 0.868 | 0 / 0 | 0.842 | 0 / 0 | 0.902 | grasslands |
| Trifolium badium | 0.585 | 0 / 0 | 0.743 | 0 / 0 | 0.512 | 0 / 0 | 0.857 | alpine habitats |
| Trifolium campestre | 0.78 | 0 / 0 | 0.804 | 0 / 0 | 0.647 | 0 / 0 | 0.919 |  |
| Trifolium dubium | 0.661 | 0 / 0 | 0.777 | 0 / 0 | 0.619 | 0 / 0 | 0.891 | grasslands |
| Trifolium hybridum | 0.675 | 0 / 0 | 0.724 | 0 / 0 | 0.6 | 0 / 1 | 0.902 |  |
| Trifolium medium | 0.722 | 0 / 0 | 0.642 | 0 / 0 | 0.554 | 0 / 0 | 0.861 | grasslands |
| Trifolium montanum | 0.783 | 0 / 0 | 0.839 | 0 / 0 | 0.83 | 0 / 0 | 0.896 | grasslands |
| Trifolium pratense | 0.741 | 0 / 0 | 0.662 | 0 / 0 | 0.664 | 0 / 0 | 0.796 |  |
| Trifolium repens | 0.797 | 0 / 0 | 0.769 | 0 / 0 | 0.731 | 0 / 0 | 0.825 |  |
| Tripleurospermum inodorum | 0.904 | 0 / 0 | 0.876 | 0 / 0 | 0.866 | 0 / 0 | 0.935 | agricultural lands |
| Trisetum alpestre | 0.849 | 0 / 0 | 0.847 | 0 / 0 | 0.767 | 0 / 0 | 0.921 | alpine habitats |
| Trisetum flavescens | 0.839 | 0 / 0 | 0.797 | 0 / 0 | 0.773 | 0 / 0 | 0.863 | grasslands |
| Trollius europaeus | 0.644 | 0 / 0 | 0.583 | 0 / 0 | 0.618 | 0 / 0 | 0.776 |  |
| Tussilago farfara | 0.539 | 0 / 0 | NA | 0 / 3 | NA | 0 / 3 | 0.934 |  |
| Ulmus glabra | 0.744 | 0 / 0 | 0.741 | 0 / 0 | 0.752 | 0 / 0 | 0.852 | forests |
| Ulmus minor | 0.83 | 0 / 0 | 0.716 | 0 / 0 | 0.719 | 0 / 0 | 0.94 | forests |
| Urtica dioica | 0.624 | 0 / 0 | 0.551 | 0 / 0 | 0.565 | 0 / 0 | 0.766 |  |
| Vaccinium gaultherioides | 0.783 | 0 / 0 | 0.834 | 0 / 0 | 0.85 | 0 / 0 | 0.921 | alpine habitats |
| Vaccinium myrtillus | 0.552 | 0 / 0 | 0.551 | 0 / 0 | 0.549 | 0 / 0 | 0.708 |  |
| Vaccinium oxycoccos s. str. | 0.818 | 0 / 0 | 0.855 | 0 / 0 | 0.813 | 0 / 0 | 0.895 | grasslands |
| Vaccinium uliginosum s. str. | 0.754 | 0 / 0 | 0.842 | 0 / 0 | 0.792 | 0 / 0 | 0.881 | grasslands |
| Vaccinium vitis-idaea | 0.631 | 0 / 0 | 0.63 | 0 / 0 | 0.632 | 0 / 0 | 0.751 |  |
| Valeriana celtica subsp. norica | 0.95 | 0 / 0 | 0.952 | 0 / 0 | 0.88 | 0 / 0 | 0.977 | alpine habitats |
| Valeriana dioica | 0.725 | 0 / 0 | 0.679 | 0 / 0 | 0.632 | 0 / 0 | 0.794 | grasslands |
| Valeriana elongata | 0.887 | 0 / 0 | 0.91 | 0 / 0 | 0.91 | 0 / 0 | 0.958 | alpine habitats |
| Valeriana montana | 0.729 | 0 / 0 | 0.765 | 0 / 0 | 0.666 | 0 / 0 | 0.875 |  |
| Valeriana officinalis s. lat. | 0.57 | 0 / 0 | 0.607 | 0 / 0 | 0.587 | 0 / 0 | 0.803 |  |
| Valeriana saxatilis | 0.636 | 0 / 0 | 0.713 | 0 / 0 | 0.727 | 0 / 0 | 0.843 |  |
| Valeriana tripteris | 0.713 | 0 / 0 | 0.733 | 0 / 0 | 0.7 | 0 / 0 | 0.866 | forests |
| Valerianella locusta | 0.823 | 0 / 0 | 0.8 | 0 / 0 | 0.54 | 0 / 1 | 0.964 |  |
| Veratrum album s. lat. | 0.61 | 0 / 0 | 0.582 | 0 / 0 | 0.553 | 0 / 0 | 0.793 |  |
| Veronica alpina | 0.859 | 0 / 0 | 0.875 | 0 / 0 | 0.814 | 0 / 0 | 0.917 | alpine habitats |
| Veronica aphylla | 0.695 | 0 / 0 | 0.881 | 0 / 0 | 0.777 | 0 / 0 | 0.91 | alpine habitats |
| Veronica arvensis | 0.832 | 0 / 0 | 0.783 | 0 / 0 | 0.741 | 0 / 0 | 0.876 |  |
| Veronica beccabunga | 0.538 | 0 / 1 | 0.548 | 0 / 0 | NA | 1 / 1 | 0.814 |  |
| Veronica chamaedrys agg. | 0.884 | 0 / 0 | 0.892 | 0 / 0 | 0.881 | 0 / 0 | 0.976 | grasslands |
| Veronica chamaedrys s. str. | 0.686 | 0 / 0 | 0.643 | 0 / 0 | 0.632 | 0 / 0 | 0.819 |  |
| Veronica hederifolia agg. | 0.855 | 0 / 0 | 0.835 | 0 / 0 | 0.812 | 1 / 0 | 0.93 | agricultural lands |
| Veronica montana | 0.529 | 0 / 0 | 0.732 | 0 / 0 | 0.652 | 0 / 0 | 0.86 | forests |
| Veronica officinalis | 0.617 | 0 / 0 | 0.64 | 0 / 0 | 0.627 | 0 / 0 | 0.828 |  |
| Veronica persica | 0.916 | 0 / 0 | 0.873 | 0 / 0 | 0.877 | 0 / 0 | 0.921 | agricultural lands |
| Veronica polita | 0.931 | 0 / 0 | 0.917 | 0 / 0 | 0.882 | 1 / 0 | 0.956 | agricultural lands |
| Veronica serpyllifolia | 0.726 | 0 / 0 | 0.754 | 0 / 0 | 0.709 | 0 / 0 | 0.846 |  |
| Viburnum lantana | 0.666 | 0 / 0 | 0.673 | 0 / 0 | 0.663 | 0 / 0 | 0.845 | forests |
| Viburnum opulus | 0.741 | 0 / 0 | 0.75 | 0 / 0 | 0.729 | 0 / 0 | 0.829 |  |
| Vicia angustifolia | 0.84 | 0 / 0 | 0.74 | 0 / 0 | 0.692 | 0 / 0 | 0.935 |  |
| Vicia cracca s. strictiss. | 0.663 | 0 / 0 | 0.609 | 0 / 0 | 0.564 | 0 / 0 | 0.809 | grasslands |
| Vicia hirsuta | 0.764 | 0 / 0 | 0.672 | 0 / 0 | 0.73 | 0 / 0 | 0.897 |  |
| Vicia sepium | 0.711 | 0 / 0 | 0.634 | 0 / 0 | 0.514 | 0 / 0 | 0.842 |  |
| Vicia tenuifolia | 0.548 | 0 / 1 | 0.628 | 0 / 1 | NA | 1 / 1 | 0.851 |  |
| Vicia tetrasperma s. str. | 0.663 | 0 / 0 | 0.645 | 0 / 0 | 0.636 | 0 / 0 | 0.906 |  |
| Vinca minor | 0.706 | 0 / 0 | 0.685 | 0 / 0 | 0.617 | 0 / 0 | 0.89 | forests |
| Vincetoxicum hirundinaria | 0.674 | 0 / 0 | 0.689 | 0 / 0 | 0.656 | 0 / 0 | 0.845 |  |
| Viola alba | 0.973 | 0 / 0 | 0.991 | 0 / 0 | 0.932 | 0 / 0 | 0.978 | forests |
| Viola arvensis | 0.924 | 0 / 0 | 0.876 | 0 / 0 | 0.874 | 0 / 0 | 0.92 | agricultural lands |
| Viola biflora | 0.796 | 0 / 0 | 0.763 | 0 / 0 | 0.73 | 0 / 0 | 0.861 | alpine habitats |
| Viola canina | 0.553 | 0 / 0 | 0.61 | 0 / 0 | 0.598 | 1 / 0 | 0.888 | grasslands |
| Viola hirta | 0.753 | 0 / 0 | 0.709 | 0 / 0 | 0.662 | 0 / 0 | 0.867 |  |
| Viola mirabilis | 0.716 | 0 / 0 | 0.732 | 0 / 0 | 0.747 | 0 / 0 | 0.921 | forests |
| Viola palustris | 0.755 | 0 / 0 | 0.807 | 0 / 0 | 0.797 | 0 / 0 | 0.842 | grasslands |
| Viola reichenbachiana | 0.705 | 0 / 0 | 0.701 | 0 / 0 | 0.671 | 0 / 0 | 0.826 | forests |
| Viola riviniana | 0.683 | 0 / 0 | 0.736 | 0 / 0 | 0.709 | 1 / 0 | 0.873 | forests |
| Willemetia stipitata | 0.775 | 0 / 0 | 0.791 | 0 / 0 | 0.792 | 0 / 0 | 0.846 | grasslands |

**Table S3.** Assumptions underlying the ABM with regard to yields, prices, subsidies, income, working hours, workload, extreme events and the national parks’ decisions concerning alpine pastures.

| **SCENARIOS** | **BAU** | **SSP1** | **SSP5** |
| --- | --- | --- | --- |
| **Yields on crop- and grassland**^[[2]](#footnote-2)^ | 15-20 % increase | 15-20 % increase | 20-30 % increase |
| **Agricultural product prices** | index adjusted | increase for low input products and energy plants | index adjusted |
| **Agricultural subsidies** | index adjusted | increase for low input products and energy plants by year 2020 | strong decrease by year 2020; no subsidies any more by year 2026 |
| **Variable costs** | index adjusted | index adjusted | moderate increase |
| **Non-agricultural income** | index adjusted | medium increase | minor increase in peri-urban areas, minor decrease in rural areas |
| **Agricultural working hours per ha or livestock** | constant | constant | minor decrease |
| **Maximum of accepted work load** | constant | lower | constant |
| **Minimum of required agricultural income** | index adjusted | minor increase | strong increase |
| **Extreme events** | low probability of occurrence, only few sub regions^[[3]](#footnote-3)^ affected; moderate reduction of standard output | very low probability of occurrence, only one affected sub region^3^, minor reduction of standard output | high probability of occurrence, more affected sub regions^3^; strong reduction of standard output |
| **National park** | National parks lease every vacant alpine pasture | National parks lease every vacant alpine pasture | National parks stop leasing alpine pastures |
|  |  |  |  |

**Table S4:** Description of the agent-based model according to the ODD protocol guidelines (Grimm *et al.*, 2006; Grimm *et al.*, 2010; Müller *et al.*, 2013).

| **Overview** | **Purpose** | The purpose of this model is to simulate changes in land-use patterns in the Enns valley as a result of farmers response to three scenarios with changing socio-economic and climate conditions. |
| --- | --- | --- |
|  | **Entities** | **Farms:** based on the three essential characteristics 75 farm agent variations exist  - three farm types: cash crop, processing and livestock - five farming styles: yield optimizer, support optimizer, traditionalist, idealist, innovative - five intensity levels: low-input, 3 intermediate levels distinguished by the Austrian Agri-Environmental Programme, intense production **National park:** representing the two national parks "Kalkalpen" and "Gesäuse" **Rent market:** technical agent, holds abandoned areas and acts as functional link between agents and areas **Patches:** individual parcels of land assigned to agents and differing by crop cultures and intensity crop cultures: cereals, non-cereals, energy plants, meadows, pastures, fallow, forest and other five intensity levels: low-input, 3 intermediate levels distinguished by the Austrian Agri-Environmental Programme, intense production |
|  | **Spatial and temporal scales** | The model is run for a cycle of 36 years simulating the period from 2014-2050. The biophysical environment for land use simulations consists of 18,772 agricultural patches and 429,983 patches representing forest areas for simulations in the forest module. Each scenario is run in a Monte-Carlo-Simulation of 100 runs. |
|  | **Process overview and scheduling** | For process overview see Appendix Figure S2 |
| **Design concepts** | **Theoretical and Empirical Background** | We want to explore the anticipated systemic feedbacks between 1) climate change, 2) land owner’s decisions on land use, 3) land-use change, and then from that 4) changes in biodiversity patterns during the upcoming decades until 2050 in a regional context, integrating a broad range of land use practices and intensity gradients. |
|  | **Individual Decision Making** | Farmers want to stay within thresholds for time use and income to maintain "happiness". **Time threshold:** 1,800 hours per year (baseline assumption) **fixed** **Income threshold:** 20,000 € total income (agricultural + non-agricultural) (baseline assumption) **relative Income threshold:** mean agricultural income per hour - 1 standard deviation **Unhappiness:** if farms violate the threshold limits (exceed time and/or deceed income), they are unhappy. Each state of unhappiness increases the probability of termination of farming. The set of possible actions depend on whether and which of the thresholds are violated as well as the farm type. Within a certain set, decisions are selected based on probabilities that depend on the respective farming style. Probabilities were assigned based on 35 semi-structured qualitative interviews with regional farmers. |
|  | **Adaption** | Depending on the outcome of the 2x2-happiness matrix with income and time use farms decide upon ten possible actions in order to recover or maintain their happiness: - Nothing - Termination (land to the rent market) - Intensification (increase of 1 intensity level) - Extensification (decrease of 1 intensity level) - Expansion (acquire 1 patch from the rent market) - Reduction (fire farm workers, put 1 patch to the rent market) - Land use change (exchange among cereals, non-cereal crops and energy plants; meadows and   pastures) - Low-Input (direct switch to lowest intensity level) - Hire farm worker (180 hours/year) - Afforestation (of grassland areas) |
|  | **Learning** | Farms do not change their behaviour over time. |
|  | **Individual Prediction** | Farms do not make predictions. |
|  | **Individual Sensing** | Farmers calculate themselves their income and their working hours based on external input factors such as prices, subsidies, wages. They can sense the fixed income threshold which is the same for all farms and the relative income threshold that is calculated for each of the three farming types. |
|  | **Interaction** | Farms and the national park do not interact directly. Exchanges of areas only happen via the intermediate, technical agent "rent market". |
|  | **Collectives** | The farms do not form any form of collectives. |
|  | **Heterogeneity** | The agents differ by farming type, farming style and farm intensity level. These characteristics and their happiness state have an impact on the decision-making and the set of possible actions among which farms choose. |
|  | **Stochasticity** | Stochasticity was an important factor for the allocation of variables with high uncertainty. Farming styles are allocated randomly. Socio-demographic factors (age, civil status, succession) are allocated based on regional statistics and national survey data. The differentiation between meadows and pastures is based on the statistical analysis of the grassland data set. |
|  | **Observation** | Farms and patches track important information as e.g. happiness, decisions, intensity level or habitat individually. During the model runs there are also global variables such as number of active/passive farms, happiness state of active farms, sum over area and habitat that track collective data. |
| **Implemen-tation Details** | **Initialization** | The model is implemented in Netlogo 6.0.1 and the Forest module in Python 2.6.  For each run, the model newly initializes the starting conditions in 2014, with variations in farming style, socio-demographic factors and allocation of meadows, pastures and fallow areas. Varying factors are based on statistics or if not available on random normal distributed values. |
|  | **Input data** | **Quantitative data:** IACS Austria (INVEKOS provided by the Austrian Ministry of Sustainability and Tourism) 2014 GIS map of spatially explicit farm data Statistics Austria (regional socio-economic and agricultural data)  Farm Account Data (LBG Accounting Report) Contribution margins and calculation data (Calculator-Tool form the ministry of sustainability and tourism https://idb.awi.bmlfuw.gv.at/) Standardarbeitszeitbedarf in der österreichischen Landwirtschaft (Standard working time requirement in the Austrian Agriculture) **Qualitative data:** 35 interviews with regional farmers and experts |
|  | **Submodels** | ABM: simulates land user decisions Forest module: simulates forest management decisions for forest areas (existing in 2014) |

**Table S5:** The minimum (*min*), maximum (*max*), mean values (*mean*), and the standard deviation (*sd*) of the four bioclimatic variables used as predictors in the SDMs: minimum temperature of the coldest month (*BIO6*, unit: °C), temperature annual range (*BIO7*, unit: °C), precipitation seasonality (*BIO15*, unit: percent), precipitation of the warmest quarter (*BIO18*, unit: millimetres). Values are for the study region as depicted in Fig. 1a and were calculated for a) current climate, as well as for the three future climate scenarios considered: b) RCP2.6, c) RCP4.5 and d) RCP8.5.

| 1. **CURRENT** | *BIO6* | *BIO7* | *BIO15* | *BIO18* |
| --- | --- | --- | --- | --- |
| *min* | -12.2 | 23.1 | 17 | 273 |
| *max* | -4.5 | 30.6 | 37 | 717 |
| *mean* | -7.337 | 28.822 | 25.393 | 462.482 |
| *sd* | 1.468 | 1.249 | 4.094 | 73.135 |
|  |  |  |  |  |
| 1. **RCP2.6** | *BIO6* | *BIO7* | *BIO15* | *BIO18* |
| *min* | -10.6 | 23 | 20 | 258 |
| *max* | -2 | 29.7 | 38 | 769 |
| *mean* | -4.939 | 27.756 | 26.544 | 481.879 |
| *sd* | 1.505 | 1.206 | 3.298 | 85.031 |
|  |  |  |  |  |
| 1. **RCP4.5** | *BIO6* | *BIO7* | *BIO15* | *BIO18* |
| *min* | -10.3 | 23.6 | 20 | 263 |
| *max* | -0.9 | 29.5 | 37 | 732 |
| *mean* | -4.373 | 27.947 | 26.337 | 462.900 |
| *sd* | 1.723 | 0.976 | 3.391 | 74.583 |
|  |  |  |  |  |
| 1. **RCP8.5** | *BIO6* | *BIO7* | *BIO15* | *BIO18* |
| *min* | -7.6 | 21 | 16 | 271 |
| *max* | -9 | 29.5 | 32 | 724 |
| *mean* | -3.509 | 27.128 | 22.126 | 449.181 |
| *sd* | 1.125 | 1.513 | 3.210 | 70.974 |

**Table S6:** Results of a generalized linear mixed-effects model relating the exposure (i.e. the spatial displacement of ranges) to climate change scenario, land-use change scenario, and their interaction. Lower AIC (Akaike Information Criterion) values indicate better models. *R²_m_* and *R²_c_* are the marginal and conditional *R²*-values of the model. The interaction term RCP8.5 : SSP5 could not be calculated due to rank deficiency of the model matrix.

| **Predictors** | **Estimate** | **Std. error** | ***p*-value** | **AIC** | ***R^2^_m_*** | ***R^2^_c_*** |
| --- | --- | --- | --- | --- | --- | --- |
| Climate change scenario* Land-use change scenario | |  |  | 39575 | 0.349 | 0.773 |
| RCP2.6 | 5.384 | 0.117 | <0.001 |  |  |  |
| RCP4.5 | 5.691 | 0.118 | <0.001 |  |  |  |
| RCP8.5 | 5.506 | 0.095 | <0.001 |  |  |  |
| BAU | 0.815 | 0.128 | <0.001 |  |  |  |
| SSP1 | 0.489 | 0.134 | <0.001 |  |  |  |
| SSP5 | 0.983 | 0.071 | <0.001 |  |  |  |
| RCP2.6 : BAU | 0.372 | 0.145 | 0.010 |  |  |  |
| RCP4.5 : BAU | 0.285 | 0.145 | 0.050 |  |  |  |
| RCP8.5 : BAU | 0.387 | 0.128 | 0.002 |  |  |  |
| RCP2.6 : SSP1 | 0.413 | 0.151 | 0.006 |  |  |  |
| RCP4.5 : SSP1 | 0.322 | 0.151 | 0.034 |  |  |  |
| RCP8.5 : SSP1 | 0.474 | 0.135 | <0.001 |  |  |  |
| RCP2.6 : SSP5 | 0.112 | 0.099 | <0.001 |  |  |  |
| RCP4.5 : SSP5 | -0.073 | 0.099 | 0.256 |  |  |  |
| Excluding |  |  |  |  |  |  |
| Climate change scenario | |  |  | 62705 | 0.003 | 0.461 |
| Land-use change scenario | |  |  | 40527 | 0.342 | 0.760 |
| Climate change scenario : Land-use change scenario | | |  | 39577 | 0.348 | 0.773 |

**References**

Choe H, Thorne JH, Hijmans R, Kim J, Kwon H, Seo C (2017) Meta-corridor solutions for climate-vulnerable plant species groups in South Korea. Journal of Applied Ecology*,* **54**, 1742-1754.

Grimm V, Berger U, Bastiansen F *et al.* (2006) A standard protocol for describing individual-based and agent-based models. Ecological Modelling*,* **198**, 115-126.

Grimm V, Berger U, DeAngelis DL, Polhill JG, Giske J, Railsback SF (2010) The ODD protocol: A review and first update. Ecological Modelling*,* **221**, 2760-2768.

Müller B, Bohn F, Dreßler G *et al.* (2013) Describing human decisions in agent-based models - ODD+D, an extension of the ODD protocol. Environmental Modelling and Software*,* **48**, 37-48.

Office of the State of Upper Austria (1993-2013) Compiled data of the Biotope Mapping Upper Austria.

Pascher K, Moser D, Dullinger S *et al.* (2011) Setup, efforts and practical experiences of a monitoring program for genetically modified plants - an Austrian case study for oilseed rape and maize. Environmental Sciences Europe*,* **23**, 12.

Willner W, Berg C, Heiselmayer P (2012) Austrian vegetation database. Biodivers Ecol*,* **4**.

1. Models failed to run [↑](#footnote-ref-1)
2. Erb KH, Lauk C, Kastner T, Mayer A, Theurl MC, Haberl H (2016) Exploring the biophysical option space for feeding the world without deforestation. Nature Communications, 7. [↑](#footnote-ref-2)
3. n=27 [↑](#footnote-ref-3)
